# A CD138^+^ tumor-associated macrophage/Siglec-F^+^ neutrophil feed-forward loop promotes immune evasion in pancreatic cancer

**DOI:** 10.1172/JCI199516

**Published:** 2026-03-12

**Authors:** Chao Wang, Qi Zhang, Jinyan Huang, Fangyu Lin, Danyang Zhao, Youling Mu, Junshuo Tong, Jinping Li, Yingjiqiong Liang, Tao Zeng, Fukang Shi, Hang Shen, Tingting Lu, Tingbo Liang

**Affiliations:** 1 Zhejiang Provincial Key Laboratory of Pancreatic Disease,; 2 Department of Hepatobiliary and Pancreatic Surgery, and; 3 Ministry of Education’s Joint International Research Laboratory of Pancreatic Diseases, The First Affiliated Hospital, Zhejiang University School of Medicine, Hangzhou, China.; 4 Zhejiang Clinical Research Center of Hepatobiliary and Pancreatic Diseases, Hangzhou, China.; 5 The Innovation Center for the Study of Pancreatic Diseases of Zhejiang Province, Hangzhou, China.; 6 Zhejiang University Cancer Center, Hangzhou, China.; 7 Bio-X Institutes, Key Laboratory for the Genetics of Developmental and Neuropsychiatric Disorders (Ministry of Education), Shanghai Jiao Tong University, Shanghai, China.

**Keywords:** Gastroenterology, Immunology, Oncology, Cancer immunotherapy, Macrophages, Neutrophils

## Abstract

Immune evasion is a major obstacle in pancreatic cancer therapy. Recent data implicate proinflammatory macrophages in the progression of pancreatic ductal adenocarcinoma (PDAC) and its therapeutic response. However, whether or which of the proinflammatory macrophage subtypes play a crucial role in the immune escape of PDAC remains unclear. Here, we identify a population of CD138^+^ tumor-associated macrophages (TAMs), characterized by their proinflammatory and neutrophil-chemotactic activity, which undergo significant expansion in both patients with PDAC and mouse models. These cells are elicited by a local synergy between IL-34/syndecan-1 and PGE_2_/EP2 signaling and are associated with immune evasion and poor clinical outcomes in patients, while also promoting immune escape and disease progression in mouse models. Mechanistically, CD138^+^ TAMs establish a feed-forward loop with immunosuppressive Siglec-F^+^ neutrophils, which exhibit elevated PGE_2_ expression, via the secretion of SAA3 and CXCL1. Targeting CD138^+^ TAMs by disrupting IL-34/syndecan-1 signaling with anti–IL-34 neutralizing antibodies significantly suppressed PDAC progression, especially when combined with anti–PD-1 antibodies. Together, our study elucidates a CD138^+^ TAM/Siglec-F^+^ neutrophil axis that drives immune escape in PDAC and proposes a therapeutic strategy that integrates IL-34/syndecan-1 signaling blockade with anti–PD-1 immunotherapy for the treatment of PDAC.

## Abstract 2

Targeting the CD138^+ ^tumor-associated macrophage-Siglec-F^+ ^neutrophil axis prevents immune evasion and enhances therapeutic responses to anti-PD-1 immunotherapy in pancreatic cancer.

## Graphical abstract

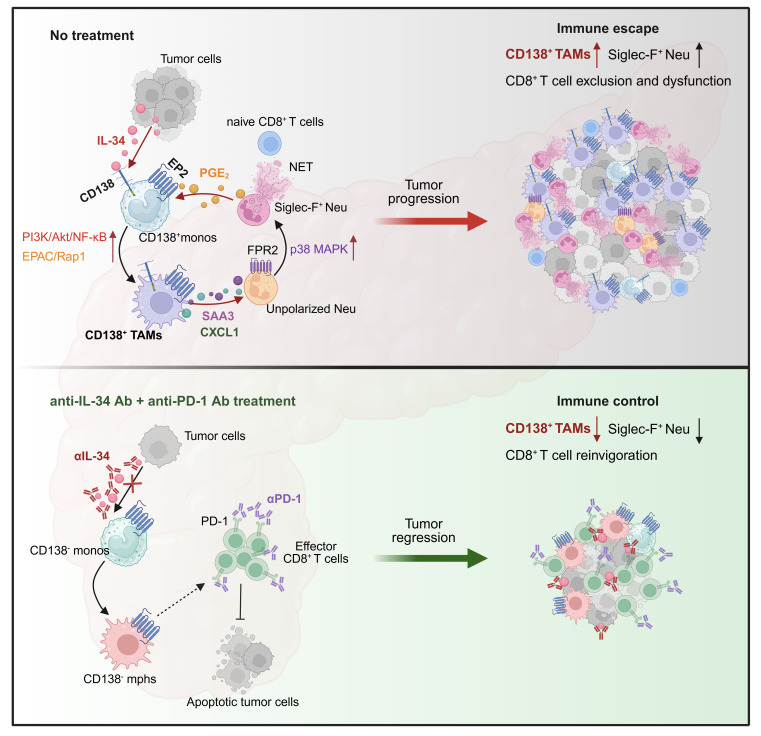

## Introduction

Pancreatic cancer, predominantly pancreatic ductal adenocarcinoma (PDAC), is recognized as one of the most aggressive solid malignancies, exhibiting nearly uniform mortality rates and ranking as the third leading cause of cancer-related deaths in the United States (1). PDAC is notoriously resistant to current therapies, including immunotherapy, primarily due to its immunosuppressive tumor microenvironment (TME) (2). Tumor-associated macrophages (TAMs), which are abundant within the PDAC TME, are believed to significantly influence tumor immune evasion (3), although it remains undetermined which TAM subset plays a pivotal role. Traditionally, proinflammatory macrophages have been regarded as antitumorigenic, characterized by an M1-like phenotype (4). Nonetheless, these macrophages harbor considerable heterogeneity and functional plasticity during disease progression and therapy (5). IL-1β^+^ TAMs trigger pathogenic inflammation, fueling PDAC development (6). Inflammatory macrophage-derived granulin and CXCL3 facilitate the generation of myofibroblasts, thereby driving tumor metastasis (7, 8). Conversely, upon exposure to radiotherapy, targeted therapy, and/or immunotherapy, the proinflammatory polarization of macrophages plays a crucial role in reactivating antitumor immunity (9–11). However, whether or which of the proinflammatory macrophage subtypes play a crucial role during the immune evasion of PDAC is still unclear, as is the manner in which local signals elicit their functions.

Previous studies have reported interactions between TAMs and neutrophils within the TME across several cancer types (12, 13). Mesothelin secretion by metastatic pancreatic cancer cells induces the expression of S100A9 in macrophages, which leads to increased neutrophil infiltration into the lungs and the formation of neutrophil extracellular traps (NETs), thereby supporting tumor metastasis (14). Furthermore, dual targeting of CCR2^+^ TAMs and CXCR2^+^ neutrophils improves antitumor immunity compared with either strategy alone, indicating a compensatory response from the alternative myeloid subset (15). However, the interactions between TAMs and neutrophils within the primary tumor of PDAC and their roles in tumor immune evasion remain unclear. A better understanding of these interactions is needed to facilitate the rational targeting of TAMs to overcome immune evasion in PDAC.

IL-34, a short-chain helical hematopoietic cytokine, plays a crucial role in regulating the differentiation, proliferation, and survival of myeloid cells (16, 17). It is increasingly evident that IL-34 significantly influences the immune escape and progression of several cancer types by inducing M2 polarization of TAMs (17, 18). Despite this, the specific roles of IL-34 in PDAC remain poorly understood. Syndecan-1, also known as CD138, serves as a functional receptor for IL-34; upon engagement, it stimulates the migration of myeloid cells and modulates the activation of CSF1R (19). Recent research has revealed that syndecan-1 localized on the surface of pancreatic cancer cells regulates macropinocytosis, an essential metabolic pathway that supports tumor growth (20). Nevertheless, the regulatory effects of IL-34/syndecan-1 signaling on the phenotype and function of myeloid cells within the TME as well as the impact of this process on tumor immune evasion are still undefined.

In this study, we delved into the influence of TAMs on the immune escape of PDAC, uncovering the expansion of a population of CD138^+^ TAMs in both PDAC patients and mouse models. This unique subset of TAMs exhibited proinflammatory and neutrophil-chemotactic activity. Their generation was collaboratively driven by IL-34/syndecan-1 and PGE_2_/EP2 signaling, establishing a feed-forward loop with immunosuppressive PGE_2_-expressing Siglec-F^+^ neutrophils through the secretion of SAA3 and CXCL1, thereby contributing to tumor immune evasion. Notably, we demonstrated that targeting CD138^+^ TAMs via the blockade of IL-34/syndecan-1 signaling effectively impeded PDAC progression, particularly when combined with anti–PD-1 antibodies. Our findings underscore the crucial roles of CD138^+^ TAMs in facilitating tumor immune evasion and provide valuable insights for developing combination therapy strategies that target these cells in conjunction with anti–PD-1 immunotherapy for the effective treatment of PDAC.

## Results

### A CD138^+^ TAM subset is identified and associated with immune evasion and poor outcomes in PDAC.

**Figure 1:**
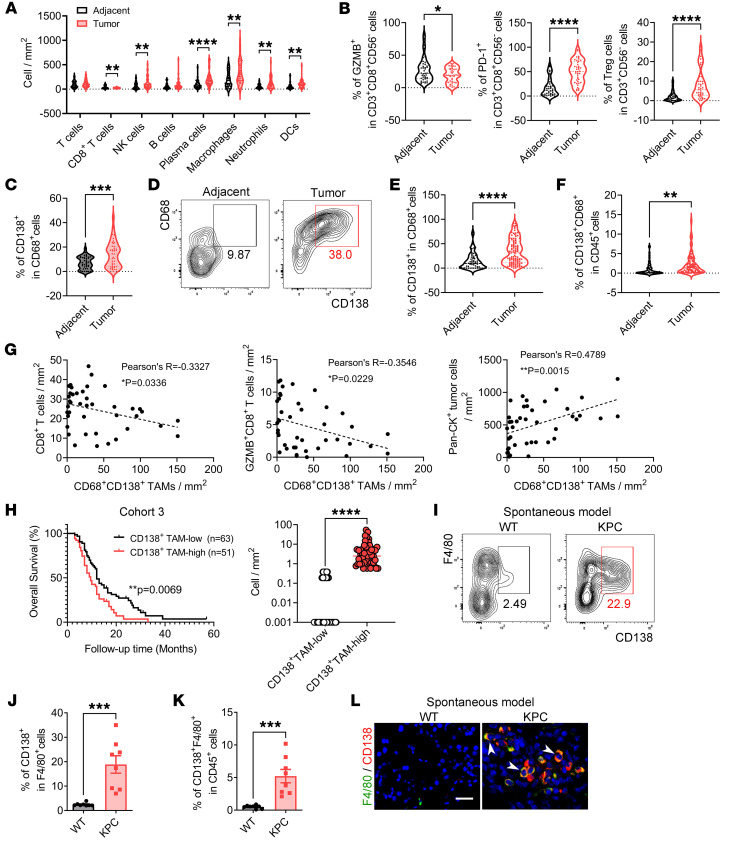
A CD138^+^ TAM subpopulation is identified and associated with immune evasion and poor prognosis in PDAC. (**A**–**C**) Abundance of various immune cell types (**A**) and the proportions of activated cytotoxic and exhausted CD8^+^ T cells within CD8^+^ T cells, Treg cells among T cells (**B**), and CD138^+^ TAMs among macrophages (**C**). The analysis utilized mIHC data from 41 paired adjacent benign and tumor tissue samples collected from patients with PDAC in cohort 2. (**D**) Representative images of CD138^+^ macrophages in adjacent benign (*n* = 58) and tumor tissues (*n* = 103) from patients with PDAC in cohort 1. (**E** and **F**) Quantification of data from **D**, with graphs depicting the frequencies of CD138^+^ macrophages in CD68^+^ macrophages (**E**) and in CD45^+^ cells (**F**). (**G**) Correlation among the abundance of CD138^+^ TAMs, CD8^+^ T cells, activated cytotoxic CD8^+^ T cells, and Pan-CK^+^ tumor cells in tumor tissues from patients with PDAC in cohort 2 (*n* = 41). (**H**) Kaplan-Meier survival curves generated for the abundance of CD138^+^ TAMs calculated through multispectral analysis of tumor tissues from patients with PDAC in cohort 3. This analysis categorized patients into 2 groups: CD138^+^ TAM-high and CD138^+^ TAM-low (graph on the right), revealing median survival times of 9.5 and 12 months, respectively (*P* value = 0.0069, HR 1.685, 95% CI 1.098–2.584). (**I**) Flow cytometry images of CD138^+^ macrophages in the tumor tissues of spontaneous KPC mice. (**J** and **K**) Quantification of data from **I**, representing the frequencies of CD138^+^ macrophages in F4/80^+^ macrophages (**J**) and in CD45^+^ cells (**K**) (*n* = 8 per group). (**L**) Immunofluorescence microscopy images of tumors from spontaneous KPC mice showing the presence of CD138^+^ macrophages (white arrowheads). Scale bar: 10 μm. *P < 0.05, **P < 0.01, ***P < 0.001, and ****P < 0.0001 by paired or unpaired 2-tailed Student’s *t* test (**A**–**C**, **E**, **F**, **J**, and **K**), by Pearson’s correlation analysis (**G**), and by log-rank analysis (**H**). Data represent mean ± SEM.

To explore the impact of tumor-infiltrating macrophages on immune evasion in PDAC, we assessed the cellular composition of the TME using multiplex immunohistochemistry (mIHC) staining on a tissue array comprising 41 paired adjacent benign and tumor tissues from patients with PDAC (referred to as cohort 2) (Supplemental Figure 1, A and B; supplemental material available online with this article; https://doi.org/10.1172/JCI199516DS1). The results revealed a significant decrease in the abundance of CD8^+^ T cells, accompanied by an increase in the density of NK cells, plasma cells, macrophages, neutrophils, and DCs in tumor tissues compared with adjacent benign tissues (Figure 1A). Furthermore, the analysis of T cell status within the PDAC TME indicated that the proportions of activated CD8^+^ cytotoxic T cells were significantly reduced, while the frequencies of exhausted CD8^+^ T cells and Treg cells were markedly elevated (Figure 1B). These findings suggest the presence of an immunosuppressive TME in patients with PDAC.

Notably, we identified a subset of TAMs expressing CD138, which was found to be expanded in the tumor tissues of PDAC patients in cohort 2 (Figure 1C and Supplemental Figure 1B). The emergence of this subset was further validated in fresh samples from patients with PDAC, including adjacent benign tissues (*n* = 58) and tumor tissues (*n* = 103), collectively referred to as cohort 1, utilizing flow cytometry (Figure 1, D–F). Thus, we investigated the roles of CD138^+^ TAMs in tumor immune escape. The findings demonstrated a negative correlation between the abundance of CD138^+^ TAMs and the presence of both CD8^+^ T cells and activated cytotoxic CD8^+^ T cells, while exhibiting a positive association with tumor cell density (Figure 1G). However, this abundance showed no significant relationship with the density of Treg cells or exhausted CD8^+^ T cells (Supplemental Figure 1C). These findings suggest that CD138^+^ TAMs contribute to tumor immune evasion by promoting the exclusion and dysfunction of CD8^+^ T cells. To further explore the impact of CD138^+^ TAMs on PDAC progression, we assessed their abundance in a tissue array comprising tumor samples from 114 patients with available prognostic information (designated as cohort 3) using mIHC staining. Patients were categorized into 2 subgroups: CD138^+^ TAM-low (*n* = 63) and CD138^+^ TAM-high (*n* = 51), based on the density of CD138^+^ TAMs (Figure 1H and Supplemental Figure 1D). Kaplan-Meier survival analysis demonstrated that patients in the CD138^+^ TAM-high group experienced significantly shorter overall survival times compared with those in the CD138^+^ TAM-low group (*P* value = 0.0069; Figure 1H). Additionally, univariate analysis revealed a negative correlation between CD138^+^ TAM abundance and tumor differentiation (Supplemental Table 1), indicating that patients with elevated levels of CD138^+^ TAMs are more likely to exhibit aggressive tumor behavior. Therefore, CD138^+^ TAMs may serve as potential indicators for PDAC progression.

To confirm the presence of CD138^+^ TAMs in PDAC mouse models, we first established a spontaneous PDAC mouse model using *Kras^G12D/+^ Trp53^R172H/+^ Pdx1^cre/+^* (hereafter referred to as KPC) mice (21). Tumor samples were collected at the stage when the disease burden in KPC mice became evident. As expected, a significant number of CD138^+^ TAMs were identified in the tumor tissues of KPC mice (Figure 1, I–L). These observations were further corroborated in the orthotopic KPC mouse model (Supplemental Figure 1, E–H) and in another spontaneous PDAC mouse model, *Kras^LSL-G12D^ TgfβR2^fl/fl^ Ptf1a^cre/+^* (hereafter referred to as KTC) mice (22) (Supplemental Figure 1, I–L). Collectively, these findings indicate that CD138^+^ TAMs are expanded in the PDAC TME and may contribute to tumor progression by promoting immune evasion.

### CD138^+^ TAMs exhibit robust proinflammatory and neutrophil-chemotactic activity.

Previous studies have documented the emergence of an antiinflammatory population of CD138^+^ macrophages during the resolution of inflammation in lupus (23) and infectious diseases (24) as well as the progression of abdominal aortic aneurysm (25). These cells are characterized by the elevated expression levels of IL-10R, CD206, and CCR2 as well as the activation of the cAMP/PKA/CREB pathway. This prompted us to investigate whether CD138^+^ TAMs within the PDAC TME represent such antiinflammatory populations. We conducted a transcriptional comparison between CD138^+^ and CD138^–^ TAMs in orthotopic tumors using SMART-seq. Contrary to our expectations, the transcriptional profile of CD138^+^ TAMs did not exhibit upregulation of *Il10ra*, *Il10rb*, *Mrc1*, or *Ccr2* nor did it demonstrate enrichment in the cAMP signaling pathway (Supplemental Figure 2, A and B and Supplemental Table 5). This may be attributed to the high expression of the cAMP-hydrolyzing enzyme phosphodiesterase 4D (*Pde4d*) (26) in these cells (Supplemental Figure 3F). Flow cytometry results confirmed the low expression levels of IL-10R and CD206 in CD138^+^ TAMs from patients with PDAC (Supplemental Figure 2C). These findings suggest that CD138^+^ TAMs may constitute a subset of macrophages separated from the previously identified antiinflammatory population.

**Figure 2:**
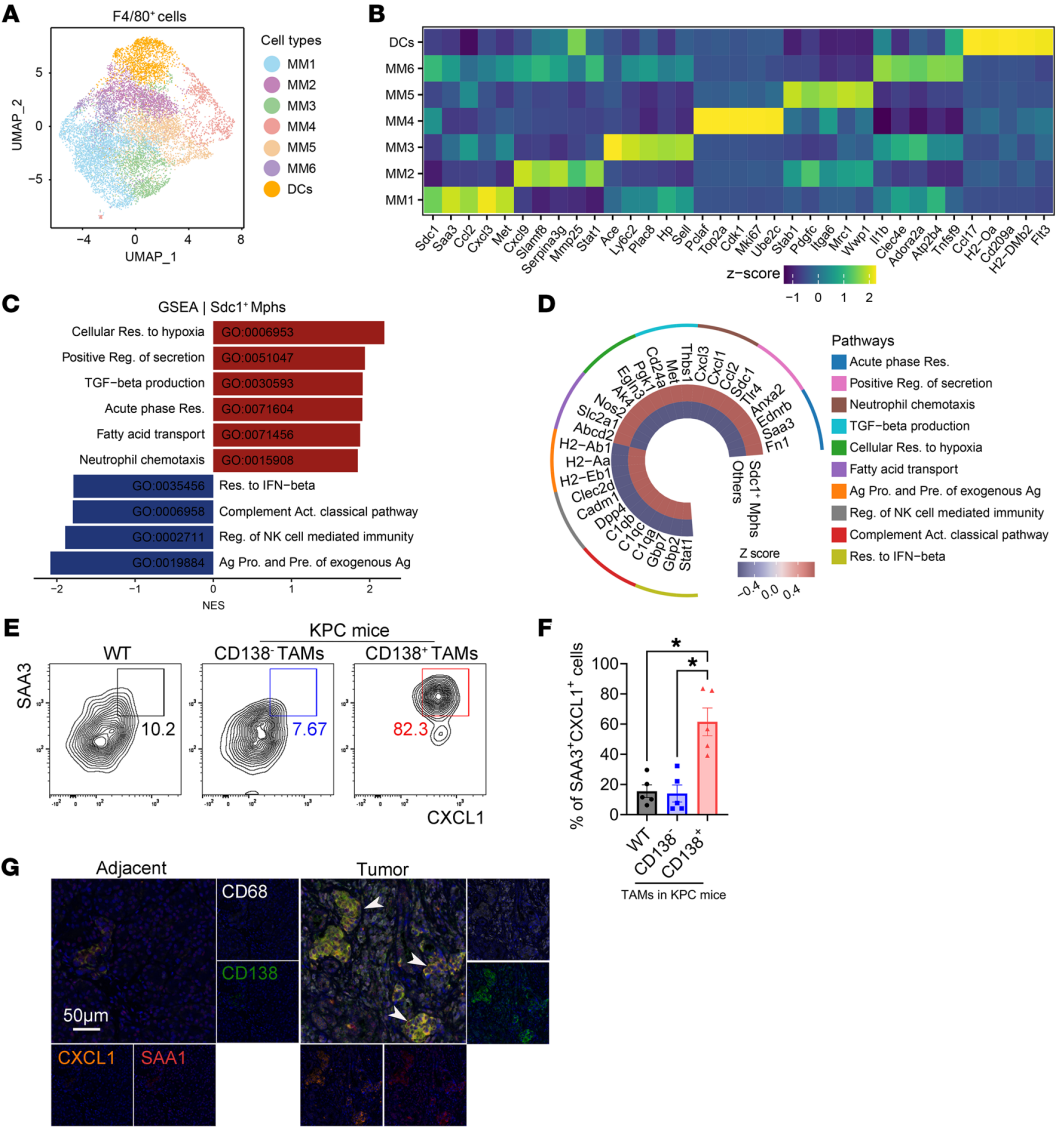
The CD138^+^ TAM subset exhibits proinflammatory and neutrophil-chemotactic activity. (**A**) UMAP plots illustrating F4/80^+^ cells sorted from orthotopic tumors, with colors representing distinct scRNA-seq clusters. (**B**) Heatmap displaying the scaled expression levels of marker genes across the identified macrophage and DC subsets. (**C**) GSEA of Gene Ontology biological processes on genes ranked by log_2_FC between *Sdc1*^+^ TAMs and other clusters. Mphs, macrophages; Res., response; Reg., regulation; Ag, antigen; Pro., processing; Pre., presentation; Act., activation; NES, normalized enrichment score. (**D**) Heatmap illustrating the expression of selected genes belonging to the categories in **C**, comparing *Sdc1*^+^ TAMs with other subsets depicted in **A**. (**E**) Frequencies of SAA3^+^CXCL1^+^ cells in F4/80^+^ macrophages derived from the pancreas of WT mice, as well as in F4/80^+^CD138^–^ and F4/80^+^CD138^+^ TAMs from orthotopic KPC mice. (**F**) Quantification of data from **E** (*n* = 5 per group). (**G**) Representative mIHC images depicting CD68^+^CD138^+^SAA1^+^CXCL1^+^ TAMs (white arrowheads) in paired adjacent benign and tumor tissues from patients with PDAC in cohort 2. Scale bar: 50 μm. *P < 0.05 by Kruskal-Wallis test with Dunn’s multiple-comparison test (**F**). Data represent mean ± SEM.

To further investigate the phenotype of CD138^+^ TAMs within the PDAC TME, scRNA-seq was performed on F4/80^+^ cells sorted from tumor tissues of the orthotopic KPC model. Following quality control, we extracted and analyzed 11,711 cells from ten mice. Genes were identified and embedded in a nondiscriminative manner, with dimensionality reduction executed using UMAP. Seven clusters were identified and categorized into 6 macrophage subclusters, along with 1 DC subcluster, which was distinguished by the upregulation of the classical DC marker *Flt3* (Figure 2, A and B and Supplemental Table 2). Among the macrophage subclusters, MM2 exhibited elevated expression of *Cxcl9* and *Stat1*, indicative of an M1 (hot) phenotype (27). MM6 was characterized by high expression levels of proinflammatory genes (*Il1b* and *Clec4e*) and aligned with inflammatory and noncytotoxic IL-1β^+^ macrophages (28). Additionally, a subcluster (MM3) was identified as a transitional population between monocytes and macrophages, retaining monocyte markers such as *Plac8* and *Ly6c2* (29). Other TAM clusters expressed genes linked to cell cycle regulation (*Ube2c*, *Top2a*, and *Mki67* in MM4) or an M2-like phenotype (*Mrc1*, *Pdgfc*, and *Wwp1* in MM5) (30, 31). Our analysis revealed a subcluster (MM1) with high expression levels of *Sdc1*, the gene encoding CD138 (Figure 2, A and B). A publicly available scRNA-seq dataset from the pancreas of healthy mice and those with acute pancreatitis (32) was integrated with our data to compare the proportions of macrophage subsets (Supplemental Figure 3, A–C and Supplemental Table 3). We observed that the frequency of *Sdc1*^+^ macrophages exhibited the most marked increase among all macrophage subsets within tumor tissues (Supplemental Figure 3D). Furthermore, gene signature scores for these macrophage subsets were computed from a bulk RNA-seq dataset of patients with PDAC sourced from The Cancer Genome Atlas (TCGA), utilizing the marker genes identified in the scRNA-seq data. Patients were categorized into 2 groups based on their gene signature score levels. Survival analysis indicated that patients in the MM1 score-high group experienced significantly shorter overall survival times (Supplemental Figure 3E). These findings suggest that *Sdc1*^+^ macrophages may play a critical role in promoting the progression of pancreatic cancer. Consequently, we conducted GSEA for *Sdc1*^+^ macrophages. The results indicated that the transcriptome of this population was enriched in neutrophil chemotaxis (*Cxcl1*, *Ccl2*, and *Cxcl3*), positive regulation of secretion (*Sdc1*, *Tlr4*, and *Anxa2*), acute phase response (*Saa3*, *Fn1*, and *Ednrb*), and cellular response to hypoxia (*Egln3*, *Ak4*, and *Pgk1*), while being depleted of pathways related to the regulation of NK cell–mediated immunity, antigen presentation, and complement activation, as determined by Gene Ontology terms (Figure 2, C and D and Supplemental Table 4). SMART-seq data revealed a comparable gene signature in CD138^+^ macrophages isolated from orthotopic tumors, characterized by the upregulation of proinflammatory and neutrophil-chemotactic factors, including *Saa3* and *Cxcl1* (Supplemental Figure 3F and Supplemental Table 6). Flow cytometry and mIHC analysis confirmed a marked elevation of SAA3 (SAA1, the ortholog of murine SAA3; ref. 33) and CXCL1 in CD138^+^ TAMs derived from tumor tissues of PDAC patients and orthotopic mouse models (Figure 2, E–G). These findings underscore CD138^+^ TAMs as a unique subset of macrophages characterized by the expression of proinflammatory and neutrophil-chemotactic programs within the PDAC TME.

### IL-34 and PGE_2_ elicit CD138^+^ TAMs.

**Figure 3:**
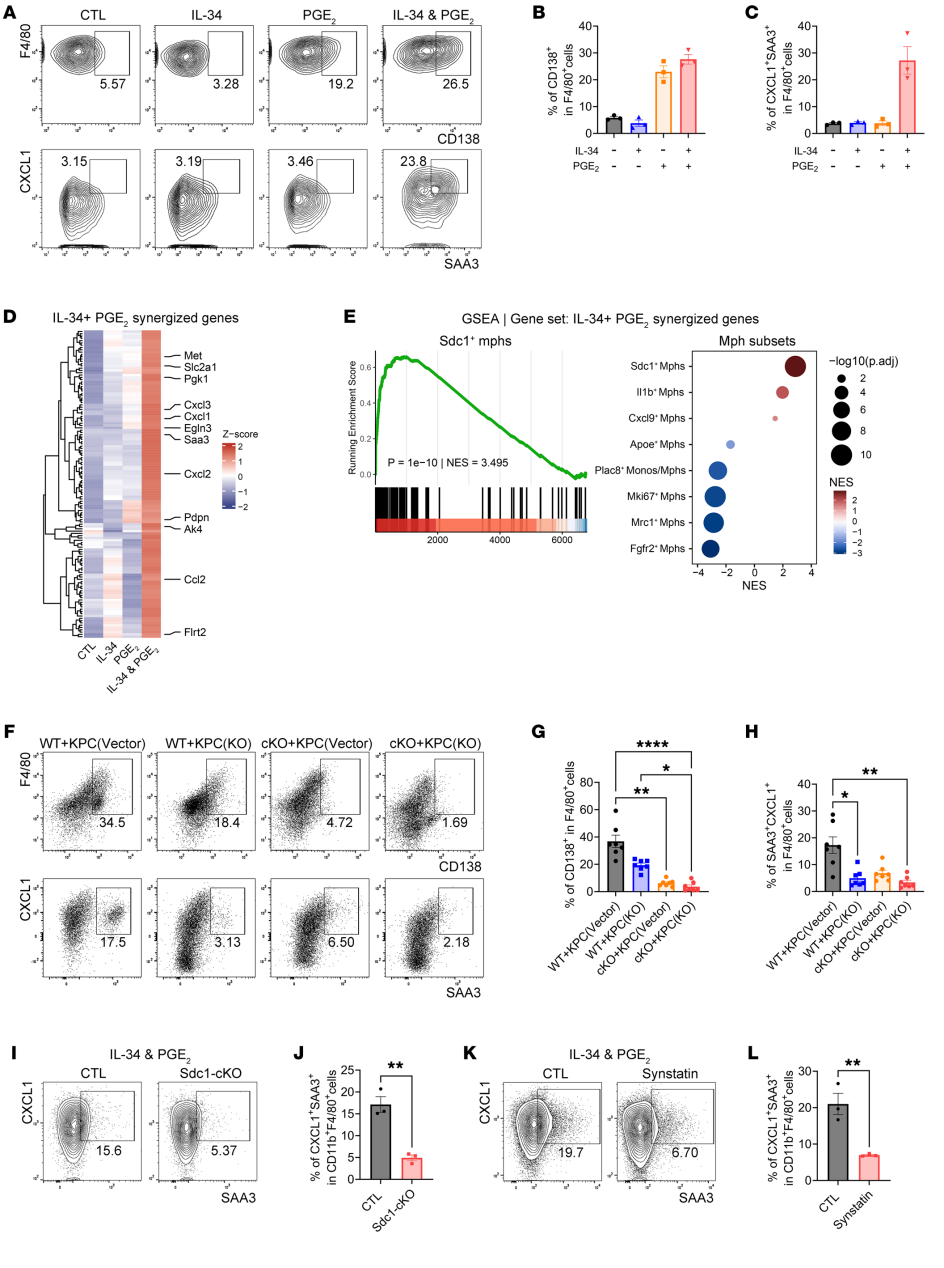
The formation of CD138^+^ TAMs is induced via IL-34/syndecan-1 and PGE_2_/EP2 signaling. (**A**) Representative flow cytometry images illustrating the expression levels of CD138, CXCL1, and SAA3 in F4/80^+^ cells from BMDM cultures treated with IL-34 and/or PGE_2_. (**B** and **C**) Quantification of data from **A**, showcasing the percentages of CD138^+^ (**B**) and CXCL1^+^SAA3^+^ (**C**) within the F4/80^+^ population (*n* = 3 per group). (**D**) Heatmap of the relative expression of genes synergized by IL-34 plus PGE_2_ in BMDM cultures. (**E**) GSEA of genes synergized by IL-34 plus PGE_2_, on genes ranked by their correlation with the fate probability of the monocyte-to-*Sdc1*^+^ TAM trajectory (left) and by log_2_FC between each monocyte/macrophage (Mono/Mph) subset versus other monocytes/macrophages (right). (**F**) Representative images showing the expression of CD138, CXCL1, and SAA3 in F4/80^+^ cells within tumor tissues from control or Ptger2-cKO mice bearing orthotopic KPC^vector^ or KPC^Il34-KO^ tumors. (**G** and **H**) Quantification of data from **F**, illustrating the percentages of CD138^+^ (**G**) and CXCL1^+^SAA3^+^ (**H**) within F4/80^+^ macrophages (*n* = 7 per group). (**I**) Flow cytometry images of CXCL1^+^SAA3^+^ macrophages in the cultures of BMDMs derived from control or Sdc1-cKO mice in the presence of IL-34 and PGE_2_. (**J**) Quantification of data from **I**, showing the percentages of CXCL1^+^SAA3^+^ within F4/80^+^ macrophages (*n* = 3 per group). (**K**) Representative images of CXCL1^+^SAA3^+^ macrophages in BMDM cultures treated with IL-34 and PGE_2_, with or without synstatin, a selective inhibitor of syndecan-1. (**L**) Quantification of data from **K**, highlighting the percentages of CXCL1^+^SAA3^+^ in F4/80^+^ macrophages (*n* = 3 per group). *P < 0.05, **P < 0.01, and ****P < 0.0001 by Kruskal-Wallis test with Dunn’s multiple-comparison test (**G** and **H**) and by unpaired 2-tailed Student’s *t* test (**J** and **L**). Data represent mean ± SEM.

To explore the mechanisms responsible for the generation of CD138^+^ TAMs, we established a CD45.1/CD45.2 chimera model with orthotopic KPC tumors (Supplemental Figure 4A). Flow cytometry analysis revealed that CD138^+^ TAMs in the chimeric mice predominantly expressed CD45.1 (Supplemental Figure 4, B and C), indicating that these cells originate from circulating monocytes rather than from tissue-resident macrophages. Given that syndecan-1 serves as a functional receptor for IL-34 (34), we investigated the potential roles of IL-34 in eliciting the CD138^+^ TAM state. To determine the expression of IL-34 in PDAC, scRNA-seq was conducted using tumor tissues isolated from orthotopic KPC mice 21 days after implantation. The data were then integrated with a publicly available scRNA-seq dataset from blood samples of 3 orthotopic KPC mice (6). Following quality control, we extracted and reanalyzed 22,061 cells. Genes were identified and embedded in a nondiscriminative manner, with dimensionality reduction performed using UMAP. Eight clusters were identified and categorized into 2 nonimmune cell types, namely, ductal cells and cancer-associated fibroblasts, as well as 6 immune cell types: monocytes/macrophages, neutrophils, DCs (including cDC1 and cDC2), B cells, T/NK cells, and mast cells (Supplemental Figure 5A). Signature genes for each cluster were cross-referenced with established markers from the literature to accurately identify the different cell types represented by the clusters (Supplemental Figure 5B and Supplemental Table 7). The results revealed that IL-34 was predominantly derived from ductal cells (Supplemental Figure 5C). mIHC analysis confirmed a substantial increase in IL-34 levels in tumor cells from both orthotopic KPC mice and patients with PDAC (Supplemental Figure 5, D–G). However, treatment of mouse bone marrow–derived macrophages (BMDMs) with IL-34 did not induce syndecan-1 expression or the synthesis of SAA3 and CXCL1, suggesting the requirement of additional factors (Figure 3, A–C). Prostaglandin E_2_ (PGE_2_), a lipid inflammatory mediator, was found to be elevated in PDAC (35–37), stimulating syndecan-1 expression in BMDMs (24). Furthermore, our findings indicated a notable rise in PGE_2_ levels in the serum of both orthotopic KPC mice and individuals with PDAC (Supplemental Figure 6, A and B). RT-PCR data revealed elevated expression levels of *Ptgs2* (COX-2, a critical enzyme in PGE_2_ synthesis; ref. 38) in neutrophils isolated from orthotopic KPC tumors 10 days after implantation (Supplemental Figure 6C), suggesting that PGE_2_ is primarily derived from tumor-infiltrating neutrophils prior to the emergence of CD138^+^ TAMs (Supplemental Figure 15). Thus, we investigated whether PGE_2_ contributed to the production of CD138^+^ TAMs. Flow cytometry analysis demonstrated that PGE_2_ alone upregulated the expression of syndecan-1 in a dose-dependent manner, while exhibiting limited effects on the synthesis of SAA3 and CXCL1 in BMDMs (Figure 3, A–C, and Supplemental Figure 6, D and E). However, its cotreatment with IL-34 resulted in a robust production of SAA3 and CXCL1 in BMDMs (Figure 3, A–C). Bulk RNA-seq revealed a distinct set of transcripts that were synergistically induced by IL-34 plus PGE_2_ (Figure 3D and Supplemental Table 8). These transcripts were notably enriched in the transcriptome of *Sdc1*^+^ TAMs and among the driver genes of the monocyte-to-*Sdc1*^+^ TAM transition, as evidenced by scRNA-seq data, which included reclustering of the monocyte/macrophage subset (Figure 3E, Supplemental Figure 7, A–D, and Supplemental Tables 9 and 10). These genes encoded for proteins that trigger acute inflammation (*Saa3*) and enhance neutrophil recruitment (*Cxcl1*, *Ccl2*, and *Cxcl3*) (Figure 3D). Furthermore, the morphology, migration, and phagocytosis of CD138^+^ macrophages induced by the combination of IL-34 and PGE_2_, along with CD138^+^ TAMs isolated from orthotopic tumors, were analyzed. We found that both of them showed enlarged size, irregular shape, extended pseudopods, and enhanced migration and phagocytic capabilities compared with the control group (Supplemental Figure 8). Together, these findings suggest that IL-34 and PGE_2_ induce the production of CD138^+^ TAMs.

To further confirm this hypothesis, we examined the effects of IL-34 and PGE_2_ on the differentiation of CD138^+^ TAMs in vivo. RT-PCR analysis revealed a dramatic increase in the transcriptional levels of EP2 (encoded by *Ptger2*) rather than other PGE_2_ receptors (39) in both CD138^+^ TAMs isolated from orthotopic tumors and BMDMs exposed to PGE_2_ (Supplemental Figure 7, E and F). Furthermore, the introduction of PGE_2_ receptor inhibitors into the BMDM cultures demonstrated that the EP2 antagonist (PF-04418948) notably inhibited the effect of PGE_2_ on the upregulation of syndecan-1 expression (Supplemental Figure 7, G and H). Thus, we established an orthotopic model comprising *Ptger2^fl/fl^* *Csf1r-Cre* (hereafter referred to as Ptger2-cKO) mice and a KPC cell line with silenced IL-34 expression, which was subsequently analyzed using flow cytometry (Supplemental Figure 7, I–K). The results indicated that either silencing IL-34 expression or conditionally knocking out *Ptger2* in monocytes/macrophages effectively impeded the generation of CD138^+^ TAMs in the orthotopic tumors (Figure 3, F–H). To determine whether IL-34 relies on syndecan-1 for mediating CD138^+^ TAM differentiation, BMDMs derived from *Sdc1^fl/fl^* *Csf1r-Cre* (hereafter referred to as Sdc1-cKO) mice, which allow for the conditional knockout of *Sdc1* in monocytes/macrophages, were exposed to IL-34 and PGE_2_. Flow cytometry analysis showed that knockout of *Sdc1* in BMDMs significantly suppressed their synthesis of SAA3 and CXCL1 (Figure 3, I and J). The introduction of synstatin, a selective inhibitor of syndecan-1 (40), into the BMDM culture system yielded similar results (Figure 3, K and L). Collectively, these phenomena demonstrate that CD138^+^ TAMs are induced by IL-34/syndecan-1 and PGE_2_/EP2 signaling.

Next, we investigated the molecular mechanisms through which IL-34 and PGE_2_ drive the differentiation of CD138^+^ TAMs. Notably, we observed an upregulated gene set enriched in the PI3K-Akt, NF-κB, and Rap1 signaling pathways in *Sdc1*^+^ TAMs (Supplemental Figure 9A and Supplemental Table11). These pathways are well-established downstream signaling routes for CSF1R (a classical IL-34 receptor) and PGE_2_ receptors (41, 42). Upon introducing antagonists or inhibitors targeting these pathways into the culture systems, we found that inhibitors of the PI3K/Akt (PI3K/AKT-IN-1) and NF-κB (JSH-23) pathways suppressed the synthesis of SAA3 and CXCL1, while leaving CD138 expression unaffected in BMDMs cultured with IL-34 and PGE_2_ (Supplemental Figure 9, B–D). In contrast, the EPAC/Rap1 pathway antagonist ESI-08 significantly inhibited the expression of CD138 as well as the production of SAA3 and CXCL1 (Supplemental Figure 9, B–D). These findings indicate that IL-34 activates the PI3K/Akt/NF-κB pathway, while PGE_2_ primarily stimulates the EPAC/Rap1 pathway, collectively driving the differentiation of CD138^+^ TAMs. Together, these findings demonstrate that IL-34 and PGE_2_ collaboratively induce the generation of CD138^+^ TAMs within the PDAC TME and enhance the synthesis of proinflammatory factors such as SAA3 and CXCL1 in these cells.

### CD138^+^ TAMs promote the progression of PDAC by facilitating tumor immune escape.

**Figure 4:**
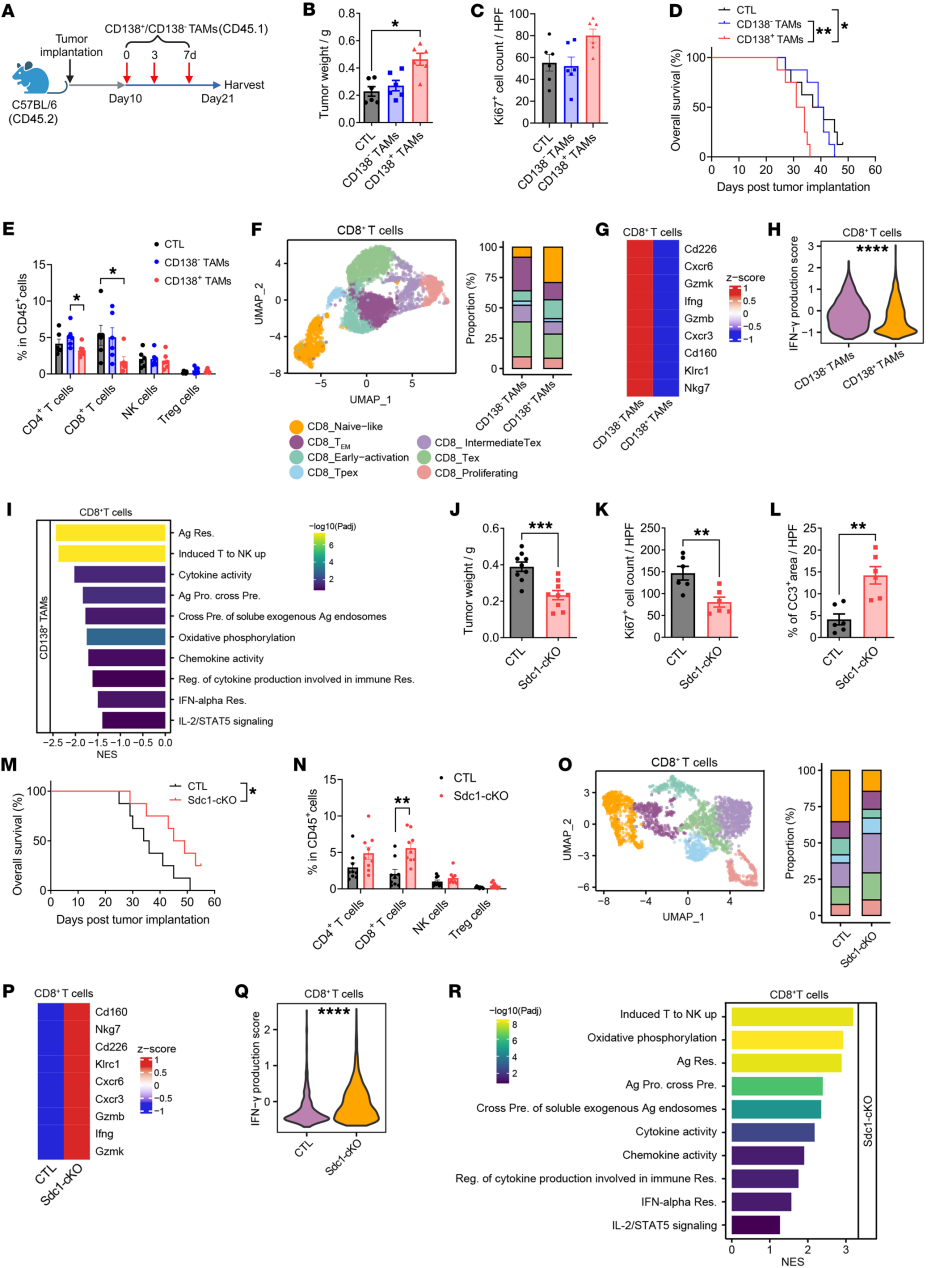
CD138^+^ TAMs facilitate the progression of PDAC by promoting tumor immune evasion. (**A**) Experimental approach utilized for the adoptive transfer of CD138^+^ or CD138^–^ TAMs isolated from CD45.1^+^ orthotopic tumors into CD45.2^+^ orthotopic KPC mice. (**B** and **C**) Quantification of tumor weight in mice (**B**) and the number of Ki67^+^ cells per ×40 field in tumor sections (**C**) (*n* = 6 per group). HPF, high power field. (**D**) Overall survival probabilities of mice that received the adoptive transfer (*n* = 8 per group). (**E**) Quantification of the frequencies of effector cell subsets within CD45^+^ cells in tumor tissues (*n* = 6 per group). (**F**) Analysis of UMAP (left) and the proportions (right) of CD8^+^ T cell clusters in tumor tissues. (**G**) Heatmap of the relative expression of effector cytokines in tumor-infiltrating CD8^+^ T cells. (**H**) GSEA of the gene set related to IFN-γ production. (**I**) GSEA of pathways associated with CD8^+^ T cell proliferation and activation. (**J**) Quantification of tumor weight in mice (*n* = 9 per group). (**K** and **L**) Quantification of the number of Ki67^+^ cells (**K**) and the percentage of CC3^+^ area (**L**) per ×40 field in tumor sections (*n* = 6 per group). CC3, cleaved caspase-3. (**M**) Overall survival probabilities of control and Sdc1-cKO mice (*n* = 8 per group). (**N**) Quantification of the frequencies of effector cell subsets within CD45^+^ cells in tumor tissues (*n* = 9 per group). (**O**) Analysis of UMAP (left) and the proportions (right) of CD8^+^ T cell clusters in tumor tissues. (**P**) Heatmap of the relative expression of effector cytokines in tumor-infiltrating CD8^+^ T cells. (**Q**) GSEA of the gene set related to IFN-γ production. (**R**) GSEA of pathways associated with CD8^+^ T cell proliferation and activation. *P < 0.05, **P < 0.01, and ***P < 0.001 by Kruskal-Wallis test with Dunn’s multiple-comparison test (**B** and **E**), by log-rank analysis (**D** and **M**), and by unpaired 2-tailed Student’s *t* test (**J**–**L** and **N**). Data represent mean ± SEM.

To investigate the roles of CD138^+^ TAMs in the progression of PDAC, an adoptive transfer assay was conducted using the orthotopic KPC model. Briefly, CD138^+^ TAMs extracted from orthotopic tumors were intravenously injected into orthotopic KPC mice every 3 to 4 days for a total of 3 injections, starting at 10 days after tumor implantation (Figure 4A). The infiltration of CD138^+^ TAMs into the TME was confirmed through flow cytometry (Supplemental Figure 10A). The results indicated that mice receiving the adoptive transfer of CD138^+^ TAMs exhibited increased tumor weight and enhanced cell proliferation in tumor tissues, suggesting a more aggressive cancer phenotype compared with the control groups (Figure 4, B and C, and Supplemental Figure 10, B and C). Notably, survival analysis revealed significantly shorter overall survival times in the group injected with CD138^+^ TAMs (Figure 4D). Flow cytometry analysis revealed a marked reduction in the infiltration of CD8^+^ T cells in mice following the adoptive transfer of CD138^+^ TAMs (Figure 4E). scRNA-seq data indicated an increased proportion of CD8^+^ T cells exhibiting a naive phenotype, accompanied by a decrease in the fractions of effector memory, precursor exhausted, terminally exhausted (Tex), intermediate exhausted (intermediate Tex), and proliferating subsets within tumors that received the adoptive transfer of CD138^+^ TAMs (Figure 4F and Supplemental Figure 10D and Supplemental Table 12). Furthermore, CD8^+^ T cells in tumors injected with CD138^+^ TAMs showed diminished activity and cytotoxicity, as evidenced by the reduced expression of effector factors (*Ifng*, *Gzmb*, and *Nkg7*), a lower IFN-γ production score, and downregulation of pathways related to CD8^+^ T cell proliferation and activation (Figure 4, G–I and Supplemental Table 12). These findings emphasize the potential roles of CD138^+^ TAMs in accelerating PDAC progression by promoting tumor immune evasion. We further validated the protumorigenic roles of CD138^+^ TAMs using an orthotopic mouse model developed with Sdc1-cKO mice, in which CD138^+^ TAMs were specifically depleted (Supplemental Figure 10E). Tumor weight was significantly reduced in Sdc1-cKO mice with orthotopic KPC tumors compared with control mice (Figure 4J and Supplemental Figure 10F). Furthermore, histopathological analysis revealed that the depletion of CD138^+^ TAMs led to decreased cell proliferation and increased apoptosis in the tumor tissues (Figure 4, K and L, and Supplemental Figure 10G). Importantly, Sdc1-cKO mice bearing orthotopic KPC tumors exhibited a dramatic increase in overall survival times compared with those in the control group (Figure 4M). To evaluate the immune status of the TME, we assessed tumor-infiltrating lymphocytes through flow cytometry and scRNA-seq. The results demonstrated a significant increase in the infiltration of CD8^+^ T cells (Figure 4N), as well as in the fractions of effector memory, precursor exhausted, intermediate Tex, Tex, and proliferating subsets among CD8^+^ T cells within the tumor tissues of mice in the Sdc1-cKO group (Figure 4O, Supplemental Figure 10H, and Supplemental Table 13). Furthermore, tumor-infiltrating CD8^+^ T cells in the Sdc1-cKO group displayed signs of activation, as supported by increased expression of effector factors, a higher IFN-γ production score, and upregulation of pathways associated with T cell proliferation and activation (Figure 4, P–R and Supplemental Table 13). These results suggest that the depletion of CD138^+^ TAMs can effectively hinder the development of PDAC by activating the antitumor immunity of CD8^+^ cytotoxic T cells. Additionally, we investigated the tumor-promoting roles of CD138^+^ TAMs using a chimera mouse model established by spontaneous KPC and Sdc1-cKO mice (Supplemental Figure 10I). The results revealed a notable reduction in tumor incidence rates among the KPC/Sdc1-cKO chimera mice (Supplemental Figure 10J). Collectively, these findings underscore the substantial role of CD138^+^ TAMs in driving the advancement of PDAC by promoting tumor immune evasion.

### A feed-forward loop involving CD138^+^ TAMs and Siglec-F^+^ neutrophils drives immune escape in PDAC.

**Figure 5:**
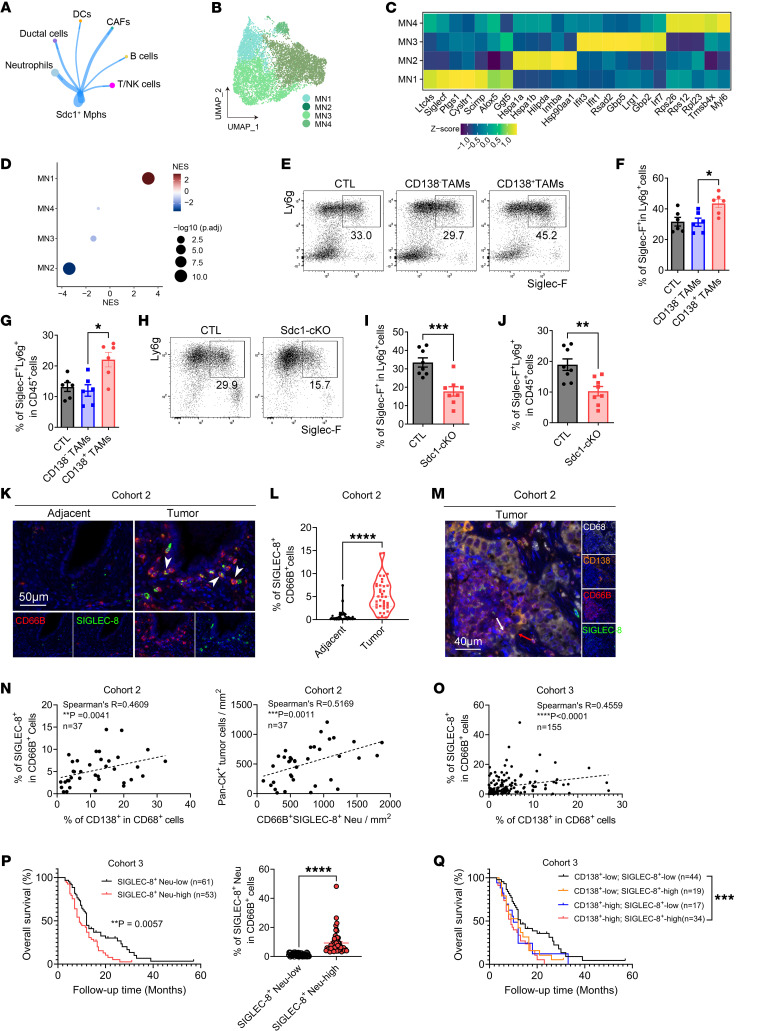
There is a significant cell-to-cell interaction between CD138^+^ TAMs and Siglec-F^+^ neutrophils in PDAC. (**A**) Cell-to-cell interactions among *Sdc1*^+^ TAMs and other major clusters, visualized using CellChat. (**B**) UMAP plots illustrating the reclustering of neutrophils. (**C**) Heatmap depicting the scaled expression of marker genes across neutrophil subsets. (**D**) GSEA of the gene set comprising DEGs between Siglec-F^+^ and Siglec-F^–^ neutrophils in orthotopic tumors, based on the marker genes of neutrophil subsets. (**E**) Images depicting the presence of Siglec-F^+^ neutrophils in orthotopic tumors following the adoptive transfer of CD138^+^ TAMs. (**F** and **G**) Quantification of data from **E** (*n* = 6 per group). (**H**) Images displaying Siglec-F^+^ neutrophils in the orthotopic tumors of Sdc1-cKO mice. (**I** and **J**) Quantification of data from **H** (*n* = 8 per group). (**K**) Images showing SIGLEC-8^+^ neutrophils (white arrowheads) in patients with PDAC. Scale bar: 50 μm. (**L**) Quantification of data from **K** (*n* = 37–38 per group). (**M**) Images revealing the colocalization of CD138^+^ TAMs (red arrow) with SIGLEC-8^+^ neutrophils (white arrow) in patients with PDAC. Scale bar: 40 μm. (**N** and **O**) The correlation among SIGLEC-8^+^ neutrophils, CD138^+^ TAMs, and Pan-CK^+^ tumor cells in patients with PDAC. (**P**) Kaplan-Meier survival curves generated based on the frequencies of SIGLEC-8^+^ neutrophils. The curves reveal a median survival of 9.0 months for the SIGLEC-8^+^ Neu-high group compared with 12 months for the SIGLEC-8^+^ Neu-low group (*P* value = 0.0057, HR 1.860, 95% CI 1.198–2.888). (**Q**) Kaplan-Meier survival curves generated for CD138^+^ TAM abundance and SIGLEC-8^+^ neutrophil frequency. Comparison between CD138^+^-high, SIGLEC-8^+^-high and CD138^+^-low, and SIGLEC-8^+^-low groups yields a median survival of 9.0 versus 13.0 months (*P* value = 0.0010, HR 2.635, 95% CI 1.482–4.687). *P < 0.05, **P < 0.01, ***P < 0.001, and ****P < 0.0001 by Kruskal-Wallis test with Dunn’s multiple-comparison test (**F** and **G**), by paired or unpaired 2-tailed Student’s *t* test (**I**, **J**, and **L**), by Spearman’s correlation analysis (**N** and **O**), and by log-rank analysis (**P** and **Q**). Data represent mean ± SEM.

To explore the mechanisms by which CD138^+^ TAMs promote immune evasion in PDAC, we first established a coculture system using OVA-stimulated OT1 CD8^+^ T cells and TAMs isolated from mice bearing orthotopic KPC tumors. Flow cytometry analysis revealed no significant changes in IFN-γ levels in OT1 CD8^+^ T cells when cocultured with CD138^+^ TAMs (Supplemental Figure 11), suggesting that CD138^+^ TAMs do not directly influence CD8^+^ T cell function. Given the marked crosstalk between *Sdc1*^+^ TAMs and neutrophils revealed by CellChat (Figure 5A), the enrichment of signaling pathways related to neutrophil chemotaxis in the transcriptome of *Sdc1*^+^ TAMs (Figure 2, C and D), and the known crucial roles of neutrophils in tumor growth and immune evasion in PDAC (43), we addressed the possibility that CD138^+^ TAMs drive the progression of PDAC by modulating the properties of neutrophils. Subclustering of neutrophils based on scRNA-seq data (Supplemental Figure 5A) uncovered 4 distinct neutrophil subsets within the TME of orthotopic KPC mice (Figure 5B and Supplemental Table 14). MN1 was characterized by the expression of genes encoding cell surface proteins that bind to sialic acid (*Siglecf*) and enzymes essential for the synthesis of lipid mediators of inflammation (*Ltc4s*, *Ptgs1*, and *Cysltr1*), aligning with previously described populations (44); MN3 exhibited a unique set of interferon-stimulated genes, including *Ifit3*, *Ifit1*, and *Irf7*, corresponding to interferon-stimulated neutrophils (45). Other neutrophil clusters displayed high expression of ribosome-related genes (*Rps26*, *Rps12*, and *Rpl23* in MN4) and genes related to cell structure and motility (*Tmsb4x* in MN4) (46) or heat shock protein–associated genes (*Hspa1b*, *Hspa1a*, and *Hsp90aa1* in MN2) (Figure 5C). Subsequently, our data were integrated with the dataset from the pancreas of healthy mice and those with acute pancreatitis (32) to compare the fractions of neutrophil subsets (Supplemental Figure 3, A and B, and Supplemental Figure 12A). The results revealed a marked increase in the proportions of MN1 and MN2 within orthotopic KPC tumors (Supplemental Figure 12B). Notably, the gene signature score of MN1, but not MN2, exhibited a significant correlation with the poor prognosis of PDAC patients, as indicated by the dataset from TCGA (Supplemental Figure 12C). Flow cytometry analysis confirmed a higher frequency of Siglec-F^+^ neutrophils in the tumor tissues of orthotopic KPC mice compared with WT mice (Supplemental Figure 12, D–F). Thus, we conducted transcriptomic analysis of Siglec-F^+^ neutrophils isolated from the orthotopic tumors using bulk RNA-seq, followed by GSEA of the scRNA-seq subsets of neutrophils based on the gene signature of these cells. The results demonstrated significant comparability in transcriptional programs between sorted Siglec-F^+^ neutrophils and the MN1 subset (Figure 5D, Supplemental Figure 12G, and Supplemental Table 15), indicating that Siglec-F^+^ can be utilized to identify the MN1 subset. Siglec-F^+^Ly6g^+^ cells derived from orthotopic tumors were confirmed to be a subset of neutrophils, as evidenced by their multilobed nuclei and pale pink cytoplasm containing fine, light purple granules, which were visible through Wright-Giemsa staining. Additionally, these cells exhibited minimal expression of CCR3 (Supplemental Figure 12H). Notably, a significant increase in the proportion of Siglec-F^+^ neutrophils was observed in the tumor tissues of orthotopic KPC mice following the adoptive transfer of CD138^+^ TAMs (Figure 5, E–G). However, the depletion of CD138^+^ TAMs through genetic ablation of IL-34/syndecan-1 signaling using Sdc1-cKO mice resulted in a significant decrease in the proportion of the neutrophil subset within orthotopic tumors (Figure 5, H–J). Furthermore, a notable increase in the frequency of SIGLEC-8^+^ neutrophils, which correspond to the human equivalent of murine Siglec-F^+^ neutrophils (47), was observed in the primary tumors of patients with PDAC from cohort 2 (Figure 5, K and L). To investigate the correlation among CD138^+^ TAMs, SIGLEC-8^+^ neutrophils, and tumor cells in patients, we conducted a mIHC assay on tissue arrays derived from cohorts 2 and 3. Our findings indicated a positive correlation between the frequency of SIGLEC-8^+^ neutrophils and CD138^+^ TAMs, as well as between the abundance of SIGLEC-8^+^ neutrophils and tumor cells in the tumor tissues of these patients (Figure 5, M–O, and Supplemental Figure 1B). Furthermore, the analysis of localization patterns revealed that CD138^+^ TAMs exhibited significantly higher effective scores and percentages, 2 indicators that integrate both cell proximity and numbers to evaluate cell distribution (48), compared with CD138^–^ TAMs (Supplemental Figure 12, I–L). This suggests a colocalization and effective cell-to-cell interaction between CD138^+^ TAMs and SIGLEC-8^+^ neutrophils. Notably, the presence of SIGLEC-8^+^ neutrophils was correlated with negative outcomes in patients with PDAC (Figure 5P). Patients with elevated levels of both CD138^+^ TAMs and SIGLEC-8^+^ neutrophils had the poorest prognoses (Figure 5Q). Altogether, these findings imply a potential influence of CD138^+^ TAMs on the activity and accumulation of Siglec-F^+^ (SIGLEC-8^+^) neutrophils and the protumorigenic roles of this neutrophil subset.

**Figure 6:**
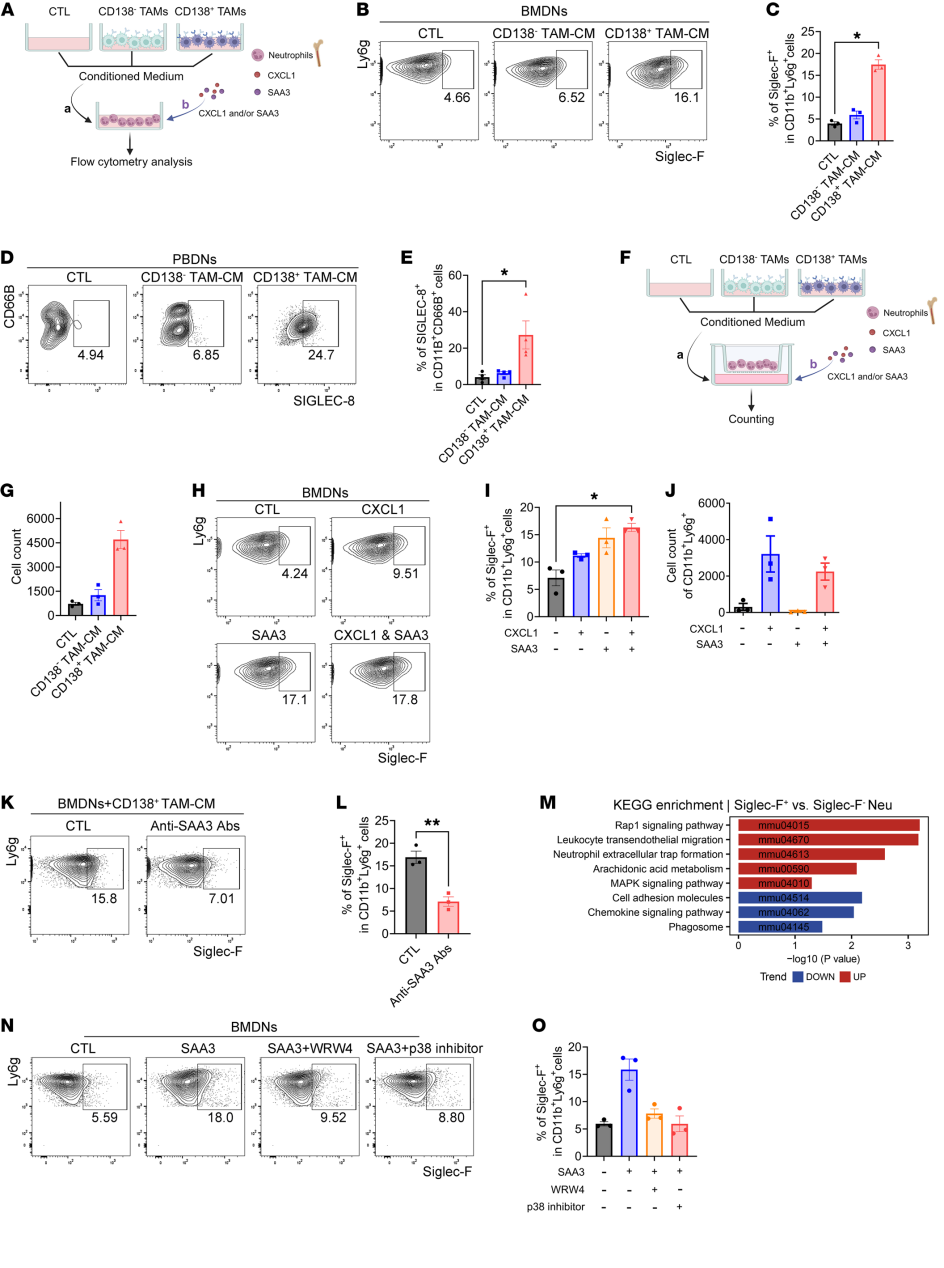
CD138^+^ TAMs promote the polarization and migration of Siglec-F^+^ neutrophils through the actions of SAA3 and CXCL1. (**A**) Schematic illustrating the culture of BMDNs treated with CM derived from CD138^+^ or CD138^–^ TAMs isolated from orthotopic tumors, as well as with SAA3 and/or CXCL1. (**B** and **H**) Representative images depicting the frequencies of Siglec-F^+^ in Ly6g^+^ neutrophils in BMDN cultures upon treatment with CM (**B**) or CXCL1 and/or SAA3 (**H**). (**C**) Quantification of data from **B** (*n* = 3 per group). (**D**) Images revealing the percentages of SIGLEC-8^+^ in CD66B^+^ neutrophils in human peripheral blood–derived neutrophil (PBDN) cultures exposed to CM derived from CD138^+^ or CD138^–^ TAMs in patients with PDAC. (**E**) Quantification of data from **D** (*n* = 4 per group). (**F**) Experimental approach to evaluate the cell migration capacity of BMDNs through a transwell culture system exposed to CM derived from CD138^+^ or CD138^–^ TAMs isolated from orthotopic tumors, as well as to SAA3 and/or CXCL1. (**G** and **J**) Quantification of the number of migrated cells in the transwell culture system exposed to CM (**G**) or CXCL1 and/or SAA3 (**J**) (*n* = 3 per group). (**I**) Quantification of data from **H** (*n* = 3 per group). (**K**) Representative images depicting the frequencies of Siglec-F^+^ in Ly6g^+^ neutrophils in BMDN cultures exposed to CD138^+^ TAM-CM with or without anti-SAA3 neutralizing antibodies. (**L**) Quantification of data from **K** (*n* = 3 per group). (**M**) KEGG enrichment analysis of DEGs between Siglec-F^+^ and Siglec-F^–^ neutrophils derived from orthotopic tumors. (**N**) Images displaying the frequencies of Siglec-F^+^ in Ly6g^+^ neutrophils in BMDN cultures exposed to SAA3 with or without WRW4 (a FPR2 antagonist) or p38 MAPK-IN-1 (a p38 MAPK pathway inhibitor). (**O**) Quantification of data from **N** (*n* = 3 per group). *P < 0.05 and **P < 0.01 by Kruskal-Wallis test with Dunn’s multiple-comparison test (**C**, **E**, and **I**) and by unpaired 2-tailed Student’s *t* test (**L**). Data represent mean ± SEM.

To investigate whether CD138^+^ TAMs directly influence the characteristics of Siglec-F^+^ neutrophils, neutrophils extracted from the bone marrow of WT mice were exposed to conditioned medium (CM) derived from either CD138^+^ or CD138^–^ TAMs (Figure 6A). The results revealed a significant increase in the proportions of Siglec-F^+^ neutrophils when exposed to CD138^+^ TAM-CM (Figure 6, B and C). A similar phenomenon was observed in cultures of human peripheral blood–derived neutrophils when exposed to CD138^+^ TAM-CM from patients with PDAC (Figure 6, D and E). In vitro chemotaxis analysis (Figure 6F) demonstrated that CD138^+^ TAM-CM markedly enhanced neutrophil trafficking compared with CD138^–^ TAM-CM (Figure 6G). Phenotypic analysis indicated that CD138^+^ TAMs exhibited elevated expression levels of factors that mediate neutrophil properties, such as SAA3 and CXCL1 (49) (Figure 2, B–G, and Supplemental Figure 3F). Further examination of the impacts of SAA3 and CXCL1 expressed by CD138^+^ TAMs on neutrophil characteristics, using BMDN culture systems (Figure 6, A and F), revealed that SAA3 promoted the polarization of Siglec-F^+^ neutrophils (Figure 6, H and I), while CXCL1 enhanced neutrophil migration (Figure 6J). This observation was corroborated by the blockade of BMDN polarization into Siglec-F^+^ neutrophils induced by CD138^+^ TAM-CM using anti-SAA3 neutralizing antibodies (Figure 6, K and L). To explore the mechanism by which SAA3 induces the polarization of Siglec-F^+^ neutrophils, we conducted KEGG enrichment analysis of differentially expressed genes (DEGs) in Siglec-F^+^ neutrophils utilizing bulk RNA-seq data. The results indicated that the upregulated genes in these cells were significantly enriched in MAPK signaling pathways (Figure 6M and Supplemental Table 15), a canonical downstream pathway of SAA3 (50–52). Upon introducing inhibitors targeting the p38 MAPK pathway and FPR2 (a primary receptor of SAA3; ref. 52) into the BMDN cultures, we found that both inhibitors markedly impeded the polarization of BMDNs into Siglec-F^+^ neutrophils (Figure 6, N and O). These phenomena suggest that SAA3 induces the polarization of Siglec-F^+^ neutrophils through binding to FPR2 and subsequently stimulating the p38 MAPK pathway. Collectively, these findings demonstrate that CD138^+^ TAMs play a crucial role in regulating the polarization and migration of Siglec-F^+^ neutrophils through the actions of SAA3 and CXCL1.

**Figure 7:**
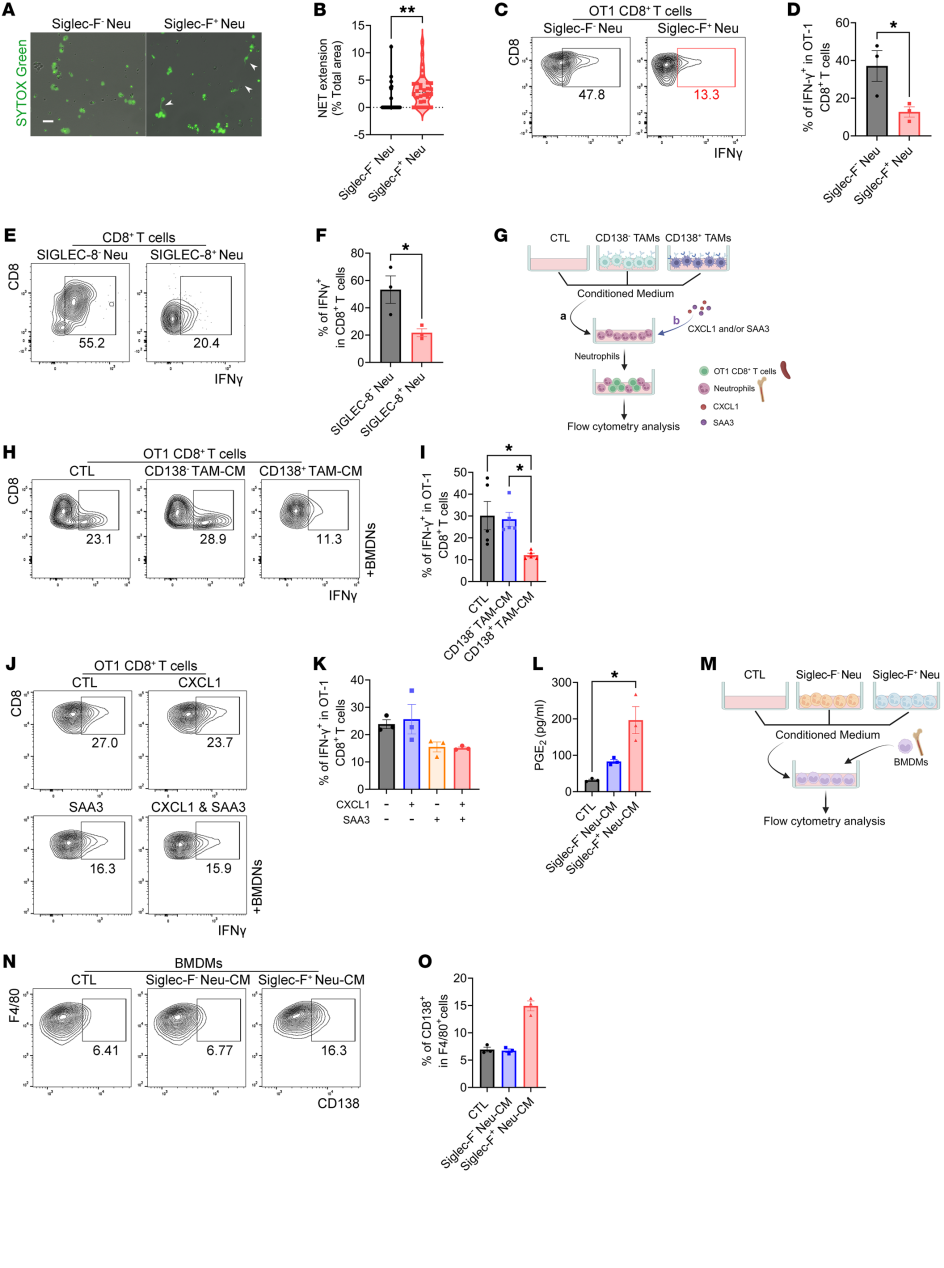
A feed-forward loop between CD138^+^ TAMs and Siglec-F^+^ neutrophils drives tumor immune evasion by inhibiting the antitumor effects of CD8^+^ T cells. (**A**) Representative images showing NET formation (white arrowheads) in Siglec-F^+^ and Siglec-F^–^ neutrophils isolated from orthotopic tumors. Scale bar: 20 μm. (**B**) Quantification of data from **A**, calculating the percentage of the total area covered by the SYTOX Green^+^ region (*n* = 30 per group). (**C**) Plots depicting IFN-γ production by OT1 CD8^+^ T cells from cocultures of splenocytes from OT1 transgenic mice with either Siglec-F^–^ or Siglec-F^+^ neutrophils sorted from orthotopic tumors. (**D**) Quantification of data from **C** (*n* = 3 per group). (**E**) Images depicting IFN-γ production by activated CD8^+^ T cells from cocultures involving CD8^+^ T cells derived from human peripheral blood and either tumor-infiltrating SIGLEC-8^–^ or SIGLEC-8^+^ neutrophils in patients with PDAC. (**F**) Quantification of data from **E** (*n* = 3 per group). (**G**) Schematic illustrating the cocultures of splenocytes isolated from OT1 transgenic mice with BMDNs treated with CM derived from CD138^+^ or CD138^–^ TAMs sorted from orthotopic tumors, as well as with CXCL1 and/or SAA3. (**H** and **J**) Plots displaying IFN-γ production by OT1 CD8^+^ T cells from the indicated coculture groups upon treatment with CM (**H**) or CXCL1 and/or SAA3 (**J**). (**I**) Quantification of data from **H** (*n* = 5 per group). (**K**) Quantification of data from **J** (*n* = 3 per group). (**L**) Quantification of PGE_2_ levels in CM derived from Siglec-F^–^ and Siglec-F^+^ neutrophils sorted from orthotopic tumors (*n* = 3 per group). (**M**) Schematic illustrating the cultures of BMDMs upon treatment with CM derived from Siglec-F^+^ or Siglec-F^–^ neutrophils sorted from orthotopic tumors. (**N**) Images showing the percentages of CD138^+^ macrophages in BMDM cultures treated with CM. (**O**) Quantification of data from **N** (*n* = 3 per group). *P < 0.05 and **P < 0.01 by unpaired 2-tailed Student’s *t* test (**B**, **D**, and **F**) and by Kruskal-Wallis test with Dunn’s multiple-comparison test (**I** and **L**). Data represent mean ± SEM.

We next explored whether and how Siglec-F^+^ neutrophils promote immune evasion in PDAC. Analysis of bulk RNA-seq data revealed that genes upregulated in Siglec-F^+^ neutrophils were significantly enriched in pathways related to arachidonic acid metabolism and NET formation, as defined by KEGG terms (Figure 6M and Supplemental Table 15). Furthermore, Siglec-F^+^ neutrophils exhibited a greater capacity for NET formation in in vitro cultures compared with their Siglec-F^–^ counterparts (Figure 7, A and B). NET formation has been implicated in the exclusion and dysfunction of CD8^+^ T cells within the TME (53, 54). Notably, our results demonstrated the activation of CD8^+^ T cells in orthotopic tumors following the depletion of CD138^+^ TAMs via the genetic ablation of IL-34/syndecan-1 signaling (Figure 4, N–R, and Supplemental Figure 10H). Thus, we examined whether Siglec-F^+^ neutrophils exert direct effects on CD8^+^ T cells. To this end, we established a coculture system utilizing OVA-activated OT1 CD8^+^ T cells and neutrophils isolated from orthotopic tumors. Flow cytometry analysis showed a significant reduction in IFN-γ levels in OT1 CD8^+^ T cells when cocultured with Siglec-F^+^ neutrophils, as opposed to those cocultured with Siglec-F^–^ neutrophils (Figure 7, C and D). Importantly, SIGLEC-8^+^ neutrophils isolated from the tumor tissues of patients with PDAC exhibited similar inhibitory effects on CD8^+^ T cells (Figure 7, E and F). This suggests that Siglec-F^+^ neutrophils possess the ability to impair the cytotoxicity of CD8^+^ T cells. To investigate whether CD138^+^ TAMs hinder the cytotoxicity of CD8^+^ T cells by modulating the properties of neutrophils, BMDNs exposed to CM from either CD138^+^ or CD138^–^ TAMs were introduced into the coculture system (Figure 7G). It was observed that only neutrophils primed by CD138^+^ TAM-CM were capable of reducing IFN-γ levels in OT1 CD8^+^ T cells (Figure 7, H and I). Notably, neutrophils treated with SAA3 (Figure 7G), a factor that is highly expressed by CD138^+^ TAMs, exhibited similar immunosuppressive effects on CD8^+^ T cells (Figure 7, J and K). These findings indicate that CD138^+^ TAMs impair the antitumor functions of CD8^+^ T cells by stimulating the polarization of Siglec-F^+^ neutrophils via the influence of SAA3. It is widely recognized that PGE_2_ is a major metabolite of arachidonic acid metabolism (55), leading to the hypothesis that these cells inversely upregulate the expression of syndecan-1 on TAMs by secreting PGE_2_, thereby promoting the generation of CD138^+^ TAMs. ELISA confirmed a marked elevation in PGE_2_ levels in the CM from Siglec-F^+^ neutrophils (Figure 7L). Subsequently, BMDMs isolated from WT mice were exposed to CM from either Siglec-F^+^ or Siglec-F^–^ neutrophils derived from orthotopic tumors (Figure 7M). Flow cytometry analysis revealed a marked increase in CD138 expression in BMDMs in the group treated with Siglec-F^+^ neutrophil-CM compared with those exposed to Siglec-F^–^ neutrophil-CM (Figure 7, N and O). Furthermore, we observed a synergistic effect between CD138^+^ TAMs and Siglec-F^+^ neutrophils on the functional impairment of CD8^+^ T cells in coculture systems, although CD138^+^ TAMs alone did not affect their functions (Supplemental Figure 13). Taken together, these findings demonstrate the presence of a feed-forward loop between CD138^+^ TAMs and Siglec-F^+^ neutrophils in the TME, which results in immune evasion in PDAC by suppressing the antitumor activities of CD8^+^ T cells. We then investigated the presence of this loop in other cancer types. The results indicated a significant increase in the abundance of CD138^+^ TAMs and SIGLEC-8^+^ neutrophils in the tumor tissues of patients with hepatocellular carcinoma (HCC). In contrast, such an increase was not observed in patients with intrahepatic cholangiocarcinoma (ICC) (Supplemental Figure 14). Given that both IL-34 and PGE_2_ levels were elevated and correlated with tumor progression and metastasis in HCC, but not in ICC (56, 57), we speculate that the CD138^+^ TAM/Siglec-F^+^ neutrophil axis may have developed in cancer types characterized by elevated levels of both IL-34 and PGE_2_.

### Anti–IL-34 antibodies in combination with anti–PD-1 immunotherapy effectively abrogate the progression of PDAC.

**Figure 8:**
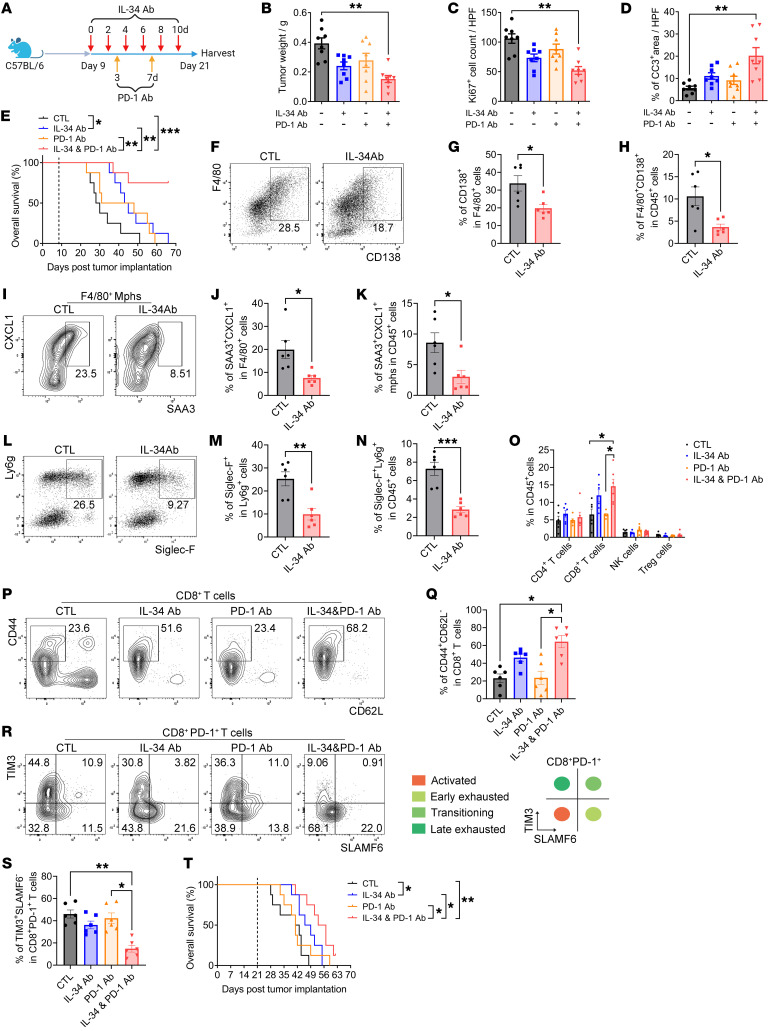
Anti–IL-34 antibodies abrogate tumor progression and enhance responses to anti–PD-1 immunotherapy in PDAC by disrupting the CD138^+^ TAM/Siglec-F^+^ neutrophil axis. (**A**) Experimental design employed to evaluate the therapeutic efficacy of anti–IL-34 antibodies in conjunction with anti–PD-1 antibodies in PDAC. (**B**) Quantification of tumor weight (*n* = 8 per group). (**C** and **D**) Quantification of Ki67^+^ cell counts (**C**) and the percentage of CC3^+^ area (**D**) per ×40 field in tumor sections (*n* = 8 per group). (**E**) Overall survival probabilities of mice (*n* = 8 per group). The dashed line indicates the time point when the combination therapy commenced. (**F** and **I**) Flow cytometry images showing CD138^+^F4/80^+^ (**F**) and SAA3^+^CXCL1^+^ (**I**) macrophages in the tumor tissues of mice following anti–IL-34 antibody treatment. (**G** and **H**) Quantification of data from **F** (*n* = 6 per group). (**J** and **K**) Quantification of data from **I** (*n* = 6 per group). (**L**) Representative plots illustrating the presence of Siglec-F^+^ neutrophils in the tumor tissues of mice upon treatment with anti–IL-34 antibodies. (**M** and **N**) Quantification of data from **L** (*n* = 6 per group). (**O**) Quantification of the frequencies of effector cell subsets among CD45^+^ cells in tumor tissues (*n* = 6 per group). (**P** and **R**) Representative images depicting CD44^+^CD62L^–^ effector (**P**) and TIM3^+^SLAMF6^–^PD-1^+^ late-exhausted (**R**) CD8^+^ T cells within the tumor tissues. (**Q**) Quantification of data from **P** (*n* = 6 per group). (**S**) Quantification of data from **R** (*n* = 6 per group). (**T**) Overall survival probabilities of mice (*n* = 8 per group). The dashed line indicates the time point when the combination therapy commenced. *P < 0.05, **P < 0.01, and ***P < 0.001 by Kruskal-Wallis test with Dunn’s multiple-comparison test (**B**–**D**, **O**, **Q**, and **S**), by log-rank analysis (**E** and **T**), and by unpaired 2-tailed Student’s *t* test (**G**, **H**, **J**, **K**, **M**, and **N**). Data represent mean ± SEM.

Next, we investigated the impact of disrupting the feed-forward loop between CD138^+^ TAMs and Siglec-F^+^ neutrophils by blocking IL-34/syndecan-1 signaling in PDAC progression. Given that flow cytometry results indicated that these 2 subsets prominently emerged 14 days after tumor implantation (Supplemental Figure 15), anti–IL-34 antibodies were administered intraperitoneally to orthotopic KPC mice twice daily, commencing 9 days after tumor injection, with tumor tissues harvested 21 days after implantation (Figure 8A). A marked reduction in tumor weight was observed in the anti–IL-34 antibody treatment group (Figure 8B and Supplemental Figure 16A). Pathological analysis revealed that treatment with anti–IL-34 antibodies led to decreased cell proliferation and increased apoptosis within the tumor tissues (Figure 8, C and D, and Supplemental Figure 16B). Notably, survival analysis demonstrated a significant increase in overall survival times for mice receiving anti–IL-34 antibody treatment compared with the control group (Figure 8E).

To determine whether the therapeutic effects of anti–IL-34 antibodies depend on the blockade of the CD138^+^ TAM/Siglec-F^+^ neutrophil axis, we examined the cellular compositions within the TME of orthotopic KPC mice following treatment with anti–IL-34 antibodies. The results demonstrated a significant reduction in the percentage of CD138^+^ TAMs and Siglec-F^+^ neutrophils (Figure 8, F–N), accompanied by a notable increase in the frequencies of CD8^+^ T cells after treatment with anti–IL-34 antibodies, although other effector cell subsets did not exhibit significant alterations (Figure 8O). Subsequent phenotypic analysis revealed an expansion of effector CD8^+^ T cells; nevertheless, a considerable proportion of CD8^+^ T cells displayed a late-exhausted state, and NK cells were not activated within the TME (Figure 8, P–S, and Supplemental Figure 16, C–G). This finding indicates that T cell exhaustion occurs in the PDAC TME during treatment with anti–IL-34 antibodies. Thus, a therapeutic strategy combining anti–IL-34 antibodies with anti–PD-1 immunotherapy was evaluated in orthotopic KPC mice (Figure 8A). The results revealed a notable delay in tumor progression in mice receiving the combination therapy compared with those treated with single agents (Figure 8, B–D, and Supplemental Figure 16, A and B). More importantly, mice treated with the combination therapy exhibited remarkably longer survival times than those in the control groups (Figure 8E). Subsequently, we assessed the immune status of the TME in mice that underwent combination therapy. The results revealed a marked increase in the percentages of CD8^+^ T cells and effector CD8^+^ T cells, alongside a substantial reduction in late-exhausted CD8^+^ T cells (Figure 8, O–S, and Supplemental Figure 16, C–E). Furthermore, NK cells within the TME did not exhibit significant activation (Supplemental Figure 16, F and G). These observations demonstrate that anti–PD-1 immunotherapy enhances therapeutic efficacy against PDAC by reversing CD8^+^ T cell exhaustion within the TME when combined with anti–IL-34 antibodies. Notably, the combination therapy continued to prolong overall survival times in orthotopic KPC mice, even when initiated 21 days after tumor implantation (Figure 8T). Considering that CD138^+^ TAMs drive the polarization of Siglec-F^+^ neutrophils through the activation of the SAA3/FPR2/p38 MAPK pathway, we tested a therapeutic strategy that combined anti–IL-34 antibodies, anti–PD-1 antibodies, and WRW4 (a FPR2 antagonist) in orthotopic KPC mice. Unexpectedly, survival analysis revealed no improvement in overall survival times in mice when WRW4 was introduced into the therapeutic strategy (Supplemental Figure 16H). Collectively, these findings indicate that anti–IL-34 antibodies dramatically enhance the management of PDAC by impeding the immunosuppressive feed-forward loop between CD138^+^ TAMs and Siglec-F^+^ neutrophils and demonstrate improved therapeutic effects, particularly when combined with anti–PD-1 immunotherapy.

## Discussion

Over the past few decades, numerous studies have explored the potential of immunotherapy to improve overall survival in patients with PDAC and in mouse models (58). However, the majority of immunotherapy strategies, whether administered alone or in combination, have proven ineffective, primarily due to the tumor’s ability to evade the immune system. This study identifies the emergence of a unique population of CD138^+^ TAMs that expands in both PDAC patients and mouse models, characterized by proinflammatory and neutrophil-chemotactic activity. This expansion correlates with tumor immune escape and unfavorable outcomes in patients and is crucial for immune evasion and disease progression in mice. Through phenotypic and functional analyses, we determined that these cells establish a reciprocal relationship with immunosuppressive Siglec-F^+^ neutrophils by secreting SAA3 and CXCL1. This interaction facilitates PDAC immune evasion by hindering the cytotoxic actions of CD8^+^ T cells. By blocking the generation of CD138^+^ TAMs, the progression of pancreatic tumors can be prevented, particularly when combined with anti–PD-1 immunotherapy. Thus, our findings support a combinatorial therapeutic strategy to impede the advancement of PDAC.

It should be noted that although both IL-34 and PGE_2_ have been reported to promote M2 polarization of macrophages in acute inflammation and several cancer types (17, 18, 24, 59, 60), their combined actions have rarely been reported. To the best of our knowledge, this study provides the initial documentation of a localized synergy between IL-34/syndecan-1 and PGE_2_/EP2 signaling in the polarization of tumor-infiltrating monocytes into proinflammatory CD138^+^ TAMs. The underlying mechanism involves the activation of the PI3K/Akt/NF-κB signaling pathway by IL-34, in conjunction with the EPAC/Rap1 pathway stimulated by PGE_2_. Supporting this model, previous studies have demonstrated the following: (a) upregulation of both IL-34 and COX-2 in PDAC has been observed (35–37, 61). (b) PGE_2_ exerts regulatory effects on syndecan-1 expression in macrophages (24, 62). (c) Syndecan-1 acts as a functional receptor for IL-34 in macrophages (19, 34, 59). (d) Macrophages induced by IL-34 promote the polarization of Th1/Th17 cells in rheumatoid arthritis, a chronic inflammatory environment (32). (e) PGE_2_ facilitates tumor immune escape through the impairment of antigen presentation by Cxcl9/10^+^ inflammatory monocytes (63). Thus, in response to the chronic, dysregulated, persistent, and unresolved inflammatory state within the PDAC TME (64), tumor-infiltrating monocytes likely undergo a distinct differentiation program, giving rise to an atypical proinflammatory macrophage subtype in PDAC.

Both TAMs and tumor-infiltrating neutrophils play crucial roles in tumor immune escape (65). Several studies have documented a bidirectional interplay between these 2 myeloid cell types within tumors (66, 67). However, the specific interactions between TAMs and neutrophils in the TME of PDAC have not been extensively explored. This study emphasizes a role of SAA3, secreted by CD138^+^ TAMs, in polarizing tumor-infiltrating neutrophils toward an immunosuppressive Siglec-F^+^ phenotype, characterized by enhanced synthesis of PGE_2_ and the release of NETs. This process occurs through the binding of SAA3 to FPR2, subsequently activating the p38 MAPK signaling pathway in neutrophils. In turn, Siglec-F^+^ neutrophils facilitate syndecan-1 expression via PGE_2_ production, which further enhances IL-34/syndecan-1 signaling in TAMs. This interaction creates a feed-forward loop that ultimately drives immune evasion in PDAC. Notably, the emergence of a small number of CD138^+^ macrophages in adjacent benign tissues of PDAC patients and in the pancreatic tissues of WT mice suggests that this feed-forward loop is initially driven by gradually increasing levels of IL-34 secreted by tumor cells during the early stages of pancreatic cancer. Furthermore, the elevated expression levels of *Ptgs2* in tumor-infiltrating neutrophils prior to the establishment of this loop suggest that these cells provide the initiating PGE_2_ signal necessary to trigger this loop.

Our preclinical studies demonstrate that the administration of anti–IL-34 antibodies effectively inhibits the progression of pancreatic tumors by enhancing the antitumor responses of effector CD8^+^ T cells. Notably, these antibodies exhibit improved therapeutic effects when combined with anti–PD-1 immunotherapy. In contrast, anti–PD-1 antibodies administered independently do not enhance overall survival in mice. Similar trends have been reported in clinical trials, where PDAC tumors show limited responsiveness to anti–PD-1 immunotherapies (58, 68). Thus, these findings indicate that targeting CD138^+^ TAMs through the blockade of IL-34/syndecan-1 signaling facilitates a transition of the PDAC TME from immunosuppressive to immunoreactive. This transition may enable anti–IL-34 antibodies to effectively synergize with anti–PD-1 immunotherapy, potentially converting entirely unresponsive PDAC tumors into responsive ones in clinical settings. Our findings indicate that the introduction of the FPR2 antagonist into the combination therapeutic strategy did not confer any benefit in overall survival times in mice. This lack of improvement may be attributed to the presence of multiple ligands of FPR2, including lipoxin A4, which has been reported to play inhibitory roles in tumor progression and metastasis of PDAC (69, 70).

In summary, we have identified a unique population of CD138^+^ TAMs elicited by IL-34 and PGE_2_ in both PDAC patients and mouse models. This specific TAM subset establishes a feed-forward loop with immunosuppressive PGE_2_-secreting Siglec-F^+^ neutrophils through the actions of SAA3 and CXCL1, which leads to immune evasion and thereby fosters a protumor TME. Targeting this population with anti–IL-34 antibodies may yield clinical benefits for PDAC patients, especially when combined with anti–PD-1 immunotherapy. Therefore, the combined application of anti–IL-34 and anti–PD-1 antibodies may sustain antitumor responses, providing a therapeutic avenue for improved PDAC management.

## Methods

Additional details may be found in Supplemental Methods.

### Sex as a biological variable.

This study included both male and female patients, as well as healthy donors, ensuring that there was no bias in the grouping. The results were consistent regardless of gender. All animal experiments were conducted using male mice. Given that estrogen might exert a suppressive effect on tumor progression, the male mouse represents a widely accepted and appropriate model for exploring the mechanisms of tumor progression and evaluating cancer therapeutics, in the absence of this confounding hormonal influence.

### Patient samples.

Fresh tissue and serum samples were collected from patients with PDAC in cohort 1, all of whom underwent surgical resection at The First Affiliated Hospital of Zhejiang University School of Medicine. This cohort comprised 103 patients with PDAC who had not received any prior treatments. Additionally, 41 paired adjacent benign and tumor tissues from patients with PDAC in cohort 2 along with 156 unpaired tumor tissues from patients in cohort 3 were utilized to create 2 distinct tissue arrays at Wuhan Servicebio Technology. Detailed patient information for cohort 3, which includes data on 114 patients with available prognostic information, is provided in Supplemental Table 1. Patients in cohort 3 were stratified into groups according to the abundance of CD138^+^ TAMs and/or the frequency of SIGLEC-8^+^ neutrophils, utilizing the optimal *P* value cutoff determined by the Mann-Whitney test. A total of 13 patients with HCC and 15 patients with ICC underwent surgical resection at The First Affiliated Hospital of Zhejiang University School of Medicine, from which paired adjacent benign and tumor tissues were collected for the preparation of tissue sections.

### Animals.

Male *C57BL/6* mice aged 6 to 8 weeks were purchased from the Model Animal Research Center of Nanjing University, Nanjing, China. The *Kras^LSL-G12D^*, *Trp53^LSL-R172H^*, and *Pdx-1-cre* genetically engineered mice were procured from The Jackson Laboratory and subsequently crossed to generate the spontaneous KPC mouse model. *Kras^LSL-G12D^ TgfβR2^fl/fl^ Ptf1a-cre* (KTC) mice were provided by Hideaki Ijichi (The University of Tokyo, Tokyo, Japan) and Harold L. Moses (Vanderbilt-Ingram Cancer Center, Nashville, Tennessee, USA). *Ptger2-flox* (strain T018685), *Sdc1-flox* (strain T015943), and *Csf1r-Cre* (strain T005640) mice were acquired from GemPharmatech. *Ptger2-flox* and *Sdc1-flox* mice were crossed with *Csf1r-Cre* mice to generate *Ptger2^fl/fl^ Csf1r-Cre* (Ptger2-cKO) and *Sdc1^fl/fl^ Csf1r-Cre* (Sdc1-cKO) mice, respectively. All mice were maintained under specific pathogen–free conditions at the Experimental Animal Center of The First Affiliated Hospital of Zhejiang University School of Medicine.

For the orthotopic tumor models, 5 × 10^5^ KPC cells were resuspended in cold PBS and mixed at a 1:1 dilution with Matrigel (Corning) to achieve a final volume of 25 μL, which was then injected into the pancreas. Mice were euthanized on day 21 after tumor implantation, except for those designated for survival analysis. The endpoint of the survival analysis was defined as the time at which all mice in either the control or experimental group had succumbed.

### Anti–IL-34, anti–PD-1, and/or WRW4 treatment in vivo.

Mice were intraperitoneally injected with 200 μg of anti–IL-34 antibody (catalog MAB5195, R&D Systems) every other day, totaling 6 doses, starting on day 9 after the orthotopic inoculation of 5 × 10^5^ KPC cells. Anti–PD-1 antibody (200 μg/mice, catalog BE0146, Bio X Cell) was administered intraperitoneally on days 12 and 16 after tumor implantation. WRW4 (1 mg/kg, MedChemExpress) was injected intraperitoneally daily, commencing on day 9 after tumor implantation. At 21 days after injection, all mice were euthanized, except for those designated for survival analysis. The endpoint of the survival analysis was determined as the time at which all mice in the control group had perished. Tumor tissues were collected for flow cytometry analysis or IHC staining. For the survival analysis of the combination therapy against advanced tumors, the administration of anti–IL-34 antibodies began on day 21, while anti–PD-1 antibodies were injected starting on day 24 following tumor implantation.

### Statistics.

Statistical analyses were conducted using GraphPad Prism 9. Results are presented as mean ± SEM, with significance evaluated through unpaired or paired 2-tailed Student’s *t* tests, unless otherwise specified. *P* values less than 0.05 were considered statistically significant. For correlation assessments, either Pearson’s or Spearman’s correlation analyses were utilized. Differences in survival analysis were determined using the log-rank test. The optimal cutoff point for patients in cohort 3 was calculated using Mann-Whitney tests within SPSS Statistics software. Bioinformatics statistical analyses were performed in R (v4.4.1). For comparisons between 2 groups, the Wilcoxon’s rank-sum test was employed to assess differences in population means, with significance defined as a *P* value less than 0.05, unless otherwise indicated.

### Study approval.

All participants provided signed informed consent, and the research protocol was approved by the Ethics Committee of The First Affiliated Hospital of Zhejiang University School of Medicine (IIT20210023B-R1). The animal experiments were conducted in strict accordance with the guidelines and standards approved by the Institutional Animal Care and Use Committee.

### Data availability.

The datasets generated and/or analyzed during the current study are available in the Genome Sequence Archive under accession number CRA017890. The data reanalyzed for this study can be accessed in the Gene Expression Omnibus using the accession code GSE217846, which pertains to the scRNA-seq of blood samples from orthotopic KPC mice. Additionally, the scRNA-seq data of healthy pancreas and acute pancreatitis in mice can be found in the European Nucleotide Archive under the project identifier PRJNA978570. All code utilized in this study is accessible at https://jhuanglab.github.io/sdc1_mac/ (commit ID is aa92608). Values for all data points in graphs are reported in the Supporting Data Values file.

## Author contributions

CW, QZ, T Lu, and T Liang designed the experiments and interpreted the data. CW, FL, YM, and JT performed most of the experiments. FL performed clinical relevance analysis. JH, DZ, YL, TZ, and JL performed the bioinformatics analysis. FS and HS assisted in some experiments. CW and T Liang wrote the manuscript and provided overall guidance. The order of co–first authors CW, QZ, and JH reflects the extent to which each author contributed to the key developments in this work.

## Conflict of interest

The authors have declared that no conflict of interest exists.

## Funding support

- National Key Research and Development Program of China (2020YFA0804300 to QZ).
- National Natural Science Foundation of China (82188102 and U20A20378 to T Liang, 82003016 to CW, and 92474310, 32321002, and 82273338 to QZ).
- Natural Science Foundation of Zhejiang Province (LQ21H160017 to CW).
- Fundamental and Interdisciplinary Disciplines Breakthrough Plan of the Ministry of Education of China (JYB2025XDXM611 to T Liang).

## Supplementary Material

Supplemental data

Unedited blot and gel images

Supplemental table 1

Supplemental table 2

Supplemental table 3

Supplemental table 4

Supplemental table 5

Supplemental table 6

Supplemental table 7

Supplemental table 8

Supplemental table 9

Supplemental table 10

Supplemental table 11

Supplemental table 12

Supplemental table 13

Supplemental table 14

Supplemental table 15

Supporting data values

## Version 1 03/12/2026

In-Press Preview

## Version 2 05/01/2026

Electronic publication
