# Supplementary material for: A CD138+ tumor-associated macrophage/Siglec-F+ neutrophil feed-forward loop promotes immune evasion in pancreatic cancer
Source: J Clin Invest. 2026 Mar 12;136(9):e199516. doi: 10.1172/JCI199516 (PMC13132371; doi:10.1172/JCI199516)
Supplement: Supplemental data [file jci-136-199516-s206.pdf]

Supplemental Material

Manuscript

A CD138$^+$ tumor-associated macrophage-Siglec-F$^+$ neutrophil feedforward loop promotes immune evasion in pancreatic cancer

(Wang et al.)

Supplemental Methods

Cell lines

The KPC cell line was generously provided by Prof. Raghu Kalluri from the MD Anderson Cancer Center in Houston, Texas, USA. This cell line was derived from spontaneous tumors in the *Kras*$^{LSL\text{-}G12D}$*; Trp53*$^{LSL\text{-}R172H}$*; Pdx-1-cre* mouse model. KPC cells were cultured in RPMI 1640 medium supplemented with 10% FBS and 1% penicillin/streptomycin. Prior to the experiments, the cells were routinely evaluated for mycoplasma contamination.

Sorting of CD138$^+$ TAMs and Siglec-F$^+$(SIGLEC-8$^+$) neutrophils

The digestion buffer was prepared in DMEM medium supplemented with 2% FBS, 0.6 mg/mL collagenase IV (Sigma), and 0.01 mg/mL DNase I (Sigma). Tumor tissues were minced in a 10cm Petri dish, resuspended in 5mL of digestion buffer, and incubated on

a shaker at 37°C for 30 minutes. The digested tissues were filtered through a 70μm mesh filter and further separated using 36% Percoll to obtain single-cell suspensions. These cells were then resuspended in PBS and pre-incubated with anti-mouse CD16/CD32 Fc Block (BD Biosciences) for 10 minutes at 4°C, followed by incubation with antibodies against CD14 (Cat 562692), F4/80 (Cat 565410), CD138 (Cat 550805 for human and Cat 564511 for mouse), CD66B (Cat 555724), Ly6g (Cat 560599), Siglec-F (Cat 562681), and SIGLEC-8 (Cat 347105) for 30 minutes at 4°C. All antibodies were procured from BD Biosciences, except for SIGLEC-8, which was purchased from BioLegend. Subsequently, the samples were sorted using a MoFlo Astrios EQ Cell Sorter (Beckman Coulter, USA).

Flow cytometry analysis

Cells from different experiments were stained with fluorescence-labeled antibody cocktails for 30 minutes at 4°C and subsequently analyzed using a BD LSRFortessa instrument. For intracellular factor staining, the cells were incubated with Brefeldin A Solution (BioLegend) for 4 hours. Following this, the cells were fixed and permeabilized using a Fixation/Permeabilization Kit (BD Biosciences). The data were analyzed using FlowJo software.

Generation of CD45.1/CD45.2 chimeric mice with orthotopic KPC tumors

Eight-week-old *C57BL/6* (CD45.2) mice were lethally irradiated with two doses of 6.5 Gy, followed by reconstitution with $1X10^6$ donor bone marrow cells from *C57BL/6*-

Ly5.1 (CD45.1) mice. After reconstitution, all mice received prophylactic antibiotics in their drinking water and were housed in a specific pathogen-free environment. A week post-reconstitution, $5X10^5$ KPC cells were injected into the pancreas of the CD45.1/CD45.2 chimeric mice. At 21 days post-injection, all mice were sacrificed, and tumor tissues were collected for flow cytometric analysis.

Adoptive transfer assay

$CD138^+$ and $CD138^-$ TAMs were isolated from primary tumors of $CD45.1^+$ mice with orthotopic KPC tumors, following established protocols. $CD45.2^+$ mice received an intravenous injection of $5X10^5$ $CD138^+$ TAMs starting on Day 10 post-orthotopic injection of KPC cells, with injections repeated every 3-4 days for a total of three doses. Control groups received intravenous injections of either $5X10^5$ $CD138^-$ TAMs or an equivalent volume of PBS, adhering to the same dosing regimen. At 21 days post-injection, all mice were euthanized, except for those designated for survival analysis. The endpoint of the survival analysis was defined as the time at which all mice in the experimental group had succumbed. Tumor tissues were collected for flow cytometry analysis, immunohistochemical staining and scRNA-seq.

Generation of a KPC/Sdc1-cKO chimeric mouse model

Eight-week-old spontaneous KPC mice were subjected to lethal irradiation, receiving two doses of 6.5 Gy, and subsequently reconstituted with $1X10^6$ donor bone marrow cells derived from Sdc1-cKO mice. Following reconstitution, all mice were

administered prophylactic antibiotics via their drinking water and maintained in a specific pathogen-free environment. Eight weeks post-reconstitution, the mice were euthanized, and pancreatic tissues were harvested for the evaluation of tumorigenesis.

In vitro differentiation of CD138$^+$ macrophages

Bone marrow cells from male C57BL/6 mice aged 6 to 8 weeks were collected by crushing the femurs and tibias in 10mL of sterile PBS, followed by filtration through a 70μm mesh filter. Red blood cells were removed using ACK Lysis Buffer (BD Biosciences). For the differentiation of BMDMs, cells were counted and seeded in DMEM medium supplemented with 10% FBS, 2% antibiotics, and 20ng/mL M-CSF (Selleck). Three days post-seeding, half of the culture medium was replaced. On day 5, fresh medium was added to the cells. At day 7 after plating, cells were stimulated with 250ng/mL IL-34 and/or 1μM PGE_2. After 24 hours, the cells were harvested and analyzed by flow cytometry and RT-PCR. For stimulation with conditioned medium (CM), Siglec-F$^+$ or Siglec-F$^-$ neutrophils sorted from tumor tissues of mice with orthotopic KPC tumors were cultured in DMEM medium supplemented with 10% FBS and 2% antibiotics for 24 hours. CM was collected and centrifuged for 5 minutes at 200g to remove cellular debris. In rescue experiments, 10μM SC-51089 (EP1 antagonist), 10μM PF-04418948 (EP2 antagonist), 10μM L-798106 (EP3 antagonist), 10μM GW627368X (EP4 antagonist), 10μM ESI-08 (EPAC/Rap1 pathway antagonist), 10μM PI3K/AKT-IN-1 (PI3K-Akt pathway inhibitor), 10μM JSH-23 (NF-kappa B pathway inhibitor), and 1μM synstatin (a selective inhibitor of syndecan-1) were

utilized. All reagents were purchased from MedChemExpress. BMDMs derived from Sdc1-cKO mice were used to evaluate the roles of syndecan-1 in the differentiation of $CD138^+$ TAMs.

In vitro differentiation of Siglec-F^+ (SIGLEC-8^+) neutrophils

Bone marrow-derived neutrophils (BMDNs) were isolated using a $Ly6g^+$ neutrophil isolation kit (Miltenyi Biotec). The cells were subsequently stimulated with 100ng/ml CXCL1 and/or 200ng/ml SAA3 for 24 hours and analyzed via flow cytometry. In rescue experiments, 10μM WRW4 (FPR2 antagonist) and 40μM p38 MAPK-IN-1 (p38 MAPK pathway inhibitor) were utilized. All reagents were procured from MedChemExpress. For stimulation with CM, $CD138^+$ or $CD138^-$ TAMs sorted from the tumor tissues of mice with orthotopic KPC tumors were cultured in DMEM medium supplemented with 10% FBS and 2% antibiotics for 24 hours. The CM was subsequently collected and centrifuged for 5 minutes at 200g to remove cellular debris. Following this, BMDNs were cultured in the presence of the CM for 24 hours and analyzed using flow cytometry. In rescue experiments, 10μg/ml anti-SAA3 antibody (Cat A11948, ABclonal Biotechnology) was introduced in the BMDN culture system.

For the in vitro polarization of SIGLEC-8^+ neutrophils, PBDNs were isolated from healthy donors using a MACSxpress® whole blood neutrophil isolation kit (Miltenyi Biotec). $CD138^+$ and $CD138^-$ TAMs sorted from patients with PDAC were cultured in DMEM medium supplemented with 10% FBS and 2% antibiotics for 24 hours. The CM was subsequently collected and centrifuged at 200g for 5 minutes to remove

cellular debris. Following this, PBDNs were cultured in the presence of the CM for 24 hours and analyzed using flow cytometry.

Lentivirus production and transfection

The pLV3-U6-Il34-sgRNA1/2/3-Cas9-EGFP-Puro and pSLenti-EF1-EGFP-CMV-OVAL-3xFLAG-WPRE plasmids were co-transfected into HEK-293T cells alongside the packaging plasmid psPAX2 and the envelope plasmid pMD2G, utilizing the PEI transfection reagent (Beyotime). Virus particles were collected 48 hours post co-transfection and subsequently employed to infect KPC cells. The cells were harvested three days following injection for Western blot validation, in vitro cultures, and orthotopic implantation.

Western blot analysis

Bone marrow cells from Ptger2-cKO and control mice, as well as KPC cells infected with lentivirus, were lysed using RIPA lysis buffer (Beyotime) supplemented with a protease and phosphatase inhibitor cocktail (Beyotime) and phenylmethanesulfonyl fluoride (PMSF, Beyotime) at 4°C for one hour. The protein concentration was quantified using a BCA Protein Assay Reagent (Beyotime). The proteins were subsequently separated by 10% SDS-PAGE and transferred to a polyvinylidene fluoride membrane (PVDF, Millipore). Following the blocking of the membrane with 5% nonfat dry milk, it was incubated overnight with a primary antibody against PTGER2 (Cat ab167171, Abcam) or IL-34 (Cat PA5-95624, Thermofisher), followed by a one-hour

incubation with a horseradish peroxidase (HRP)-conjugated anti-rabbit secondary antibody (Cat A0208, Beyotime). Finally, the membrane was exposed using a Super-sensitive ECL chemiluminescent substrate (Biosharp) and a ChemiScope Touch machine (Clinx) to detect immunoreactive bands.

ELISA

Serum samples were collected from PDAC patients in Cohort 1, healthy donors, control mice, and orthotopic KPC mice. Additionally, CM derived from tumor-infiltrating Siglec-F$^+$ and Siglec-F$^-$ neutrophils were also obtained. The levels of PGE_2 in these samples were measured using an ELISA kit (R&D Systems) in accordance with the manufacturer's instructions.

Morphology, phagocytosis, and migration of CD138$^+$ macrophages

Macrophages, including sorted CD138$^+$ and CD138$^-$ TAMs, as well as in-vitro differentiated CD138$^+$ and CD138$^-$ macrophages, were seeded onto cell slides (ThermoFisher) at a density of 1X10^5 per slide and cultured in DMEM medium supplemented with 10% FBS and 2% antibiotics. After 24 hours of culture, the cells were fixed with 4% paraformaldehyde for 30 minutes and subsequently stained with Alexa Fluor^{TM} 647 phalloidin (ThermoFisher) for 60 minutes at room temperature. The morphology of these cells was observed using a confocal laser scanning microscope (TCS SP8 CARS, Leica).

To investigate the phagocytic activity of macrophages, the cells were seeded in 96-well

plates at a concentration of 1X10^5 cells per well. pHrodo™ Deep Red E.coli BioParticles™ conjugates (ThermoFisher) were added to the cells, followed by a 2-hour incubation at 37℃. The phagocytic activity was assessed using a Multi-Mode Microplate Reader (SpectraMax i3x, Molecular Devices) at a wavelength of 640/655nm.

To examine the migration of macrophages, a transwell assay was conducted. The cells were placed in the upper chambers of a transwell apparatus with 8μm pore sizes (Corning) using DMEM medium containing 0.5% FBS and 2% antibiotics. The lower chambers contained DMEM medium supplemented with 10% FBS and 2% antibiotics. After 18 hours, the cells that migrated through the membrane were stained with a 0.5% crystal violet solution (Beyotime) and quantified by counting the average number of cells in five high-power fields (HPF) per well.

Transwell assay of BMDNs

BMDNs were isolated and seeded in the upper chambers of a transwell apparatus with 3μm pore sizes (Corning) at a density of 1X10^6 cells per well. The lower chambers contained CM from $CD138^+$ or $CD138^-$ TAMs diluted at a 1:1 ratio with DMEM medium supplemented with 10% FBS and 2% antibiotics. After 2 hours of culture, the cells in the lower chambers were counted.

To assess the chemotaxis of proteins secreted by $CD138^+$ TAMs, DMEM medium containing 10ng/ml CXCL1 and/or 100ng/ml SAA3 was added to the lower chamber of the transwell apparatus.

OT1 $CD8^+$ T cell culture assay

Splenocytes were harvested from OT1 mice and incubated with the OVA peptide (OVA 257-264, MedChemExpress) in RPMI 1640 medium for one hour at 37°C. Subsequently, $1X10^6$ splenocytes were seeded in 12-well plates and co-cultured with either $2X10^5$ $CD138^+$ or $CD138^-$ TAMs, or an equal number of Siglec-F^+ or Siglec-F^- neutrophils isolated from orthotopic tumors, in RPMI 1640 medium supplemented with 10% FBS, 2% antibiotics, and 20ng/ml mouse recombinant IL-2 (MedChemExpress) for 24 hours. Non-adherent cells were then collected to measure IFNγ production in $CD8^+$ T cells.

In the case of Siglec-F^+ neutrophils differentiated from BMDNs, BMDNs were induced in vitro using CXCL1 and/or SAA3 or CM derived from $CD138^+$ or $CD138^-$ TAMs, following established protocols. These cells were then co-cultured with an equal number of OVA peptide-stimulated OT1 splenocytes for 24 hours.

To evaluate the functional impairment of $CD8^+$ T cells, a co-culture system was established using OT1 splenocytes in the presence or absence of $CD138^+$ TAMs and/or Siglec-F^+ neutrophils. Briefly, OT1 splenocytes were labeled with the CellTrace™ Violet Cell Proliferation Kit (ThermoFisher) according to the manufacturer's instructions and subsequently incubated with the OVA peptide. Following this, $1X10^6$ OT1 splenocytes were seeded in 12-well plates and co-cultured with $2X10^5$ $CD138^+$ TAMs and/or an equal number of Siglec-F^+ neutrophils isolated from orthotopic tumors. The co-culture was maintained in RPMI 1640 medium containing 10% FBS, 2%

antibiotics, and 20ng/ml mouse recombinant IL-2 for a duration of 48 hours. Non-adherent cells were then collected for flow cytometric analysis.

Target cell killing assay

To establish co-cultures of OT1 $CD8^+$ T cells, KPC-OVA cells, and $CD138^+$ TAMs and/or Siglec-F^+ neutrophils, OT1 splenocytes were harvested from OT1 mice and incubated with the OVA peptide for five days at 37℃. Subsequently, OT1 $CD8^+$ T cells were purified using the EasySep™ mouse $CD8^+$ T cell isolation kit (Stemcell Technologies). KPC-OVA cells were seeded and allowed to adhere overnight. Following this, OT1 $CD8^+$ T cells were introduced into the culture, either in the presence or absence of $CD138^+$ TAMs and/or Siglec-F^+ neutrophils derived from orthotopic tumors. After 48 hours of co-culture, the cells were harvested via trypsinization and analyzed using flow cytometry.

Human $CD8^+$ T cell culture assay

Human $CD8^+$ T cells were isolated from the peripheral blood of healthy donors using a $CD8^+$ T cell isolation kit (Miltenyi Biotec). Subsequently, $1X10^5$ cells were seeded into each well of 48-well plates alongside an equal number of SIGLEC-8^+ or SIGLEC-8^- neutrophils isolated from the tumor tissues of patients with PDAC. These cells were cultured in RPMI 1640 medium supplemented with 10% FBS, 2% antibiotics, 20ng/ml human recombinant IL-2 (MedChemExpress), and 1% Dynabeads™ Human T-Activator CD3/CD28 (ThermoFisher) for 24 hours. Following this incubation period,

the cells were collected for flow cytometry analysis.

RT-PCR

RNA was extracted using the RNeasy Mini Kit (Qiagen), and cDNAs synthesis was performed with the HiScript II Reverse Transcriptase Kit (Vazyme). Gene expression was quantified using RT-PCR with SYBR Green Master Mix (Vazyme). The primer sequences used for RT-PCR are as follows: *Ptger1* (EP1): Forward Primer, GGGCTTAACCTGAGCCTAGC; Reverse Primer, GTGATGTGCCATTATCGCCTG; *Ptger2* (EP2): Forward Primer, GGAGGACTGCAAGAGTCGTC; Reverse Primer, GCGATGAGATTCCCCAGAACC; *Ptger3* (EP3): Forward Primer, CCGGAGCACTCTGCTGAAG; Reverse Primer, CCCCACTAAGTCGGTGAGC; *Ptger4* (EP4): Forward Primer, ACCATTCCTAGATCGAACCGT; Reverse Primer, CACCACCCCGAAGATGAACAT; *Ptgs2* (COX2): Forward Primer, TTCAACACACTCTATCACTGGC; Reverse Primer, AGAAGCGTTTGCGGTACTCAT.

Immunohistochemistry, Immunofluorescence, and mIHC

Paraffin-embedded samples were sectioned to a thickness of 4μm. Antigen retrieval was performed in a pressure cooker using 0.01M citrate buffer (pH 6.0) for 30 minutes. For immunohistochemistry, specimens were treated with 3% H_2O_2 for 30 minutes, blocked with 5% BSA (Sigma) for 1 hour, and incubated overnight at 4°C with primary antibodies against Ki67 (Cat ab16667, Abcam) and cleaved caspase-3 (Cat MAB835,

R&D systems). Subsequently, the slides were incubated with anti-rabbit IgG, HRP-linked antibody (Cat A0208, Beyotime) for 1 hour, followed by immunodetection using DAB (ZSGB-BIO) according to the manufacturer's instructions. Imaging was performed using a light microscope (Leica DM2500), and data analysis was conducted with ImageJ software (National Institutes of Health).

For immunofluorescence, slides were blocked with 5% BSA for 1 hour, then incubated overnight at 4°C with anti-F4/80 (Cat 70076S, Cell Signaling Technology) and anti-CD138 (Cat ab181789, Abcam) antibodies. The specimens were labeled with Alexa Fluor 488 (Cat A0423, Beyotime) or Alexa Fluor 647 (Cat A0473, Beyotime) and visualized with a confocal laser scanning microscope (Leica TCS SP8).

The mIHC staining was conducted using an Opal Polaris 7-Color IHC Kit (Akoya Biosciences) following the provided instructions. Briefly, specimens were retrieved in AR6 buffer for 30 minutes, followed by a blocking step of one-hour, and subsequent incubation with primary antibodies, Opal Polymer HRP Ms+Rb, and Opal work solution in sequence. This process was repeated until all antigens were labeled. The slides were then scanned using an automated imaging system (PhenoImager™ HT, Akoya Biosciences). The three staining panels contained the following antibodies: Panel 1, CD3 (Cat ab16669, Abcam), CD8 (Cat 70306S, Cell Signaling Technology), CD56 (Cat 3576S, Cell Signaling Technology), FOXP3 (Cat BX50188, Biolynx), PD-1 (Cat 86163T, Cell Signaling Technology), and Granzyme B (GZMB, Cat 46890T, Cell Signaling Technology); Panel 2, PAN-CK (Cat ab7753, Abcam), CD68 (Cat ab201340, Abcam), CD66B (Cat ab300122, Abcam), CD11C (Cat 45581T, Cell

Signaling Technology), CD20 (Cat ab78237, Abcam), and CD138 (Cat ab128936, Abcam); Panel 3, CD68 (Cat ab201340, Abcam), CD66B (Cat ab300122, Abcam), CD138 (Cat ab128936, Abcam), and SIGLEC-8 (Cat ab305297, Abcam). Cell types were defined as follows: T cells (CD3^{+}CD56^{-}), CD8^{+} T cells (CD3^{+}CD8^{+}CD56^{-}), NK cells (CD3^{-}CD56^{+}), macrophages (CD68^{+}), neutrophils (CD66B^{+}), dendritic cells (CD11C^{+}CD68^{-}CD66B^{-}Pan-CK^{-}CD20^{-}), B cells (CD20^{+}), plasma cells (CD138^{+}CD68^{-}CD66B^{-}CD11C^{-}Pan-CK^{-}CD20^{-}), activated cytotoxic CD8^{+} T cells (CD3^{+}CD8^{+}CD56^{-}GZMB^{+}), exhausted CD8^{+} T cells (CD3^{+}CD8^{+}CD56^{-}PD-1^{+}), and Treg cells (CD3^{+}CD8^{-}CD56^{-}Foxp3^{+}).

Neutrophil extracellular trap (NET) formation assay

Neutrophils, classified as either Siglec-F^{+} or Siglec-F^{-}, were isolated from the tumor tissues of orthotopic KPC mice and seeded at a density of 1X10^{5} cells per well in a 24-well plate containing RPMI 1640 medium supplemented with 10% FBS and 1% penicillin/streptomycin. After a 24-hour culture, 200 nM SYTOX Green (Thermofisher) in HBSS was carefully added to each well to avoid disturbing the formation of NETs. Following a 15-minute incubation, the plate was imaged using a confocal laser scanning microscope (Leica TCS SP8). Five fields of view at 10× magnification were captured for each well. The analysis was performed using ImageJ software, and NET extension was quantified as the percentage of the total area occupied by the SYTOX Green-positive region.

Single cell sample preparation

Minced tissues (2-4 mm^3) were digested for 30 minutes at 37 °C in an enzymatic solution containing collagenase type IV and DNase I (Sigma). The single-cell suspensions were filtered through 70-μm and 30-μm cell strainers (Miltenyi Biotech) and treated with Red Blood Cell Lysis Solution (Miltenyi Biotech). Cell viability was assessed using Countstar Rigel (Alit Biotech), and dead cell removal was carried out depending on the viability using the Dead Cell Removal Kit (Miltenyi Biotech). Finally, the cells were re-suspended in 1X PBS (Invitrogen) supplemented with 0.04% BSA at a final concentration of 700-1200 cells/μl, and subsequently processed with the 10X Chromium Single Cell 3' Kit (v3.1) according to the manufacturer's instructions at Novogene Bioinformatics Technology Co., Ltd (Tianjin, China).

Single-cell RNA sequencing (scRNA-seq)

The cell suspension was loaded into a 10X Chromium Chip (v3.1) and barcoded using a 10X Chromium Controller. RNA from the barcoded cells was subsequently reverse-transcribed, amplified, and prepared into sequencing libraries with 10X Library Construction Kit (v3.1), following the manufacturer's instructions. Sequencing was conducted with an Illumina NovaSeq, generating 150-bp paired-end reads at Novogene Bioinformatics Technology Co., Ltd (Tianjin, China).

Bulk RNA sequencing

RNA was extracted from Siglec-F^+ and Siglec-F^- neutrophils isolated from orthotopic

tumors or BMDMs exposed to IL-34 and/or PGE_2 using the MolPure Cell/Tissue Total RNA Kit (Yeasen, China). Prior to analysis, the RNA samples underwent quality assessment. Following this assessment, mRNA was enriched using magnetic oligo (dT) beads to construct a sequencing library. Adapter ligation and size selection were subsequently performed, followed by the amplification of the cDNA library. Sequencing was conducted on the Novaseq 6000 platform (Illumina, San Diego, CA, USA).

Processing of scRNA-seq data

FASTQ files were processed using Cell Ranger (v7.2.0, 10x Genomics) with default parameters and the mm10 mouse reference genome (version 2020-A, 10x Genomics). Subsequent analyses were conducted in R (v4.4.1) utilizing Seurat (v5.3.0)(1). A Seurat object was generated, and the percentage of mitochondrial gene expression was calculated for quality control purposes. Cells with fewer than 1,000 unique molecular identifiers (UMI) counts, fewer than 300 expressed genes, or greater than 20% mitochondrial gene content were excluded from the analysis.

Seurat objects derived from sorted F4/80$^+$ cells were merged. The data were normalized, and variable features were selected using SCTransform(2, 3). Principal component analysis (PCA) was performed using RunPCA, retaining the first 100 principal components. Batch effects were corrected using Harmony(4), which was implemented via Seurat's integration function, with the corrected embeddings saved as 'harmony'. A shared nearest neighbor (SNN) graph was constructed using FindNeighbors based on

the first 20 harmony dimensions. Clustering was executed using the Louvain algorithm in FindClusters (resolution=1.0). Cluster-specific marker genes were identified using FindAllMarkers (only.pos=TRUE, min.pct=0.1, FDR<0.01). Cells expressing conflicting lineage markers were classified as doublets and subsequently removed. Clusters were merged based on their top marker genes, and distinctive features were recalculated using FindAllMarkers with the same parameters. Uniform manifold approximation and projection (UMAP)(5) was performed using RunUMAP for two-dimensional visualization.

The scRNA-seq data of monocytes derived from the blood of orthotopic KPC mice were obtained from the GEO database (accession code: GSE217846) and subsequently merged with Seurat objects from live cells isolated from the tumor tissues of orthotopic KPC mice. The data were normalized using the NormalizeData function, scaled with the ScaleData function, and variable features were selected using FindVariableFeatures. PCA was performed with the RunPCA function, utilizing 100 principal components. Batch effects were corrected using IntegrateLayers with HarmonyIntegration. A SNN graph was constructed using FindNeighbors, followed by clustering with FindClusters (default parameters unless otherwise specified). Finally, UMAP was applied for two-dimensional visualization using RunUMAP.

A public scRNA-seq dataset (accession code: PRJNA978570) concerning healthy pancreas and acute pancreatitis in mice was retrieved from the European Nucleotide Archive (ENA) browser (https://www.ebi.ac.uk/ena/browser/view/PRJNA978570). The data preprocessing adhered to the protocols outlined previously.

Identification of main cell types

After identifying marker genes using the FindAllMarkers function, cells derived from the blood and tumor tissues of orthotopic KPC mice were categorized into eight main types: ductal cells (*Krt18, Krt8, Epcam, Cdkn2a, Clu*), CAFs (*Col1a1, Col1a2, Dcn, Col5a2, Bgn*), DCs (*Flt3, Grk3, Htr7*), mast cells (*Kit, Tpsb2, Cpa3, Hs6st2, Mcpt4*), monocytes/macrophages (Monos/Mphs) (*Cd14, Ly6c2, Cxc3r1, Cd68, Adgre1, Fcgr1, C1qa, C1qb*), neutrophils (*Csf3r, S100a9, S100a8, Acod1*), B cells (*Cd79a, Cd19, Cd79b, Ms4a1*), and T/NK cells (*Cd3d, Cd3e, Nkg7, Klrd1, Cd4, Cd8a*). Furthermore, DCs were subclassified into cDC1 (*H2-Oa, H2-Dmb2*) and cDC2 (*Ccr7, Fscn1*) based on their distinct signatures.

For the dataset of healthy pancreas and acute pancreatitis in mice, 14 distinct cell types were identified: acinar cells, early acinar-to-ductal metaplasia (ADM–early), late acinar-to-ductal metaplasia (ADM–late), ductal cells, endocrine cells, fibroblasts, endothelial cells, pericytes, erythrocytes, T/NK cells, B cells, DCs, neutrophils, and Monos/Mphs, as referenced in Aney et al.(6) along with established marker genes. Subsequently, Monos/Mphs and neutrophils were extracted for further analysis.

Identification of monocyte, macrophage, and neutrophil subtypes

The scRNA-seq data of F4/80$^+$ cells, sorted from tumor tissues of orthotopic KPC mice, were pre-processed. Following dimension reduction using UMAP and the construction of mutual nearest neighbors (MNN), F4/80$^+$ cells were categorized into seven subtypes:

MM1/*Sdc1*$^{+}$ mphs (*Sdc1, Saa3, Ccl2, Cxcl3, Met*), MM2/*Cxcl9*$^{+}$ mphs (*Cxcl9, Slamf8, Serpina3g, Mmp25, Stat1*), MM3/*Plac8*$^{+}$mphs (*Ace, Ly6c2, Plac8, Hp, Sell*), MM4/*Mki67*$^{+}$ mphs (*Pclaf, Top2a, Cdk1, Mki67, Ube2c*), MM5/*Mrc1*$^{+}$mphs (*Stab1, Pdgfc, Itga6, Mrc1, Wwp1*), MM6/*Il1b*$^{+}$ mphs (*Il1b, Clec4e, Adora2a, Atp2b4, Tnfsf9*), and DCs (*Ccl17, H2-Oa, Cd209a, H2-DMb2, Flt3*).

For the reclustering of monocytes and macrophages derived from blood and tumor tissues of orthotopic KPC mice, cells annotated as monocytes and macrophages were isolated, and pre-processing steps were conducted. After dimension reduction with UMAP and MNN construction, eight subsets were identified, which included the six macrophage subtypes mentioned above, along with two additional populations defined as *Apoe*$^{+}$ Mphs (*Apoe, Apol7c, Mmp13*) and *Fgfr2*$^{+}$ Mphs (*Fgfr2, Pde4c, Trim69*). Notably, the monocytes and the *Plac8*$^{+}$ macrophage subset were merged into *Plac8*$^{+}$ Monos/Mphs due to their similar transcriptional profiles (*Ace, Ly6c2, Plac8, Hp, Sell*). Cell communication analysis was performed using CellChat (v2.1.0)(7, 8).

Neutrophils were classified into four subtypes: MN1 (*Ltc4s, Siglecf, Ptgs1, Cysltr1, Scimp*), MN2 (*Hspa1b, Hspa1a, Hilpda, Inhba, Hsp90aa1*), MN3 (*Ifit3, Ifit1, Gbp5, Lrg1, Rsad2, Gbp2, Irf7*), and MN4 (*S100a8, S100a9, Rps26, Rps12, Rpl23*).

Monos/mphs and neutrophils extracted from the dataset concerning healthy pancreas and acute pancreatitis in mice were mapped to our in-house macrophage and neutrophil datasets using Seurat. Briefly, anchor identification and label prediction were performed based on SCT normalization and PCA reduction. The query data were projected onto the UMAP visualization defined by the reference, and the merged cell

embeddings were visualized using the reference-defined UMAP projection.

Identification of CD8$^+$ T cell subsets

Tumor-infiltrating CD8$^+$ T cells were isolated from orthotopic KPC mice that received adoptive transfers of either CD138$^+$ or CD138$^-$ TAMs, as well as from control and Sdc1-cKO mice bearing orthotopic tumors. These cells were subsequently analyzed using scRNA-seq. The data underwent preprocessing and analysis in accordance with established protocols. Seven distinct CD8$^+$ T cell subsets were identified based on the expression levels of genes related to T cell proliferation, activation, and exhaustion. These subsets included naive-like, early-activation, effector-memory (T_{EM}), precursor-exhausted (Tpex), intermediate exhausted (Intermediate Tex), terminally-exhausted (Tex), and proliferating CD8$^+$ T cells.

Cell fate probability calculation

Pseudotime analysis was conducted using Monocle3 (v1.3.4)(9–11). The Seurat object was converted to an AnnData format utilizing SeuratDisk (v0.0.0.9020) and subsequently imported into Python (v3.11.7). Transition matrices were generated with CellRank (v2.0.4)(12) employing PseudotimeKernel (based on Monocle3 pseudotime) and ConnectivityKernel (based on transcriptomic similarity), which were combined into a single kernel with weights of 0.8 and 0.2, respectively. Generalized Perron Cluster Cluster Analysis (GPCCA)(13) was applied to identify macrostates of cellular dynamics. Terminal states were inferred, and fate probabilities were computed for the

lineage leading to *Sdc1*$^{+}$ TAMs. Driver genes were identified based on their expression correlation with fate probabilities toward the *Sdc1*$^{+}$ TAM terminal state. The top 10 and bottom 10 driver genes were selected and visualized in a heatmap using the ComplexHeatmap package (v2.22.0)(14) in R.

Survival analysis based on RNA sequencing data

Specific marker genes for each macrophage and neutrophil subset were identified based on the criteria of an average log2fold change (FC) greater than 1, an adjusted p value of less than 0.01, and expression present in over 30% of cells. Mouse gene symbols were converted to their human orthologs utilizing ENSEMBL v109, excluding mitochondrial genes from the analysis. Additionally, canonical marker genes were manually incorporated for each subset, including *CD68, CD14,* and *ADGRE1* for macrophage subsets, as well as *CSF3R* for neutrophil subsets. For the MM4 subset, only the top 80 most variable marker genes were retained, as its transcriptional profile contained an excessive number of marker genes meeting the established criteria. Subsequently, the bulk RNA-seq dataset from patients with PDAC (TCGA-PAAD) was obtained from the GDC Data Portal (https://portal.gdc.cancer.gov/projects/TCGA-PAAD). Gene Set Variation Analysis (GSVA) enrichment scores for the marker genes of each subset were computed using the GSVA package (v2.0.7)(15) with the gsvaParam method, based on the transcripts per million (TPM)-normalized transcriptional profile from the TCGA dataset. Patients were stratified into high- and low-score groups according to the median score of each subset. Kaplan-Meier survival

analysis was conducted to estimate overall survival, and the statistical significance of differences between groups was determined using the log-rank test implemented in the survminer (v0.5.0) and survival (v3.8.3) R packages. Survival curves, along with risk tables indicating the number of patients at risk, were generated for visualization.

Processing of bulk RNA-seq data

Transcriptome indices were generated utilizing the GRCm39 mouse reference genome and GENCODE M29 transcript annotations. Sequencing data were quantified with Salmon (v1.9.0)(16). Read count matrices were imported and aggregated through the tximport function from the tximport package (v1.30.0) in R (v4.4.1). Differential expression analysis was performed with DESeq2 (v1.42.0)(17) to conduct pairwise comparisons among the groups: Control, IL-34, PGE_2, and IL-34 plus PGE_2, applying thresholds of log2FC>1 and adjusted p-value<0.05. To investigate the synergistic effects of IL-34 and PGE_2, genes differentially expressed in all three pairwise comparisons, including IL-34 plus PGE_2 vs. PGE_2, IL-34 plus PGE_2 vs. IL-34, and IL-34 plus PGE_2 vs. Control, were identified as potential signature genes. A heatmap of these signature genes was generated using the ComplexHeatmap package (v2.22.0)(14), based on variance-stabilized counts obtained from DESeq2's variance stabilizing transformation function.

Processing of Smart-seq data

The transcriptional profiles of $CD138^+$ and $CD138^-$ macrophages derived from

orthotopic tumors were analyzed using Smart-seq. Low-input libraries were constructed with the SMART-Seq v4 Ultra Low Input RNA Kit (Clontech), followed by cDNA synthesis, purification, and size selection. After adapter ligation and quality control using the Agilent 2100 Bioanalyzer, the libraries were sequenced on the Illumina platform employing a 2×150 bp paired-end protocol.

Raw FASTQ reads were aligned and quantified against the GRCm39 reference genome from ENSEMBL (https://www.ensembl.org) utilizing STAR software (v2.7.11b)(18). Transcript expression levels were normalized as TPM and log2-transformed for subsequent analyses. Differential expression analysis was performed using the limma package (v3.62.2)(19).

Gene set enrichment analysis (GSEA)

GSEA (v4.3.2)(20, 21) was conducted to identify functional alterations in the MM1/*Sdc1*$^+$ TAM subset, utilizing Gene Ontology (GO) gene sets from the mouse collection of the Molecular Signatures Database (MSigDB)(22). The input data were derived from the normalized count matrix of the Seurat object, which was generated from sorted F4/80$^+$ cells.

GSEA was applied to genes that were synergistically regulated by IL-34 and PGE_2 treatment, and normalized enrichment scores (NES) were calculated for each monocyte and macrophage subtype. Furthermore, GSEA was performed on driver genes inferred for the *Sdc1*$^+$ TAM lineage using the GSEA function from the clusterProfiler package (v4.12.0)(23), with results ranked according to NES.

For CD8$^+$ T cells within the orthotopic tumors derived from mice that underwent adoptive transfer, as well as from control and Sdc1-cKO mice, GSEA was conducted referencing to the M2, M5, and Mh gene sets from the MSigDB database(22).

The IFNγ production score was calculated using the AddModuleScore function of Seurat, with the gene set comprising *Ifng*, *Gzmb*, *Gzma*, *Gzmk*, and *Prf1*(24).

Kyoto Encyclopedia of Genes and Genomes (KEGG) enrichment analysis

Enrichment analysis of the KEGG database (Release 106.0) was performed using a custom database generated via the create_kegg_db function from the createKEGGdb package (v0.0.3). For bulk RNA-seq data, genes exhibiting |log2FC| > 1 and an adjusted p-value<0.05 were classified as either upregulated or downregulated. A hypergeometric test was utilized to identify enriched KEGG pathways for both upregulated and downregulated genes, employing the enrichKEGG function in clusterProfiler with default parameters. In the context of single-cell RNA-seq, features with log2FC>1, Pct.1>0.2, and an adjusted p-value<0.05 were designated as marker genes of *Sdc1*$^+$ TAMs and served as the input data for KEGG enrichment analysis. In the analysis of Smart-seq data, genes with a log2FC greater than 1 and an adjusted p-value of less than 0.2 were classified as upregulated, serving as the input data for KEGG enrichment analysis. Only terms with an adjusted p-value of less than 0.2 were retained for further interpretation.

mIHC analysis

All mIHC data were analyzed using PanoScore software (Panovue, Beijing, China). In Panel 1 and 2, cell types were identified based on the specific markers outlined previously, and the cell numbers, proportions and tissue areas were subsequently calculated. In Panel 3, TAMs were identified as $CD68^+$ and neutrophils as $CD66B^+$. Subtypes were delineated based on the upper quantile expression of CD138 in TAMs and the median expression of SIGLEC-8 in neutrophils. We identified SIGLEC-8^+ neutrophils in proximity to either $CD138^+$ or $CD138^-$ TAMs within a maximum distance of 20μm. The effective score and effective percent, which estimate the relative spatial positioning as previously described(25), were calculated for $CD138^+$ and $CD138^-$ TAMs in Cohort 2 and Cohort 3. In brief, the effective score denotes the average number of SIGLEC-8^+ neutrophils paired with $CD138^+$ or $CD138^-$ TAMs, while the effective percent represents the proportion of $CD138^+$ or $CD138^-$ TAMs that are paired with at least one SIGLEC-8^+ neutrophil among all $CD138^+$ or $CD138^-$ TAMs.

Supplemental references

1. Hao Y, et al. Integrated analysis of multimodal single-cell data. *Cell*. 2021;184(13):3573-3587.e29.

2. Hafemeister C, Satija R. Normalization and variance stabilization of single-cell RNA-seq data using regularized negative binomial regression. *Genome Biol*. 2019;20(1):1–15.

3. Choudhary S, Satija R. Comparison and evaluation of statistical error models for scRNA-seq. *Genome Biol*. 2022;23(1):1–20.

4. Korsunsky I, et al. Fast, sensitive and accurate integration of single-cell data with Harmony. *Nat Methods*. 2019;16(12):1289–1296.

5. Becht E, et al. Dimensionality reduction for visualizing single-cell data using UMAP. *Nat Biotechnol*. 2019;37:38–44.

6. Aney KJ, et al. Novel Approach for Pancreas Transcriptomics Reveals the Cellular Landscape in Homeostasis and Acute Pancreatitis. *Gastroenterology*. 2024;166(6):1100–1113.

7. Jin S, Plikus M V, Nie Q. CellChat for systematic analysis of cell-cell communication from single-cell transcriptomics. *Nat Protoc*. 2025;20(1):180–219.

8. Jin S, et al. Inference and analysis of cell-cell communication using CellChat. *Nat Commun*. 2021;12(1):1088.

9. Trapnell C, et al. The dynamics and regulators of cell fate decisions are revealed by pseudotemporal ordering of single cells. *Nat Biotechnol*. 2014;32(4):381–386.

10. Qiu X, et al. Reversed graph embedding resolves complex single-cell trajectories.

Nat Methods. 2017;14(10):979–982.

11. Cao J, et al. The single-cell transcriptional landscape of mammalian organogenesis. *Nature*. 2019;566(7745):496–502.

12. Weiler P, et al. CellRank 2: unified fate mapping in multiview single-cell data. *Nat Methods*. 2024;21(7):1196–1205.

13. Reuter B, et al. Generalized Markov State Modeling Method for Nonequilibrium Biomolecular Dynamics: Exemplified on Amyloid β Conformational Dynamics Driven by an Oscillating Electric Field. *J Chem Theory Comput*. 2018;14(7):3579–3594.

14. Gu Z. Complex heatmap visualization. *iMeta*. 2022;1(3):e43.

15. Hänzelmann S, Castelo R, Guinney J. GSVA: gene set variation analysis for microarray and RNA-seq data. *BMC Bioinformatics*. 2013;14:7.

16. Patro R, et al. Salmon provides fast and bias-aware quantification of transcript expression. *Nat Methods*. 2017;14(4):417–419.

17. Love MI, Huber W, Anders S. Moderated estimation of fold change and dispersion for RNA-seq data with DESeq2. *Genome Biol*. 2014;15(12):550.

18. Dobin A, et al. STAR: ultrafast universal RNA-seq aligner. *Bioinformatics*. 2013;29(1):15–21.

19. Ritchie ME, et al. limma powers differential expression analyses for RNA-sequencing and microarray studies. *Nucleic Acids Res*. 2015;43(7):e47.

20. Subramanian A, et al. Gene set enrichment analysis: A knowledge-based approach for interpreting genome-wide expression profiles. *Proc Natl Acad Sci U S A*. 2005;102(43):15545–15550.

21. Mootha VK, et al. PGC-1alpha-responsive genes involved in oxidative phosphorylation are coordinately downregulated in human diabetes. *Nat Genet*. 2003;34(3):267–273.

22. Liberzon A, et al. The Molecular Signatures Database Hallmark Gene Set Collection. *Cell Syst*. 2015;1(6):417–425.

23. Wu T, et al. clusterProfiler 4.0: A universal enrichment tool for interpreting omics data. *Innovation*. 2021;2(3):100141.

24. Liu Y, et al. Syndecan-1 inhibition promotes antitumor immune response and facilitates the efficacy of anti-PD1 checkpoint immunotherapy. *Sci Adv*. 2024;10(37):eadi7764.

25. Jia K, et al. Multiplex immunohistochemistry defines the tumor immune microenvironment and immunotherapeutic outcome in CLDN18.2-positive gastric cancer. *BMC Med*. 2022;20(1):223.

26. Han S, et al. A Novel Subset of Anti-Inflammatory CD138(+) Macrophages Is Deficient in Mice with Experimental Lupus. *J Immunol*. 2017;199(4):1261–1274.

Supplemental Figures

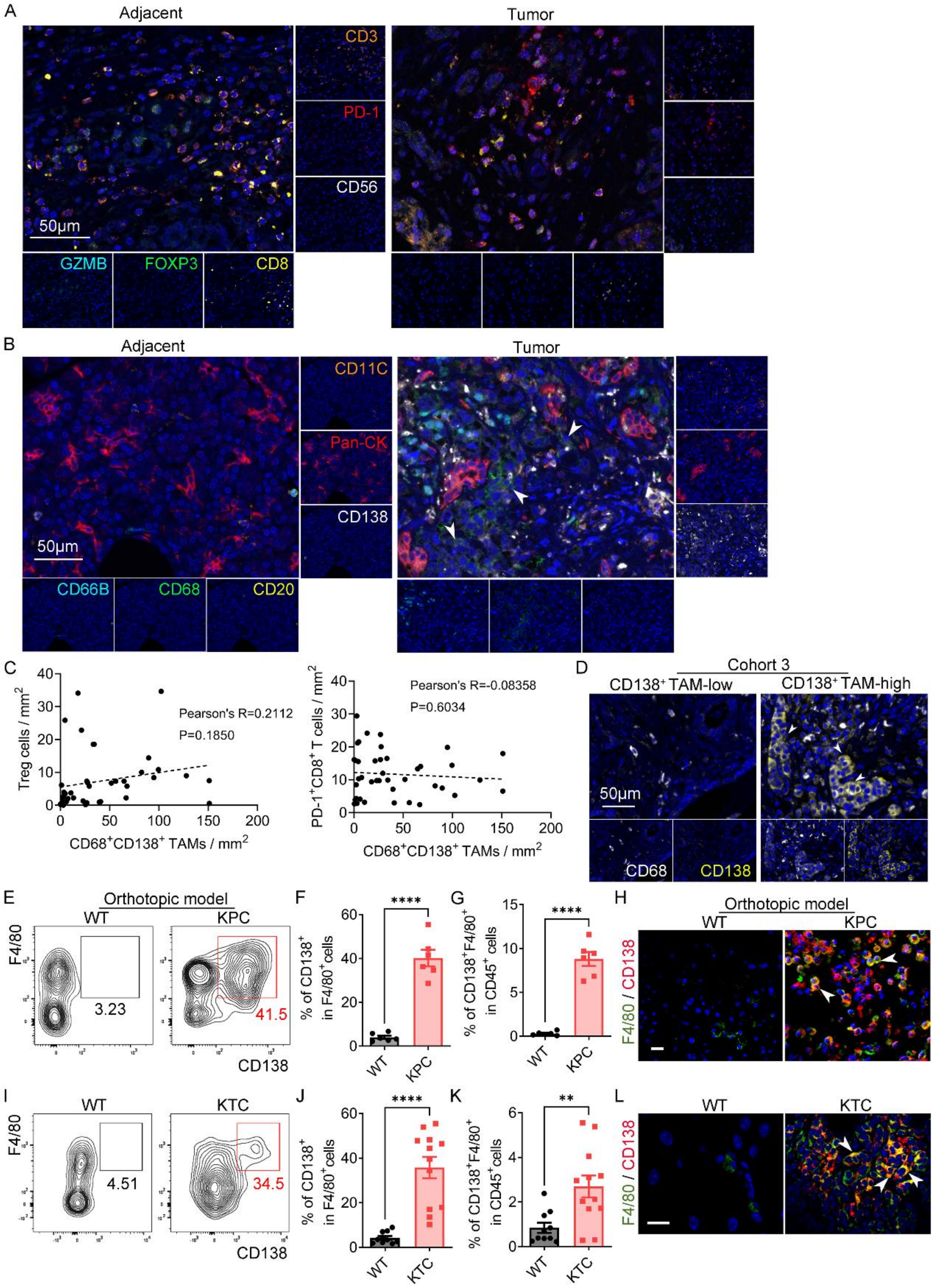

Supplemental Figure 1. The expansion of a CD138^{+} TAM population is found in both PDAC patients and corresponding mouse models. (**A** and **B**) Representative mIHC images depicting the cell types illustrated in **Figure 1, A-C**, with CD138^{+}CD68^{+}

TAMs indicated by white arrows. (**C**) Correlation among the abundance of CD138$^+$ TAMs, Treg cells, and exhausted CD8$^+$ T cells in tumor tissues from PDAC patients in Cohort 2 (n=41). (**D**) Representative images displaying CD68$^+$CD138$^+$ macrophages (white arrows) in the CD138$^+$ TAM-low and CD138$^+$ TAM-high groups from PDAC patients in Cohort 3. (**E** and **I**) Flow cytometric plots depicting CD138$^+$F4/80$^+$ macrophages in the tumor tissues of orthotopic KPC (**E**) and KTC (**I**) mice. (**F** and **G**) Quantification of (**E**) (n=6 per group). (**H** and **L**) Immunofluorescence microscopy images of tumors from orthotopic KPC (**H**) and KTC mice (**L**), revealing the presence of CD138$^+$F4/80$^+$ macrophages (white arrows). Scale bar, 10μm. (**J** and **K**) Quantification of (**I**) (n=10-12 per group). **p<0.01 and ****p<0.0001 by unpaired t test (**F**, **G**, **J**, and **K**). Data represent mean ± SEM.

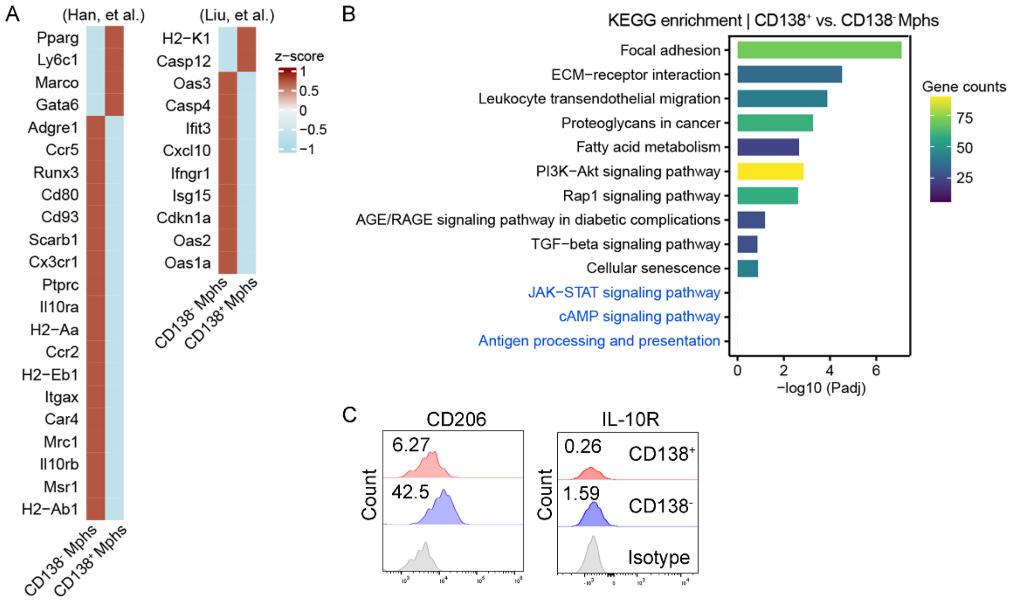

Supplemental Figure 2. CD138$^+$ TAMs do not exhibit anti-inflammatory properties in PDAC. (**A**) Heatmaps displaying the relative expression levels of signature genes associated with anti-inflammatory CD138$^+$ macrophages, along with

genes down-regulated by syndecan-1(24, 26), in both $CD138^+$ and $CD138^-$ macrophages derived from orthotopic tumors. (**B**) KEGG enrichment analysis of DEGs between $CD138^+$ and $CD138^-$ macrophages isolated from orthotopic tumors. Pathways previously identified as activated in anti-inflammatory $CD138^+$ macrophages, alongside those down-regulated by syndecan-1(blue), are additionally included in the bar plot (24, 26). (**C**) Flow cytometric images illustrating the frequencies of $CD206^+$ and IL-$10R^+$ cells in $CD138^-$ (blue line) and $CD138^+$ (red line) TAMs derived from PDAC patients in Cohort 1. The isotype control is depicted by the gray line.

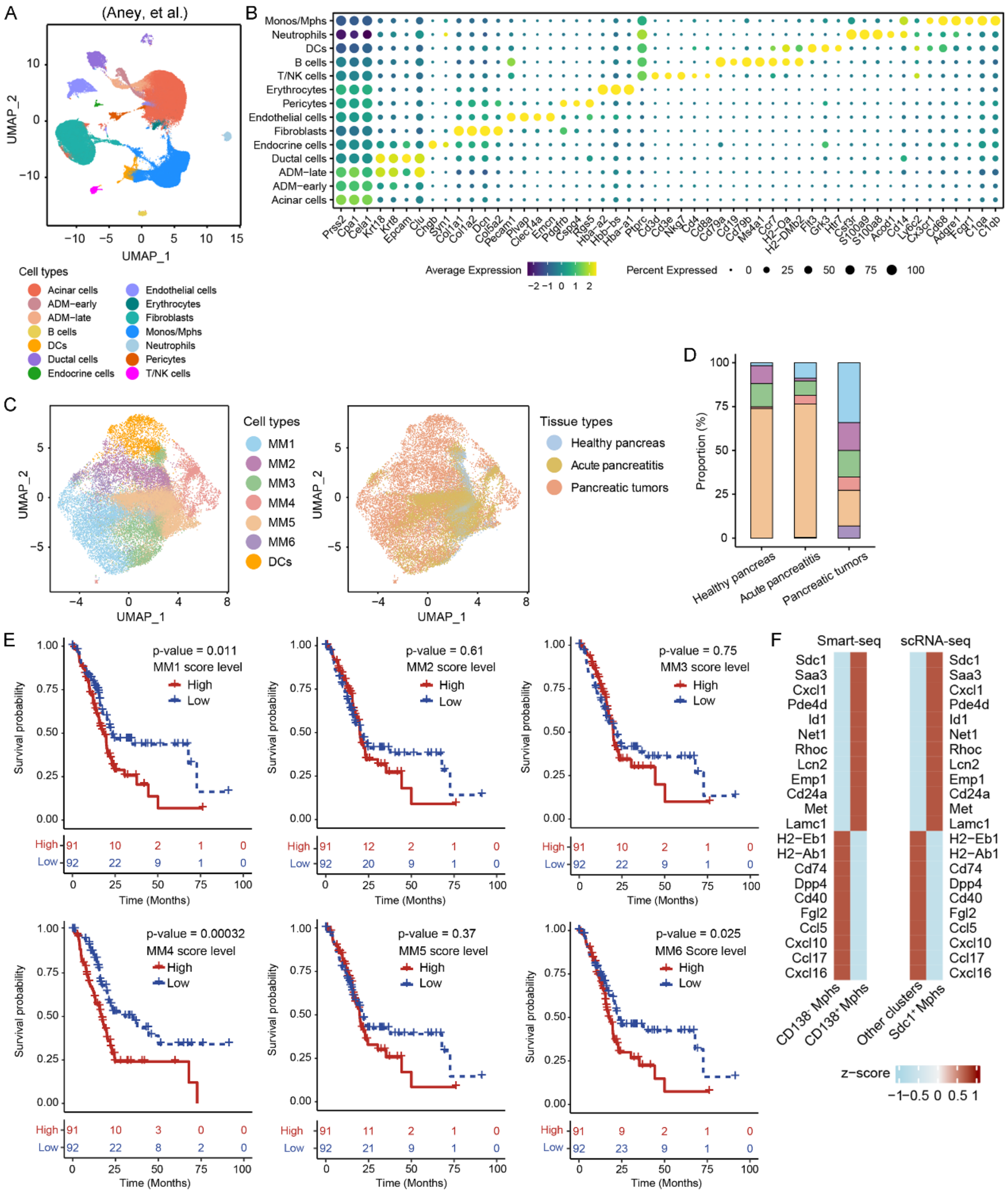

Supplemental Figure 3. A pro-inflammatory CD138^{+} macrophage subset is expanded and correlated with poor prognosis in PDAC. (**A**) UMAP plot displaying viable cells in the pancreas of both healthy and acute pancreatitis mice, with colors indicating distinct scRNA-seq clusters. (**B**) Bubble plot presenting selected cell type-specific markers across all clusters, with clustering performed as detailed in (**A**). The size of the dots represents the proportion of cells expressing a specific marker, while

the intensity of the color indicates the level of mean expression. Legends are provided below. (**C**) UMAP plots depicting macrophages in the pancreas of healthy and acute pancreatitis mice, as well as F4/80$^+$ cells sorted from orthotopic tumors, with colors representing scRNA-seq clusters (left) and the source of the cells (right). (**D**) Proportions of macrophage subsets in the pancreas of healthy and acute pancreatitis mice, as well as in orthotopic tumors. (**E**) Survival probabilities of PDAC patients classified into high- and low-score groups from the TCGA dataset, stratified by GSVA enrichment scores for each macrophage subset. (**F**) Heatmaps illustrating the scaled expression of common genes between DEGs of CD138$^+$ versus CD138$^-$ macrophages from orthotopic tumors and marker genes of *Sdc1*$^+$ macrophage subset (MM1).

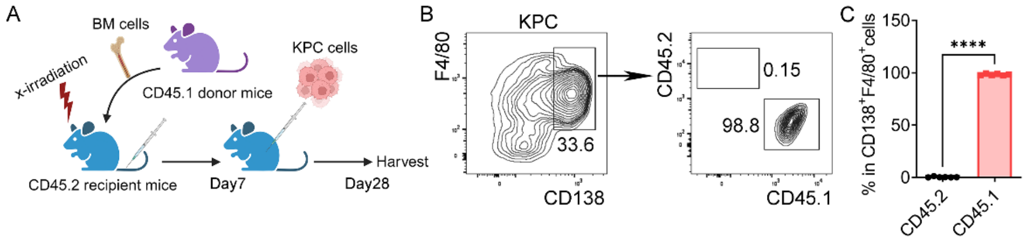

Supplemental Figure 4. CD138$^+$ TAMs originate from circulating monocytes. (**A**) Experimental approach employed to assess the origin of CD138$^+$ TAMs utilized a CD45.1/CD45.2 chimera mouse model. (**B**) Frequencies of CD45.1$^+$ and CD45.2$^+$ cells within CD138$^+$F4/80$^+$ macrophages in the tumor tissues of CD45.1/CD45.2 chimera mice bearing orthotopic KPC tumors. (**C**) Quantification of (**B**) (n=6 per group). ****$p<0.0001$ by unpaired t-test (**C**). Data represent mean ± SEM.

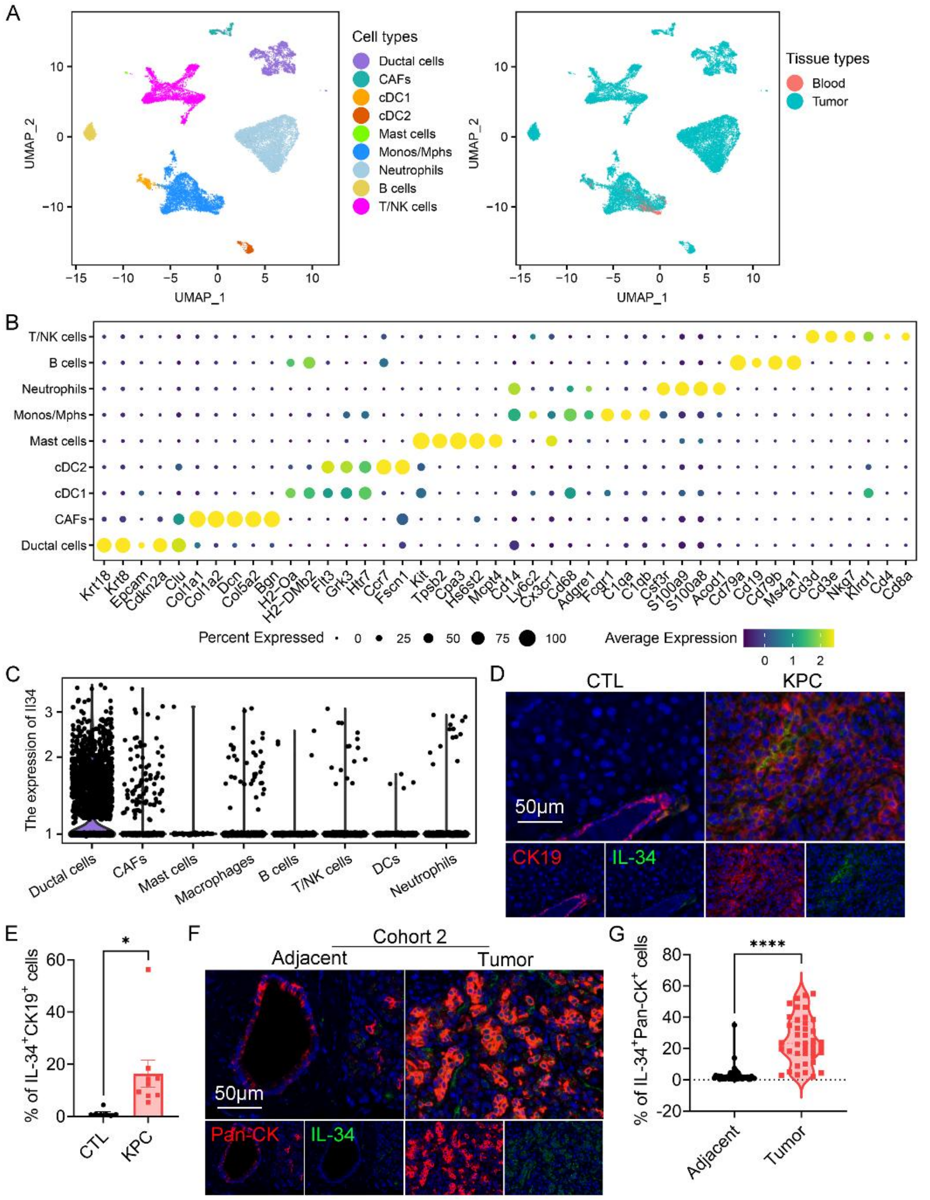

Supplemental Figure 5. The expression levels of IL-34 are elevated in pancreatic tumor cells. (**A**) UMAP plots illustrating viable cells in the peripheral blood and tumor tissues of orthotopic KPC mice, with colors indicating scRNA-seq clusters (left) and the source of the cells (right). (**B**) Bubble plot displaying selected cell type-specific markers across all clusters, with clustering performed as detailed in (**A**). (**C**) Expression levels of *Il34* across all clusters in tumor tissues depicted in (**A**). (**D**) mIHC images highlighting IL-34^{+}CK19^{+} ductal cells in tumor tissues from mice with orthotopic KPC tumors, as well as in the pancreas of healthy mice. CK19 is a well-known marker for

ductal cells in the pancreas of mice. (**E**) Quantification of (**D**), showcasing the percentages of IL-34$^+$CK19$^+$ cells within the total cells in each tissue section (n=7-9 per group). (**F**) Representative images illustrating IL-34 expression in ductal cells in paired adjacent benign and tumor tissues from PDAC patients in Cohort 2. Pan-CK serves as a well-established marker for ductal cells in PDAC patients. (**G**) Quantification of (**F**), illustrating the percentages of IL-34$^+$Pan-CK$^+$ cells within the total cells in each tissue core (n=41 per group). *$p<0.05$ and ****$p<0.0001$ by paired or unpaired t-tests (**E** and **G**). Data represent mean ± SEM.

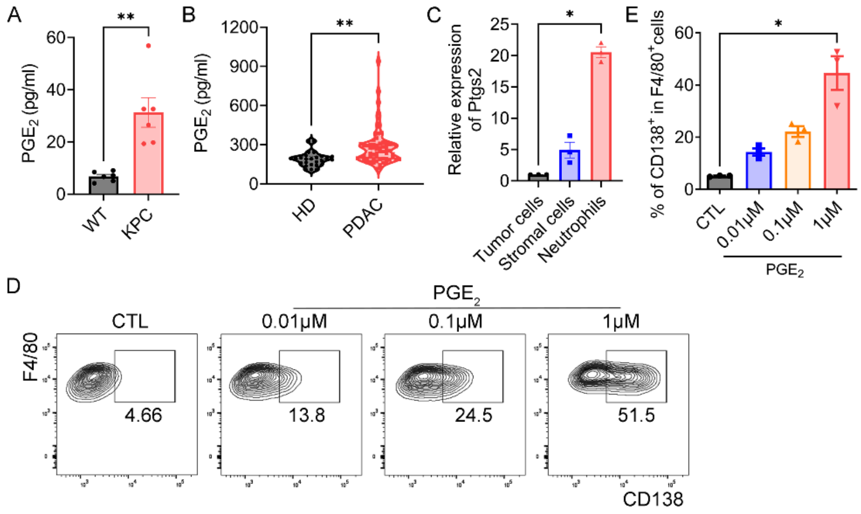

Supplemental Figure 6. PGE_2 induces the expression of syndecan-1 on BMDMs in a dose-dependent manner. (**A** and **B**) The serum levels of PGE_2 in wild-type mice and mice bearing orthotopic KPC tumors (**A**, n=6 per group), as well as in healthy donors (HD, n=26) and patients with PDAC (n=63) (**B**). (**C**) RT-PCR analysis measuring the expression levels of *Ptgs2* in GFP^+ tumor cells, GFP^-CD45^- stromal cells, and $CD45^+Ly6g^+GFP^-$ neutrophils sorted from GFP^+ orthotopic tumors ten days post-tumor implantation (n=3 per group). (**D**) Flow cytometric images illustrating the expression of CD138 in $F4/80^+$ macrophages derived from BMDM cultures exposed to varying concentrations of PGE_2. (**E**) Quantification of (**D**) (n=3 per group). *$p<0.05$ and **$p<0.01$ by unpaired t-test (**A** and **B**) and by Kruskal-Wallis test with Dunn's multiple comparison test (**C** and **E**). Data represent mean ± SEM.

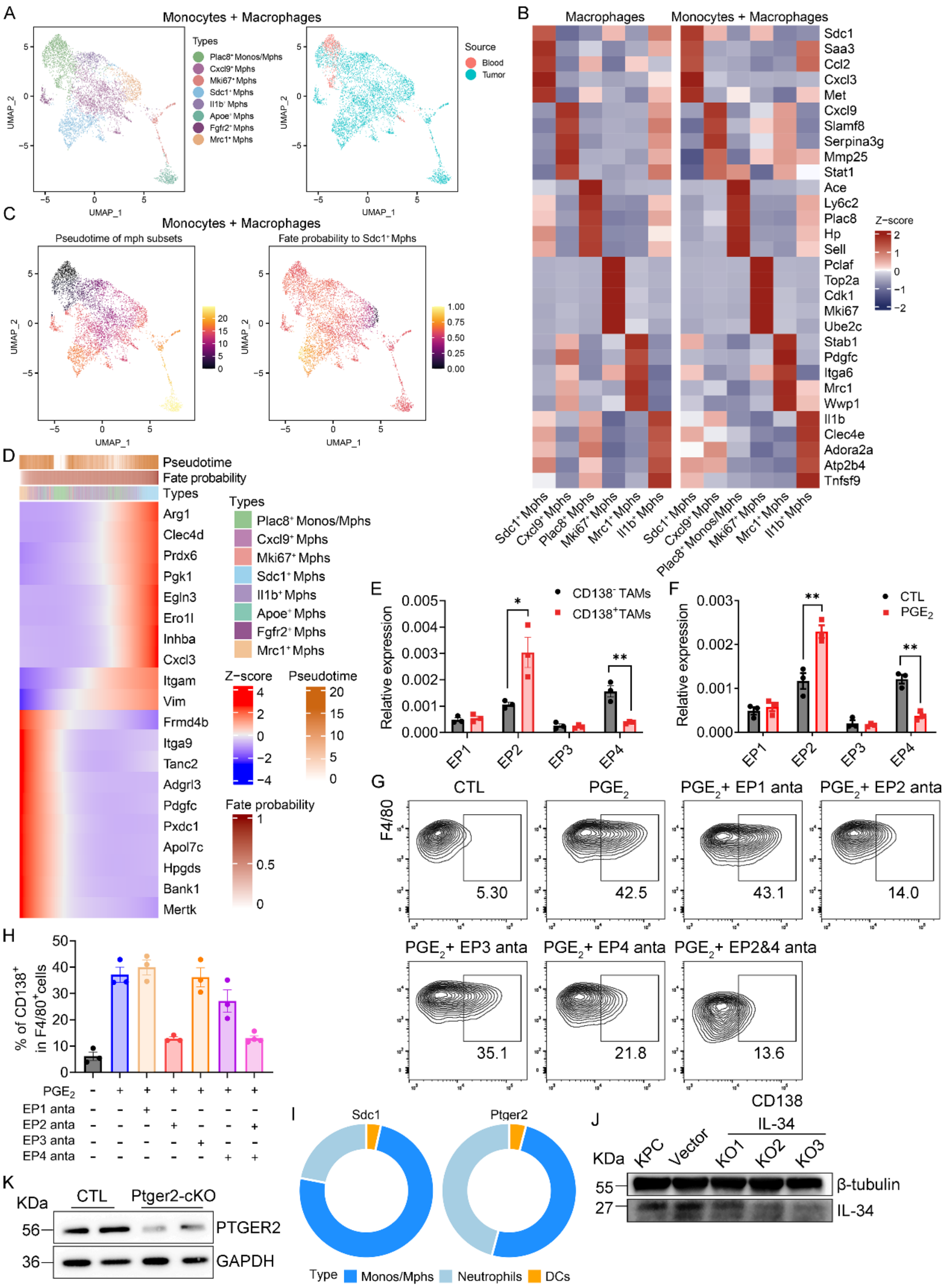

Supplemental Figure 7. The PGE2-EP2 signaling induces the expression of syndecan-1 on BMDMs. (A) UMAP plots showing the reclustering of monocytes/macrophages from the dataset presented in **Supplemental Figure 5A**. Colors denote the various monocyte/macrophage subclusters (left) and the source of the

cells (right). (**B**) Heatmaps illustrating the scaled expression of marker genes across monocyte/macrophage subsets, with reclustering executed as described in (**A**). (**C**) Pseudotime trajectory of single-cell transcriptomes of monocyte/macrophage subsets calculated using Monocle 3. The intensity of color indicates the levels of pseudotime (left). The fate probability of the monocyte-to-*Sdc1*$^+$ TAM trajectory is calculated by CellRank. The intensity of color represents levels of fate probability (right). Reclustering of monocytes/macrophages is performed as outlined in (**A**). (**D**) Heatmap of driver genes promoting the monocyte-to-*Sdc1*$^+$ TAM trajectory, as calculated by CellRank. (**E** and **F**) RT-PCR analysis measuring the expression levels of PGE_2 receptors in CD138$^-$F4/80$^+$ and CD138$^+$F4/80$^+$ macrophages isolated from tumor tissues of orthotopic KPC mice (**E**) and from BMDM cultures exposed to 1μM PGE_2 (**F**) (n=3 per group). (**G**) Flow cytometric images illustrating the expression of CD138 in F4/80$^+$ macrophages from BMDM cultures exposed to 1μM PGE_2, with or without EP1, EP2, EP3, and/or EP4 antagonists. (**H**) Quantification of (**G**) (n=3-4 per group). (**I**) Percentages of monocytes/macrophages, neutrophils, and DCs in *Sdc1*- and *Ptger2*-expressing myeloid cells derived from orthotopic tumors. Clustering is illustrated in **Supplemental Figure 5A**. (**J** and **K**) Western blot analysis evaluating the expression levels of IL-34 (27kDa) in KPC cells transfected with lentivirus (**J**), as well as PTGER2 (56kDa) in BMDMs from control and Ptger2-cKO mice (**K**). Equal loading is confirmed by quantifying β-tubulin (55kDa) and GAPDH (36kDa), respectively. $^*p<0.05$ and $^{**}p<0.01$ by unpaired t-test (**E** and **F**). Data represent mean ± SEM.

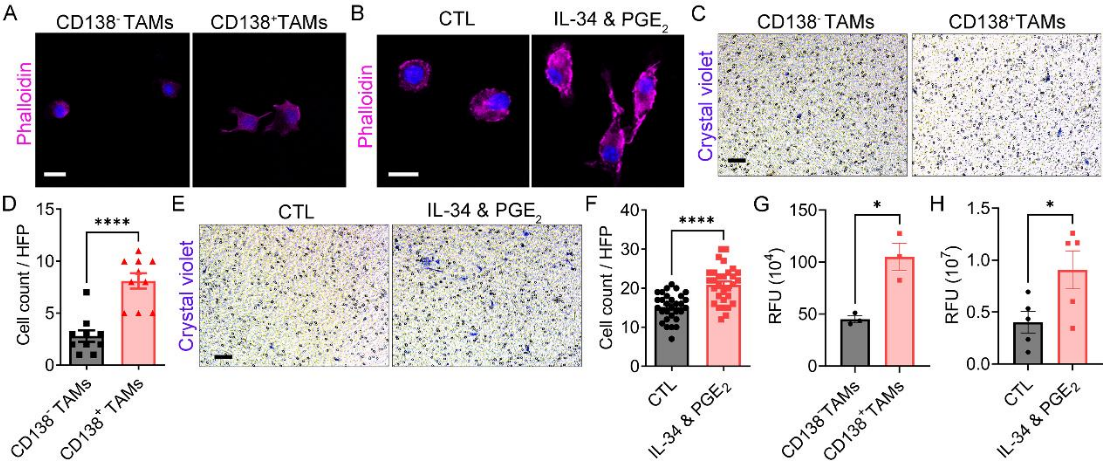

Supplemental Figure 8. CD138$^+$ macrophages co-induced by IL-34 and PGE_2 exhibit a phenotype comparable to that of CD138$^+$ TAMs from orthotopic tumors. (**A** and **B**) Phalloidin staining images showing the morphology of CD138$^-$ F4/80$^+$ and CD138$^+$ F4/80$^+$ macrophages isolated from the tumor tissues of orthotopic KPC mice (**A**), as well as from BMDM cultures exposed to IL-34 and PGE_2 (**B**). Scale bar, 10μm. (**C** and **E**) Representative crystal violet staining images illustrating the migration of CD138$^-$F4/80$^+$ and CD138$^+$F4/80$^+$ macrophages through the membrane of inserts in transwell culture systems. The cells were isolated from orthotopic tumors (**C**) and from BMDM cultures exposed to IL-34 and PGE_2 (**E**). Scale bar, 50μm. (**D** and **F**) Quantification of the number of cells depicted in (**C**) (**D**, n =10 per group) and (**E**) (**F**, n =30 per group). (**G** and **H**) Cell phagocytotic ability of CD138$^-$F4/80$^+$ and CD138$^+$F4/80$^+$ macrophages assessed using pHrodo™ Deep Red E. coli BioParticles™ Conjugate. The cells were isolated from orthotopic tumors (**G**) and from BMDM cultures exposed to IL-34 and PGE_2 (**H**). The graphs show the quantification of fluorescence signals (n=3 per group in (**G**) and n=5 per group in (**H**)). *$p<0.05$ and ****$p<0.0001$ by unpaired t-test (**D** and **F-H**). Data represent mean ± SEM.

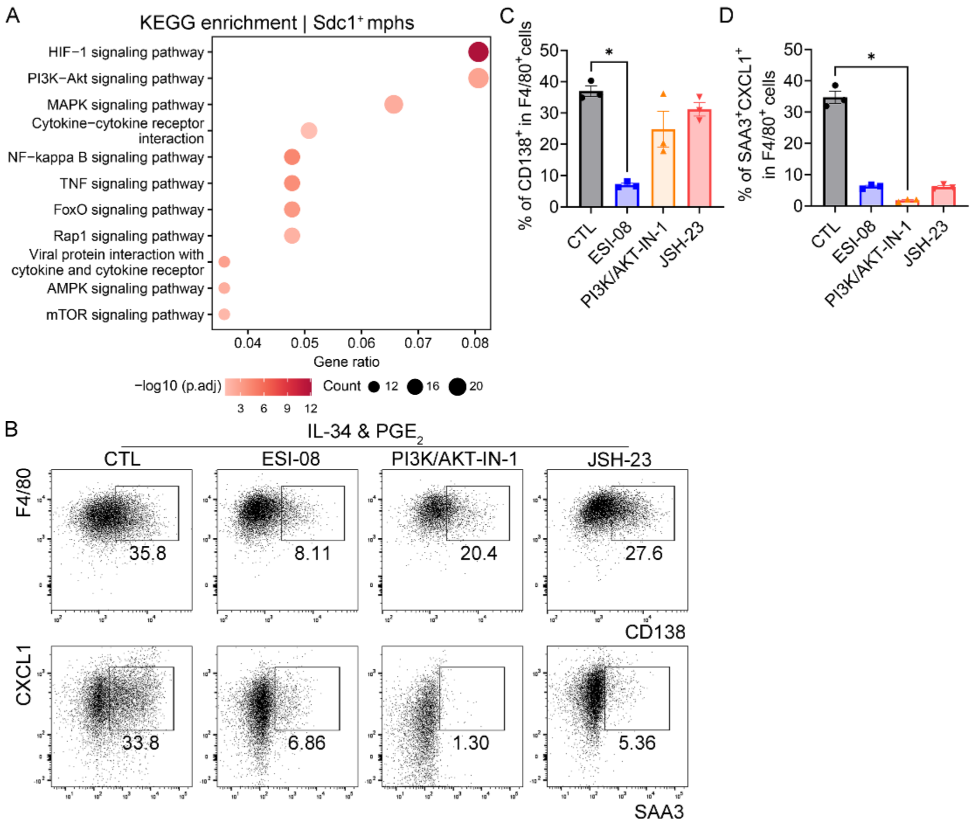

Supplemental Figure 9. IL-34 and PGE₂ collaboratively induce the differentiation of BMDMs into CD138⁺ macrophages through the activation of PI3K/Akt/NF-κB and EPAC/Rap1 signaling pathways. (**A**) KEGG enrichment analysis of signaling pathways related to environmental information processing on genes ranked by log_2FC between *Sdc1*⁺ macrophages and other monocyte/macrophage subsets. Reclustering of monocytes/macrophages is performed as illustrated in **Supplemental Figure 7A**. (**B**) Representative images depicting the expression of CD138, CXCL1, and SAA3 in F4/80⁺ macrophages from BMDM cultures exposed to IL-34 plus PGE_2, with or without the addition of ESI-08 (an EPAC/Rap1 pathway antagonist), PI3K/AKT-IN-1 (a PI3K-Akt pathway inhibitor), or JSH-23 (an NF-κB pathway inhibitor). (**C** and **D**) Quantification of (**B**) (n=3 per group). *p<0.05 by Kruskal-Wallis test with Dunn's

multiple comparison test (**C** and **D**). Data represent mean ± SEM.

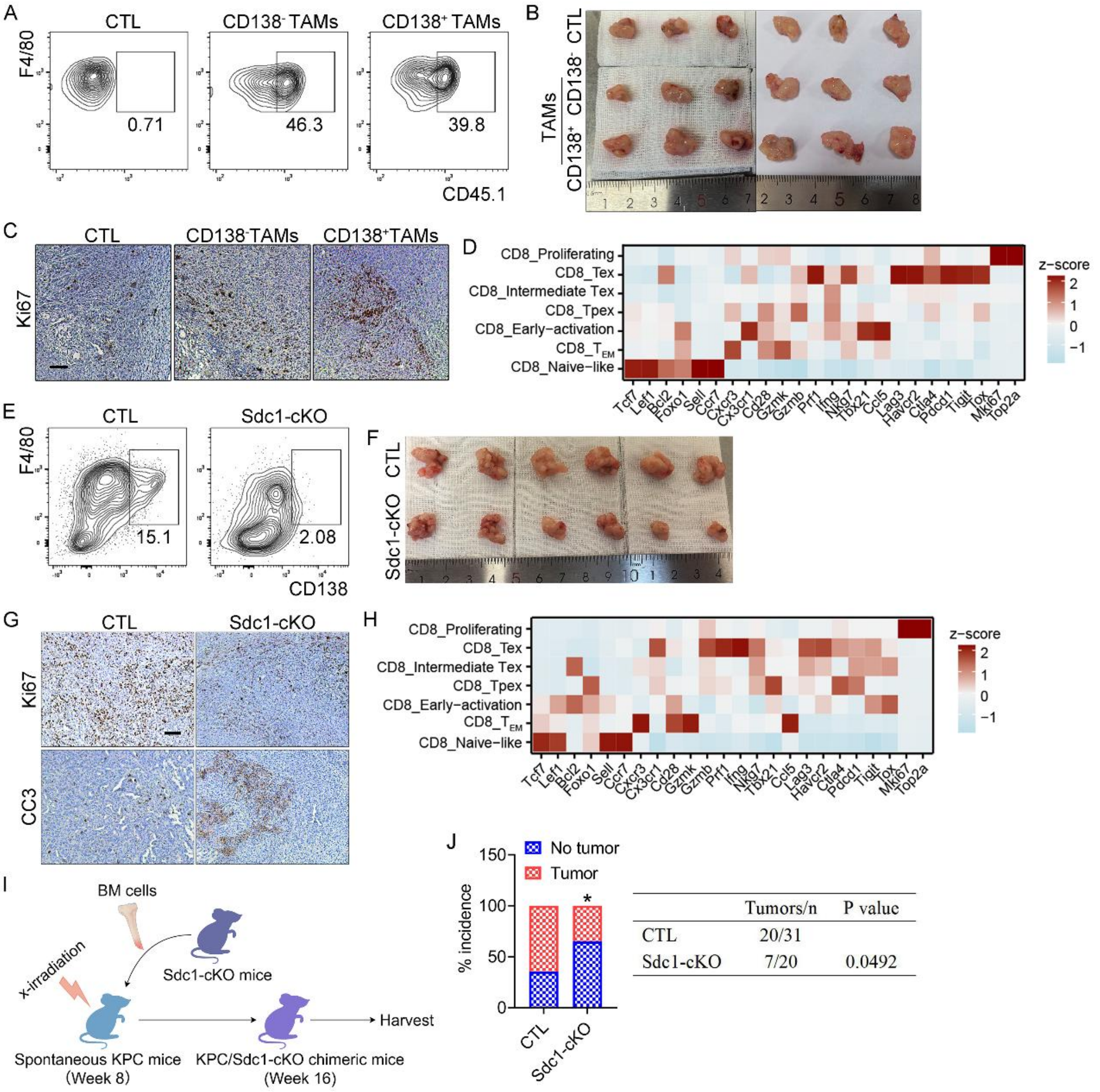

Supplemental Figure 10. CD138⁺ TAMs promote pancreatic tumor growth in murine models. (**A**) Flow cytometric images indicating the presence of CD45.1⁺ cells among F4/80⁺ macrophages within the tumor tissues of CD45.2⁺ mice following adoptive transfer, as illustrated in **Figure 4A**. (**B**) Images showing the harvested tumor tissues from mice post-adoptive transfer. (**C**) Immunohistochemical microscopy images depicting Ki67⁺ cells in tumor tissues from mice after adoptive transfer. Scale bar, 50μ m. (**D**) Heatmap illustrating the scaled expression of genes related to T cell proliferation,

activation, and exhaustion across tumor-infiltrating $CD8^+$ T cell subsets in mice that underwent adoptive transfer, with clustering performed as described in **Figure 4F**. (**E**) Flow cytometric analysis revealing the presence of $CD138^+F4/80^+$ TAMs in control and Sdc1-cKO mice with orthotopic KPC tumors. (**F**) Representative images showing the harvested tumor tissues from control and Sdc1-cKO mice bearing orthotopic tumors. (**G**) Immunohistochemical microscopy images displaying $Ki67^+$ cells and $CC3^+$ areas in tumor tissues of control and Sdc1-cKO mice with orthotopic tumors. Scale bar, 50μ m. (**H**) Heatmap illustrating the scaled expression of genes related to T cell proliferation, activation, and exhaustion across tumor-infiltrating $CD8^+$ T cell subsets in control and Sdc1-cKO mice bearing orthotopic tumors, with clustering executed as described in **Figure 4O**. (**I**) Experimental methodology employed to assess the pro-tumorigenic roles of $CD138^+$ TAMs involved the use of a KPC/Sdc1-cKO chimera mouse model. (**J**) Tumor incidence rates in spontaneous KPC (CTL) and KPC/Sdc1-cKO chimera (Sdc1-cKO) mice at 16 weeks of age. Tumor presence is confirmed by the observation of a macroscopic tumor upon necropsy. *p<0.05 by two-sided Fisher's exact test (**J**).

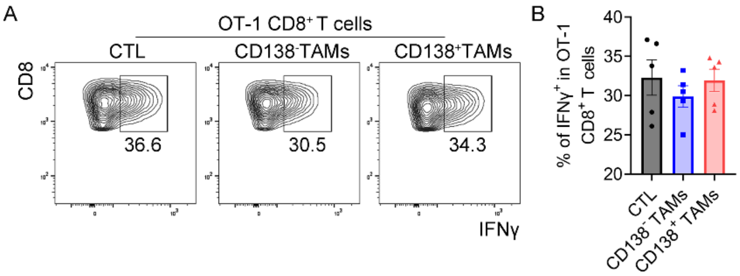

Supplemental Figure 11. $CD138^+$ TAMs do not directly impede the activation of $CD8^+$ T cells. (**A**) Representative images depicting the production of IFNγ by OT1 $CD8^+$ T cells from co-cultures of OT1 splenocytes with either $CD138^-$ or $CD138^+$ TAMs

sorted from orthotopic KPC mice. (**B**) Quantification of (**A**) (n=5 per group). Data represent mean ± SEM.

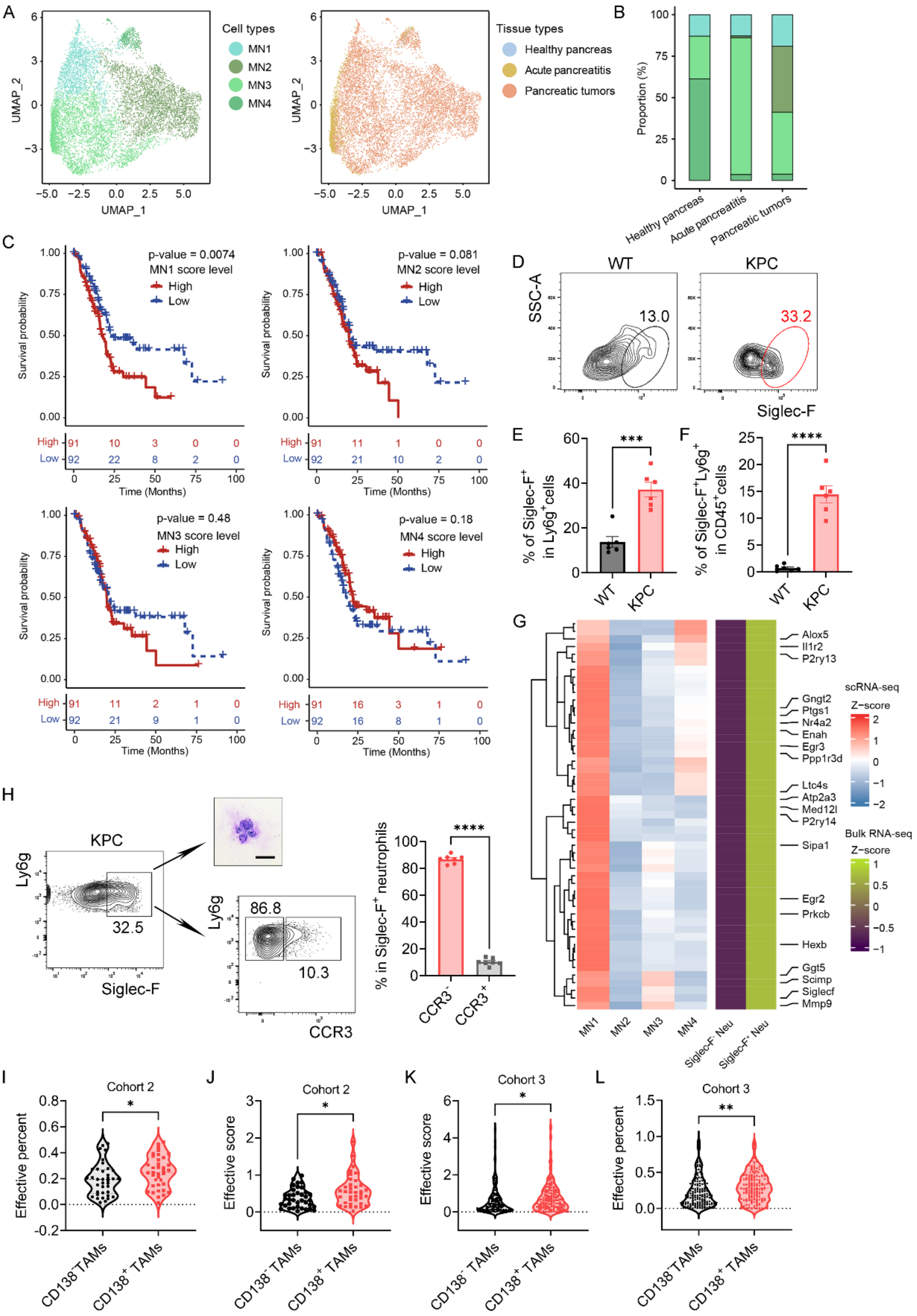

Supplemental Figure 12. A Siglec-F⁺ neutrophil subpopulation is expanded, co-

localized with CD138$^+$ TAMs, and is correlated with poor prognosis in PDAC. (**A**) UMAP plots depicting neutrophils in the pancreas of healthy and acute pancreatitis mice, as well as in orthotopic tumors, with colors representing scRNA-seq clusters (left) and the source of the cells (right). (**B**) Proportions of neutrophil subsets in the pancreas of healthy and acute pancreatitis mice, as well as in orthotopic tumors. (**C**) Survival probabilities of PDAC patients categorized into high- and low-score groups from the TCGA dataset, stratified by GSVA enrichment scores for each neutrophil subset. (**D**) Representative images depicting the presence of Siglec-F$^+$ neutrophils in orthotopic tumors. (**E** and **F**) Quantification of (**D**), revealing the frequencies of Siglec-F$^+$ neutrophils among Ly6g$^+$ cells (**E**) and CD45$^+$ cells (**F**) (n=6 per group). (**G**) Heatmaps illustrating the scaled expression of selected genes enriched in the gene set depicted in **Figure 5D**, across the neutrophil subsets shown in **Figure 5B**. (**H**) Images depicting the morphology and CCR3 expression levels of Ly6g$^+$Siglec-F$^+$ cells isolated from orthotopic tumors. Scale bar, 20μm. (**I**-**L**) Comparison of the effective score and percent between CD138$^-$ and CD138$^+$ TAMs in tumor tissues from PDAC patients in Cohort 2 (**I** and **J**, n=37 per group) and Cohort 3 (**K** and **L**, n=153-154 per group). The effective score and percent are utilized to estimate the relative spatial position between CD138$^+$ TAMs and SIGLEC-8$^+$ neutrophils. *$p<0.05$, **$p<0.01$, ***$p<0.001$, and ****$p<0.0001$ by unpaired t-test (**E**, **F**, and **H**-**L**). Data represent mean ± SEM.

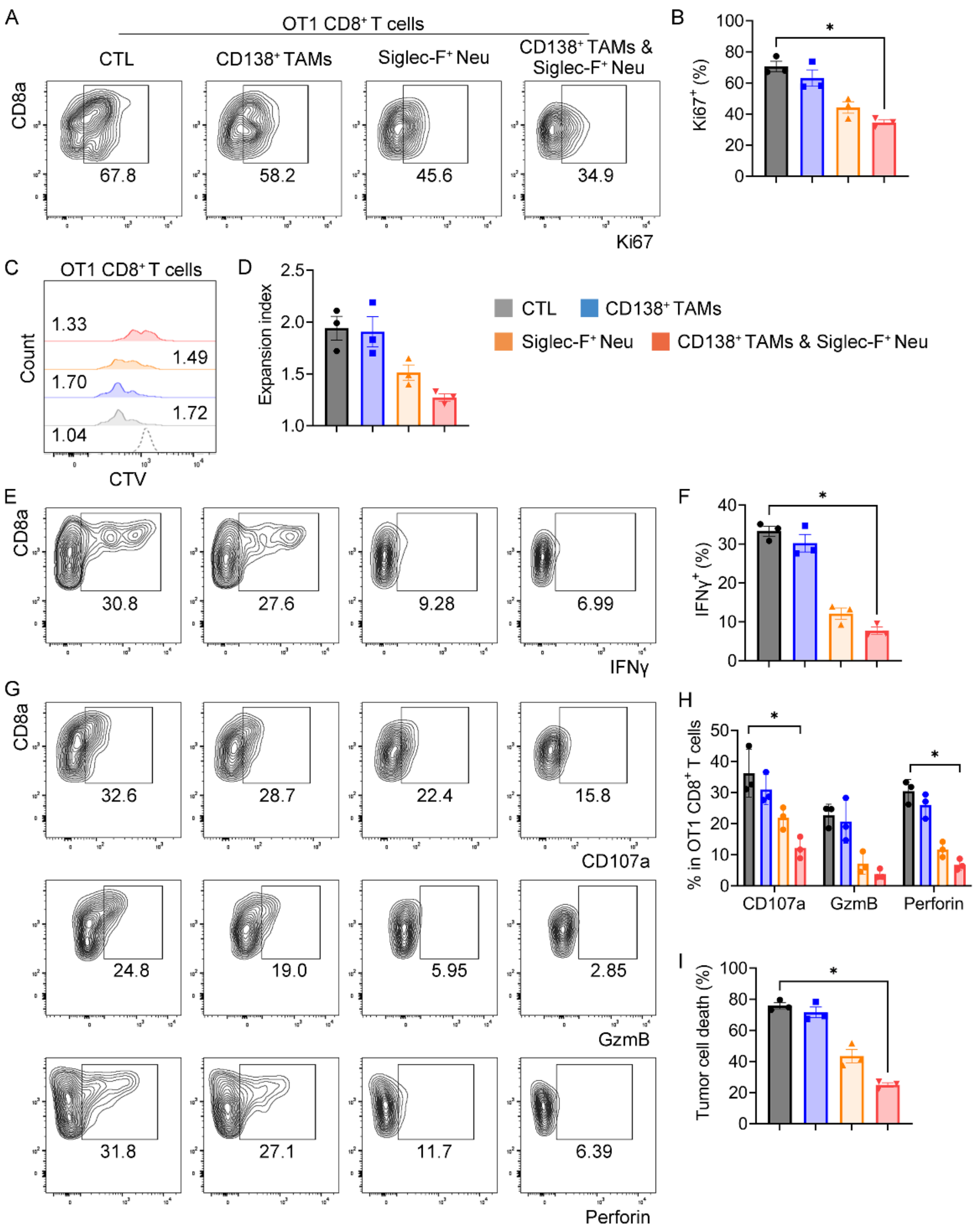

Supplemental Figure 13. CD138$^+$ TAMs and Siglec-F$^+$ neutrophils synergistically inhibit the activation of CD8$^+$ T cells. (**A**, **E**, and **G**) Plots depicting the presence of Ki67$^+$ (**A**), IFNγ$^+$ (**E**), and CD107a$^+$, GzmB$^+$, and Perforin$^+$ (**G**) cells among OT1 CD8$^+$ T cells derived from co-cultures of splenocytes from OT1 transgenic mice, with or without the inclusion of CD138$^+$ TAMs and/or Siglec-F$^+$ neutrophils sorted from orthotopic tumors. (**B**, **F**, and **H**) Quantification of (**A**), (**E**), and (**G**), respectively (n=3 per group). (**C** and **D**) Representative histogram (**C**) and the expansion index (**D**) of

CTV-labeled activated OT1 CD8$^+$ T cells further expanded for two days, either in the presence or absence of CD138$^+$ TAMs and/or Siglec-F$^+$ neutrophils derived from orthotopic tumors (n=3 per group). The dashed line indicates the OT1 CD8$^+$ T cells that are not activated by the OVA peptide. (**I**) Percentage of dead KPC-OVA cells in co-cultures with OT1 CD8$^+$ T cells, with or without the presence of CD138$^+$ TAMs and/or Siglec-F$^+$ neutrophils isolated from orthotopic tumors (Tumor: OT1: TAMs: Neu=1:5:1:5, n=3 per group). *p<0.05 by Kruskal-Wallis test with Dunn's multiple comparison test (**B**, **F**, **H**, and **I**). Data represent mean ± SEM.

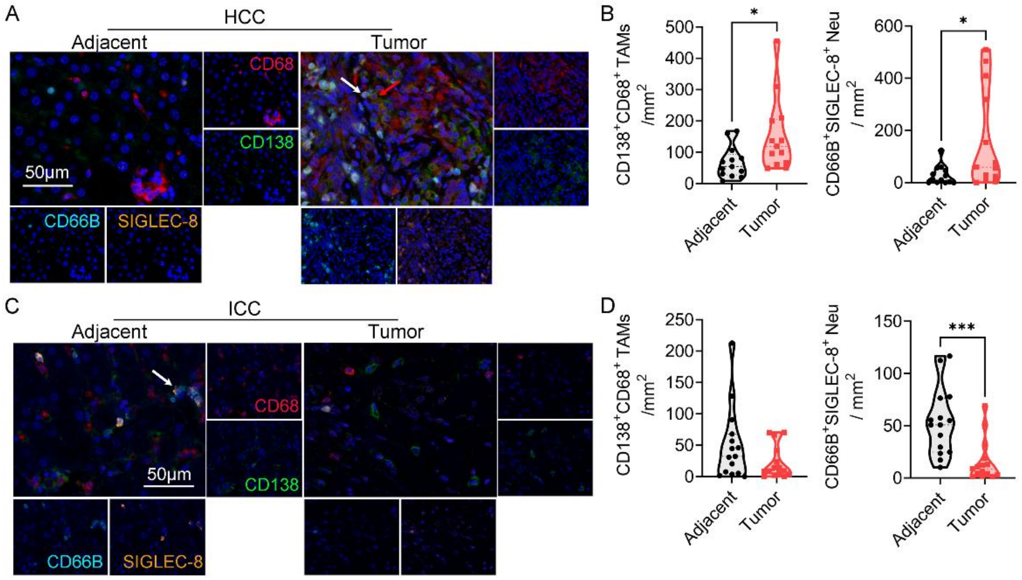

Supplemental Figure 14. CD138$^+$ TAMs and SIGLEC-8$^+$ neutrophils are present in tumor tissues from patients with HCC, but not in those with ICC. (**A** and **C**) Representative mIHC images illustrating the presence of CD138$^+$ TAMs (red arrow) and SIGLEC-8$^+$ neutrophils (white arrow) in paired adjacent benign and tumor tissues from HCC (**A**) and ICC (**C**) patients. (**B** and **D**) Quantitative analysis of (**A**) and (**C**), revealing the abundance of these two myeloid cell subsets in HCC (**B**, n=13 per group)

and ICC patients (**D**, n=15 per group). *p<0.05 and ***p<0.001 by paired t-test (**B** and **D**).

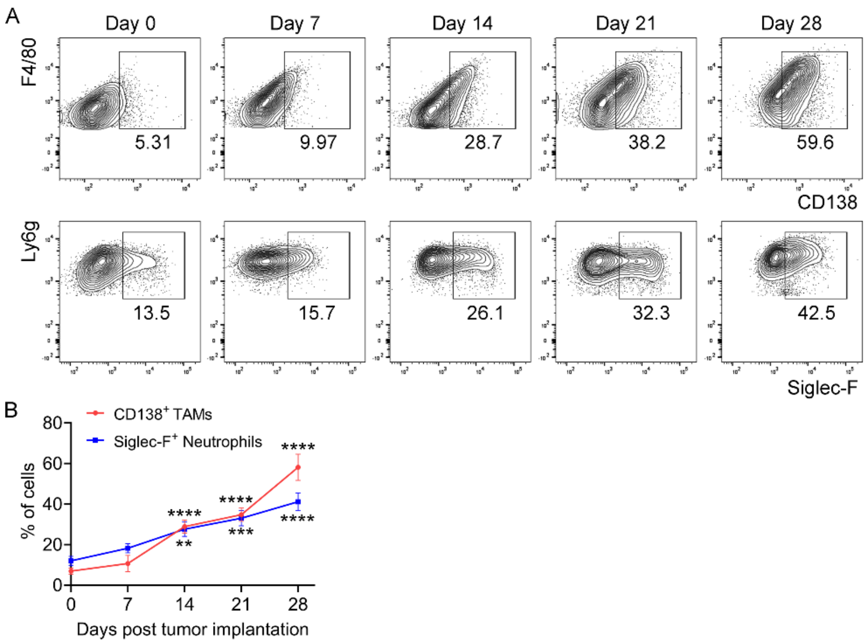

Supplemental Figure 15. CD138$^+$ TAMs and Siglec-F$^+$ neutrophils are observed in orthotopic tumors fourteen days after tumor implantation. (**A**) Frequencies of CD138$^+$ TAMs and Siglec-F$^+$ neutrophils measured at various timepoints following tumor implantation. (**B**) Quantification of (**A**) (n=6-7 per group). **p<0.01, ***p<0.001, and ****p<0.0001 by unpaired t-test (**B**). Data represent mean ± SEM.

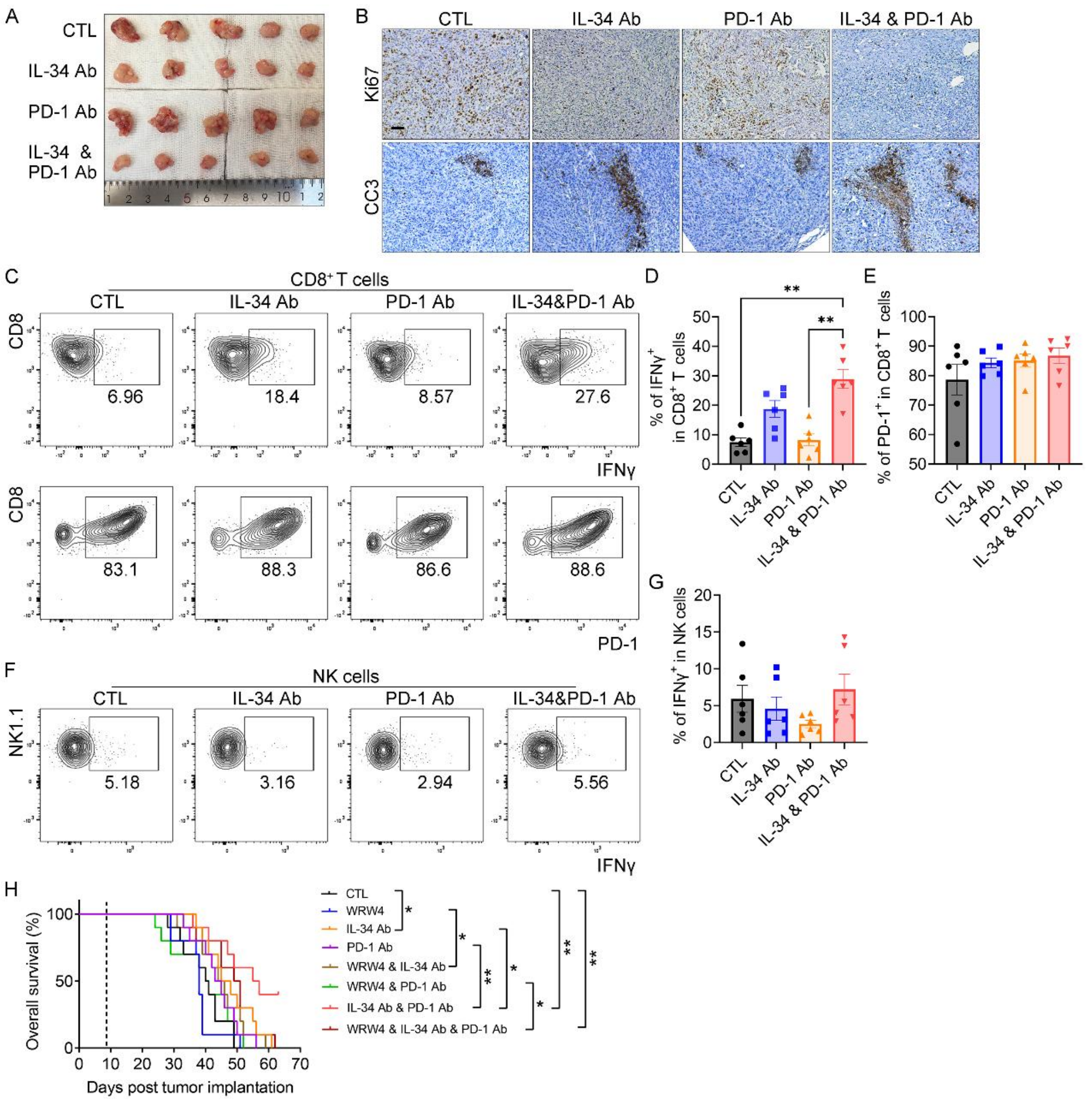

Supplemental Figure 16. The combination therapy involving anti-IL-34 and anti-PD-1 antibodies effectively halts tumor progression in PDAC by reinvigorating CD8$^+$ T cells. (**A**) Representative images of harvested tumor tissues from mice subjected to the combination treatment, as outlined in **Figure 8A**. (**B**) Immunohistochemical microscopy images revealing Ki67$^+$ cells and CC3$^+$ regions within tumor tissues from mice after the combination treatment. (**C**) Flow cytometric images showing the presence of IFNγ$^+$CD8$^+$ and PD-1$^+$CD8$^+$ T cells in orthotopic tumors following the combination therapy. (**D** and **E**) Quantification of (**C**) (n=6 per group). (**F**) Images illustrating IFNγ production in NK cells within the tumor tissues of

mice that underwent the combination treatment. (**G**) Quantification of (**F**) (n=6 per group). (**H**) Overall survival probabilities of mice following the combination therapy involving WRW4, anti-IL-34 antibodies, and/or anti-PD-1 antibodies (n=10 per group). The dashed line indicates the timepoint when the combination therapy commenced. *p<0.05 and **p<0.01 by Kruskal-Wallis test with Dunn's multiple comparison test (**D**) and by log-rank analysis (**H**). Data represent mean ± SEM.

Supplemental Tables

Supplemental Table 1. A negative correlation exists between the abundance of $CD138^+$ TAMs and tumor differentiation in patients with PDAC.

**Characteristic**	**$CD138^+$ TAM-low (n=86)**	**$CD138^+$ TAM-high (n=66)**	**P-value**
Age at diagnosis (y)			
Median (range)	65.5 (35-90)	65 (43-79)	0.528
Mean ± SD	65.6 ± 9.8	64.3 ± 9.2	
Sex			
Male	50 (58)	37 (56)	0.797
Female	36 (42)	29 (44)	
pT Stage			
T1	13 (15)	15 (23)	0.072
T2	36 (42)	36 (54)	
T3	33 (38)	13 (20)	
T4	4 (5)	2 (3)	
pN Stage			
N0	37 (44)	32 (48)	0.544
N1/N2	48 (56)	34 (52)	
pM Stage			

M0	79 (92)	64 (97)	0.329
M1	7 (8)	2 (3)	
Pathology			
Stage I	19 (22)	25 (38)	0.053
Stage II	55 (64)	33 (50)	
Stage III	5 (6)	6 (9)	
Stage IV	7 (8)	2 (3)	
CA19-9 (U/ml)			
Median (range)	377.5 (2-12000)	508.8 (6.2-12000)	0.47
Mean ± SD	2169.8 ± 3768.6	2161.5 ± 3544.9	
CA125 (U/ml)			
Median (range)	21.0 (4.6-266.6)	18.4 (4.8-538.1)	0.187
Mean ± SD	36.7 ± 42.2	34.4±69.6	
CEA (ng/ml)			
Median (range)	3.8 (0.9-51.2)	3.1 (0.7-258.5)	**0.015**
Mean ± SD	7.7 ± 8.8	8.7 ± 32.5	
Tumor location			
Head/Neck	62 (74)	50 (76)	0.88
Body/tail	22 (26)	16 (24)	

Primary tumor size (cm)			
Median (range)	3.5 (1.0-7.0)	2.8 (1.0-7.0)	0.066
Mean ± SD	3.5 ± 1.3	3.1 ± 1.3	
Perineural invasion			
Yes	55 (64)	50 (77)	0.086
No	31 (36)	15 (23)	
Blood vessel invasion			
Yes	38 (44)	33 (51)	0.422
No	48 (56)	32 (49)	
Tumor differentiation			
Low	28 (34)	18 (29)	**0.046**
Med	38 (46)	40 (63)	
High	17 (20)	5 (8)	

CEA, carcinoembryonic antigen.

Supplemental Data Files

Supplemental Data file 1. Patient information for Cohort 3.

Supplemental Data file 2. Marker genes of clusters of F4/80$^+$ cells isolated from orthotopic tumors.

Supplemental Data file 3. Marker genes of main clusters of live cells derived from the pancreas of both healthy and acute pancreatitis mice.

Supplemental Data file 4. Signaling pathways enriched in *Sdc1*$^+$ TAMs identified by GESA.

Supplemental Data file 5. The DEGs and the KEGG enriched pathways associated with CD138$^+$ macrophages derived from orthotopic tumors, in comparison to previously reported gene sets and pathways.

Supplemental Data file 6. Common genes identified between the DEGs of the *Sdc1*$^+$ (MM1) scRNA-seq cluster and CD138$^+$ macrophages isolated from orthotopic tumors.

Supplemental Data file 7. Marker genes of main clusters of live cells isolated from peripheral blood and tumor tissues of mice with KPC orthotopic tumors.

Supplemental Data file 8. Synergized genes up-regulated by IL-34 and PGE_2.

Supplemental Data file 9. Marker genes of clusters of monocytes/macrophages derived from peripheral blood and tumor tissues of orthotopic KPC mice.

Supplemental Data file 10. Driver genes of the monocyte-to-*Sdc1*$^+$ TAM transition.

Supplemental Data file 11. Signaling pathways enriched in *Sdc1*$^+$ TAMs based on KEGG enrichment analysis.

Supplemental Data file 12. Marker genes of CD8$^+$ T cell clusters, DEGs of CD8$^+$ T cells,

and GSEA of pathways enriched in $CD8^+$ T cells in orthotopic KPC mice following adoptive transfer.

Supplemental Data file 13. Marker genes of $CD8^+$ T cell clusters, DEGs of $CD8^+$ T cells, and GSEA of pathways enriched in $CD8^+$ T cells in control and Sdc1-cKO mice with orthotopic tumors.

Supplemental Data file 14. Marker genes of clusters of neutrophils derived from tumor tissues of orthotopic KPC mice.

Supplemental Data file 15. DEGs of Siglec-F^+ neutrophils compared to Siglec-F^- neutrophils and signaling pathways enriched in Siglec-F^+ neutrophils as determined by KEGG enrichment analysis.
