# Supplementary figures and images for: A CD138+ tumor-associated macrophage/Siglec-F+ neutrophil feed-forward loop promotes immune evasion in pancreatic cancer
Source: J Clin Invest. 2026 Mar 12;136(9):e199516. doi: 10.1172/JCI199516 (PMC13132371; doi:10.1172/JCI199516)

Full unedited blot for Supplemental Figure 7J.

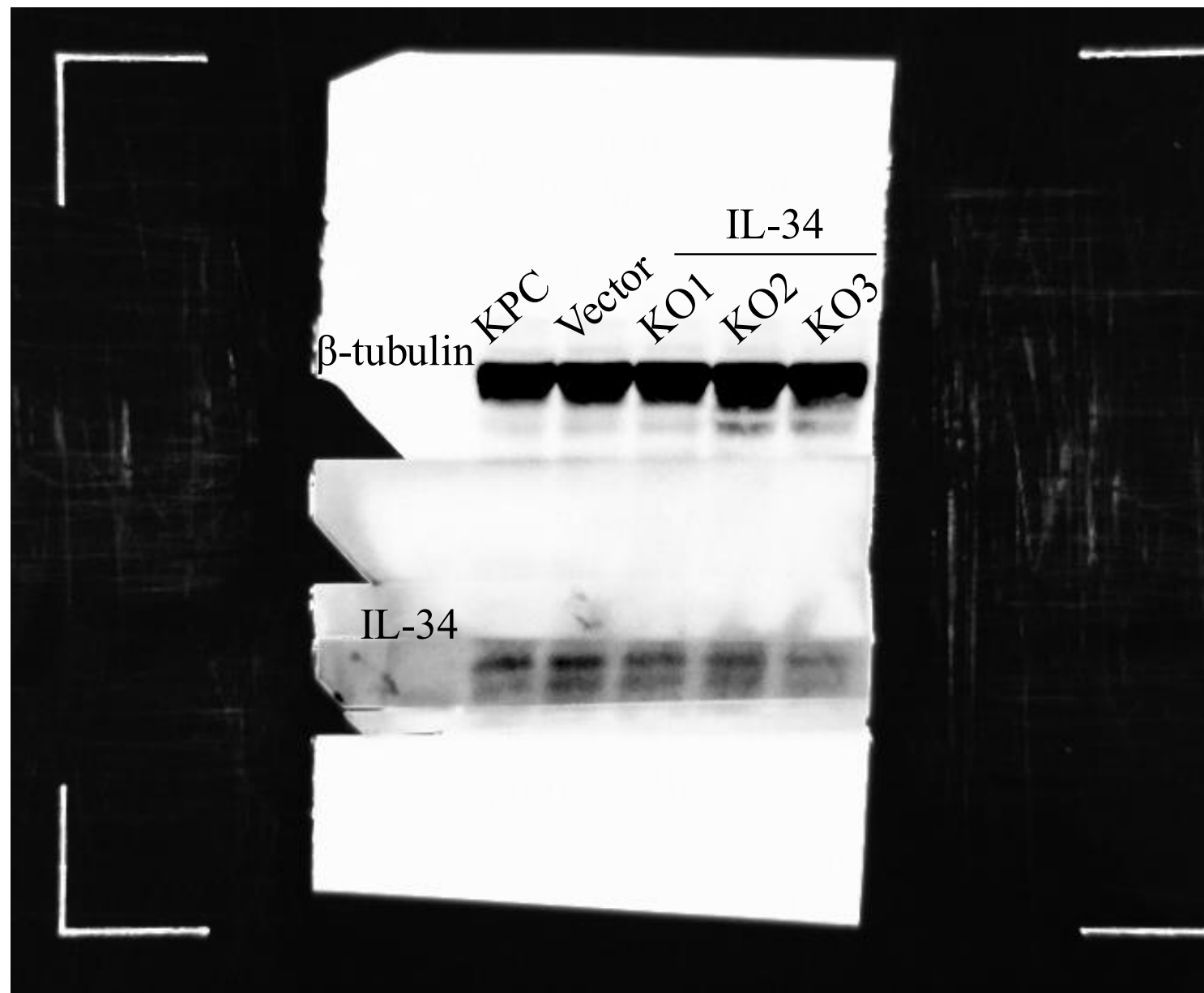

Full unedited blot for Supplemental Figure 7K.

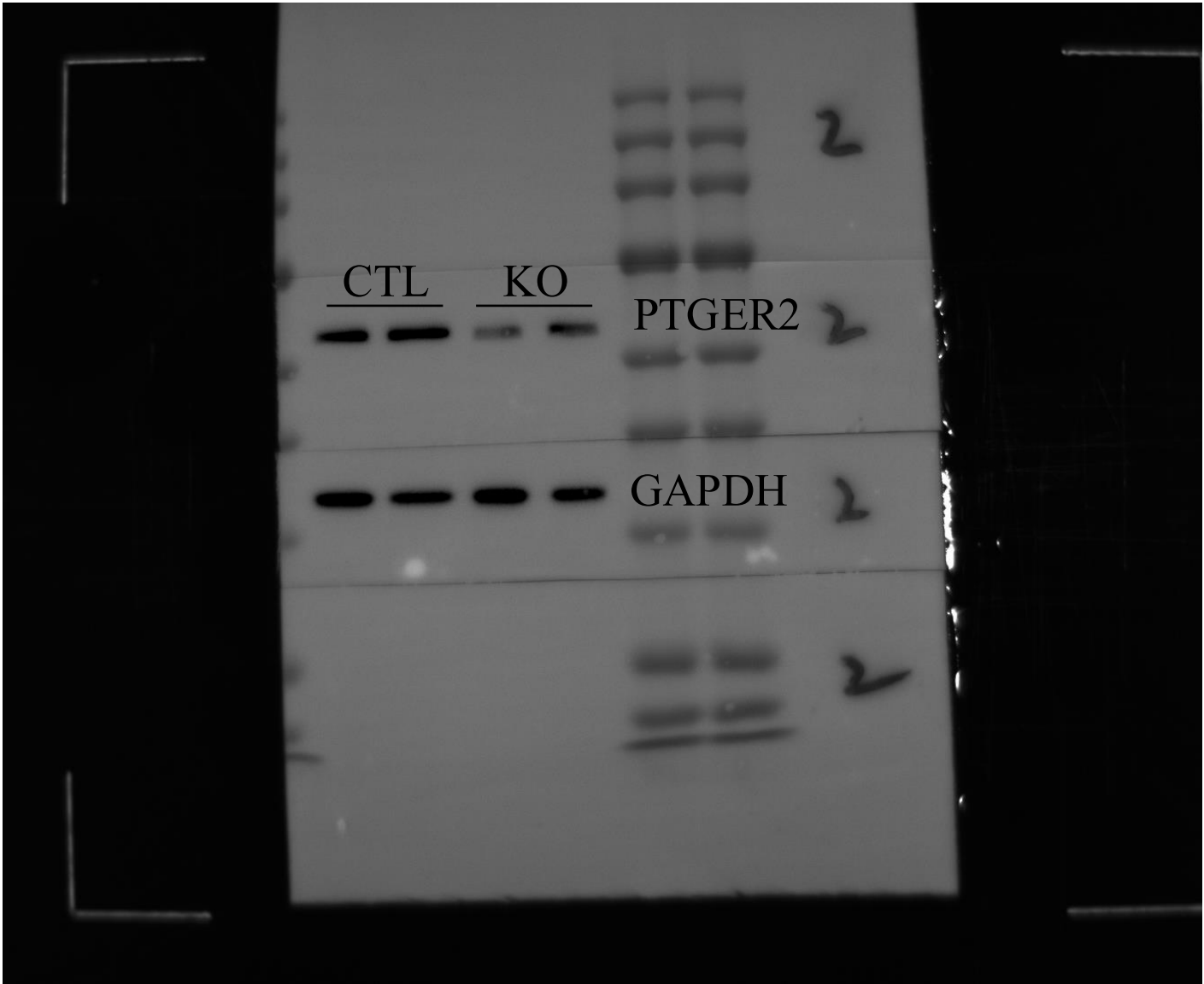

Supplement: Unedited blot and gel images [file jci-136-199516-s207.pdf]
